# Understanding hoarding behaviours in the context of homelessness

**DOI:** 10.1111/bjc.70045

**Published:** 2026-03-17

**Authors:** Tiago C. Zortea, Fiona Symington, Alasdair Churchard, Paul M. Salkovskis

**Affiliations:** ^1^ Department of Experimental Psychology, The Oxford Institute of Clinical Psychology Training & Research University of Oxford Oxford UK; ^2^ Emergency Department Psychiatric Service John Radcliffe Hospital, Oxford Health NHS Foundation Trust Oxford UK

**Keywords:** harm avoidance, hoarding, homelessness, housing and accommodation, material deprivation

## Abstract

**Objectives:**

To examine cognitive beliefs associated with hoarding in the context of homelessness, focusing on fear of future material deprivation, and to compare housing‐related adversities across groups with hoarding difficulties, homelessness or both.

**Design:**

Cross‐sectional, three‐group comparative study.

**Methods:**

Adults in the UK/Ireland were recruited between July 2023 and April 2024 through third‐sector and clinical partners via online, postal and in‐person routes. Participants were grouped as hoarding difficulties with homelessness in the past 10 years (H&H; *n* = 47), hoarding difficulties without homelessness (HD; *n* = 43) and homelessness without hoarding difficulties (HM; *n* = 39). Hoarding difficulties were identified by professional referral and eligibility screening, mapping onto DSM‐5 criteria (self‐report endorsement of all criteria). Primary outcomes were Beliefs About Hoarding Questionnaire–Revised (BAH) subscales (fear of material deprivation, harm avoidance, attachment disturbance). Secondary outcomes included negative housing experiences, hoarding severity (SI‐R), depression (PHQ‐8), anxiety (GAD‐7), functioning (WSAS) and early material deprivation (EEMD). Mixed‐model ANOVA tested group differences in BAH subscales, and Kruskal–Wallis tested housing experiences.

**Results:**

A significant group‐by‐belief interaction emerged. Fear of material deprivation and attachment disturbance were higher in H&H and HD than in HM. Harm avoidance was highest in H&H. H&H reported more negative housing experiences than HD and did not differ from HM (H&H ≈ HM > HD). Exploratory analyses showed higher depression, anxiety and impairment in H&H. HD showed the greatest hoarding severity (SI‐R total, clutter, discarding), whereas excessive acquisition was similar in H&H and HD.

**Conclusions:**

Hoarding difficulties in homelessness contexts appear embedded in cumulative psychosocial vulnerability rather than explained by deprivation alone. Harm avoidance may be particularly salient when hoarding co‐occurs with homelessness, suggesting potential targets for formulation and intervention in homelessness services.

**Practitioner points**

**Implications for practice**

- Hoarding difficulties among people who have experienced homelessness appear to be embedded in cumulative psychosocial vulnerability (e.g., heightened distress, impaired functioning), suggesting that interventions should extend beyond addressing material deprivation alone.
- Harm avoidance beliefs may be particularly salient when hoarding co‐occurs with homelessness, indicating the value of formulations that explicitly assess safety‐seeking functions of possessions and threat sensitivity.
- Clinicians working in homelessness and supported accommodation services may benefit from integrating multi‐informant assessments (service observations, self‐report, clinical formulation) to distinguish maladaptive hoarding from adaptive saving under conditions of scarcity.
- Effective intervention is likely to require personalized, longer‐term approaches that prioritize trust, stability and tenancy sustainment rather than brief or narrowly symptom‐focused treatments.

**Cautions and limitations**

- Hoarding difficulties were identified through professional referral and self‐report screening rather than structured diagnostic interviews with visual clutter assessment by the researchers, limiting diagnostic certainty.
- Cross‐sectional design precludes causal inference regarding the directionality of relationships between hoarding, homelessness and cognitive beliefs.
- Attrition was substantial, reflecting the instability of the population; findings may not generalize to individuals with the most chaotic or transient circumstances.
- Some hoarding‐related behaviours in homelessness contexts may reflect pragmatic responses to resource scarcity rather than clinically maladaptive hoarding.

## INTRODUCTION

Hoarding difficulties (HD) can be characterized as the end result of behaviours, including some combination of excessive acquisition and difficulties discarding, which result in great distress, clutter and the rendering of living space unavailable for its usual purpose (American Psychiatric Association, 2013; Frost & Hartl, 1996). The latest evidence on the prevalence of hoarding estimates that approximately 2 in every 100 people in the general population meet the criteria for the disorder (2.5%), an estimate that seems to be consistent across developed countries and similar for both males and females (Postlethwaite et al., 2019).

Hoarding is not only a source of pronounced psychological suffering (Gordon et al., 2013), but has also been found to be associated with additional complications including life‐threatening problems such as high risk of fire (Kysow et al., 2020), medical conditions (Bates et al., 2021), suicide risk (Archer et al., 2019), pests infestation, food poisoning (Frost et al., 2000), social isolation and loneliness (Yap et al., 2020) evictions, homelessness (Rodriguez et al., 2012) and a range of other severe problems (Tolin et al., 2008). Gordon et al. (2013) suggest that excessive clutter and emotional difficulties discarding may be better conceptualized as a ‘convergent pathway’, with the problematic accumulation of possessions resulting from several different experiences, beliefs and consequent behaviours. These include (1) harm avoidance, when objects are retained in order to prevent harm to the individual or others and to avoid the possibility of making an irreversible mistake; (2) attachment disturbance, developed in the fear that being alienated from an item will result in severe personal loss, as the object is considered as emotionally important to the individual due to its links with other people; and (3) fear of future material deprivation with respect to possessions, which reflects concerns about lacking resources or being unable to meet future needs, so that items are saved to protect against such an eventuality. Whereas harm avoidance centres on preventing immediate or potential negative outcomes arising from discarding a specific item, fear of future material deprivation reflects a broader anticipatory concern about scarcity or hardship. This latter set of beliefs may include past experiences of homelessness, and there is emerging evidence of the relationship between hoarding and homelessness (Greig et al., 2020; Tsai & Huang, 2019).

Homelessness is also the outcome of ‘convergent pathways’. Individuals experiencing homelessness comprise some of the most disadvantaged populations in society in most domains of life. It is defined by the United Nations as a condition in which individuals live on streets, in open spaces or cars, temporary emergency accommodation [e.g., couch surfing], shelters, camps or other temporary accommodation provided to those internally displaced, refugees or migrants. It also includes living in severely inadequate and insecure housing, such as residing in informal settlements. Rough sleeping is thus only one manifestation of homelessness, but not necessarily the most frequent one (United Nations Human Rights Office of the High Commissioner, 2021).

Like hoarding, homelessness is a convergent outcome, with having no permanent place of residence being the result of multiple elements, involving internal, systemic and external factors, including hoarding issues. Epidemiological data from the Northeast region of the US suggest that 18.5% of those experiencing homelessness living in supported housing are affected by hoarding issues as identified through the Clutter Image Rating (CIR), a validated visual screening tool used in quarterly room‐based welfare checks, more than three times the prevalence reported in the general population (Greig et al., 2020). In this context, ‘affected’ refers to residents whose bedroom or single‐room occupancy (SRO) space was rated at CIR ≥4, indicating clinically significant hoarding behaviour rather than a formal diagnosis of HD. Importantly, although HD requires clutter in a living space, residents in supported accommodation typically have a consistent private room (bedroom or SRO), enabling reliable assessment of *hoarding‐related clutter* within this population.

In addition to hoarding, findings from a recent meta‐analysis (Nilsson et al., 2019) suggest that several life events are associated with increased risk of homelessness, including physical abuse, foster care experiences, history of incarceration, drug misuse, suicide attempts and psychiatric problems. Recent data estimate that those experiencing homelessness present a remarkably higher prevalence of psychosis (21.21%) (Ayano et al., 2019) when compared to the general population. Lifetime prevalence of *one or more* adverse childhood experiences (ACEs; e.g., abuse [physical, sexual and emotional], neglect, exposure to domestic violence and other household problems) among homeless adults was 89.8%, and the lifetime prevalence *of four or more* ACEs was 53.9% (Liu et al., 2021). These numbers are strikingly higher than the global general population, for which the prevalence varies from 38 to 39% for *one or more* ACEs and 3–5% for *four or more* (Liu et al., 2021).

Hoarding can interact with homelessness in several ways, such as hoarding behaviour resulting in breach of tenancy conditions and consequent eviction, loss of uninsured property due to fire, repossession and eviction where payments are not made towards rent or loans on property and so on. There is evidence that people with HD and a history of homelessness (for various reasons, including eviction due to hoarding) are at great risk of experiencing chronic homelessness due to their record of default/eviction and future homelessness by eviction (Kerman et al., 2023; Tolin et al., 2008). For those working with individuals receiving support through temporary accommodation, hoarding behaviour can be an enormous challenge given the risks imposed not only on the individuals who hoard but also on other service users and health/social workers. The psychological processes involved in the interaction between homelessness and hoarding are yet to be evaluated.

It has been suggested that early experiences of material deprivation may be related to the development of beliefs which lead to hoarding behaviour (Seaman et al., 2010). The individual's early experience of substantial loss of belongings may be associated with an intense apprehension that this may recur, so possessions are acquired and kept to prevent such an eventuality (Gordon et al., 2013). Although this is a plausible hypothesis, there is mixed evidence on the relationship between hoarding behaviour and early experiences of material deprivation. Previous research has suggested that there was no significant difference between hoarders and non‐hoarders in terms of experiencing financial hardship, food scarcity, insufficient clothing, or inadequate housing throughout their lives (Frost & Gross, 1993). In fact, the vast majority of individuals from all comparison groups (hoarding disorder without comorbid OCD, hoarding disorder with comorbid OCD, OCD without hoarding symptoms and non‐clinical controls) investigated by Landau et al. (2011) had faced financial difficulties at some point, suggesting that financial hardship does not uniquely predispose individuals to hoarding behaviours. In their data, approximately 20% of individuals with hoarding issues reported inadequate housing at some stage in their lives, due to a variety of reasons including eviction, homelessness, or residing in subpar living conditions. This was in contrast to none of the participants with OCD but without hoarding behaviours, and only 5% of those from the general population without clinical conditions.

The latest research by Walji and Salkovskis (2024) compared three groups (individuals with hoarding reporting early material deprivation, individuals with hoarding reporting no early material deprivation and community controls) and found no evidence of different levels between the two hoarding groups for fear of material deprivation, harm avoidance and attachment disturbance (Gordon et al., 2013). Interestingly, fear of material deprivation was associated with hoarding behaviours regardless of whether there was any experience of material scarcity in early life. For those experiencing both hoarding behaviour and homelessness in adult life, proximity of the experience may, however, have an impact on such beliefs. This distinction is important because previous research has primarily operationalized deprivation as early‐life material hardship, whereas homelessness more often entails adult life and sometimes abrupt loss of housing, possessions, stability and control. In the present study, early material deprivation was measured explicitly (EEMD), while homelessness history and related housing adversities were used to index more proximal experiences of loss and insecurity. On this basis, we expected that such proximal housing disruption could engage threat‐ and scarcity‐related cognitive systems differently from early deprivation, potentially amplifying fear of material deprivation.

It thus may be that hoarding in the context of experience of homelessness could be driven by cognitions around the dread of further material deprivation. Tolin et al. (2008) reported that between 8 and 12% of a large sample of participants experiencing hoarding difficulties had been evicted or threatened with eviction due to hoarding at some point in their lives. Landau et al. (2011) remark that what stands out in this specific type of material scarcity (unlike other forms such as financial, food, or clothing shortage) is the prevalent theme of loss, frequently abrupt, associated with these experiences of eviction. The experience of losing one's home is likely to evoke feelings of loss and a diminished sense of control over one's surroundings and belongings. Consequently, individuals might seek solace and security in their belongings, especially after they have found a new place and created a ‘safe‐haven’ for themselves (Cooke, 2017; Landau et al., 2011).

Clinicians and support organizations working with homelessness face difficulties in accessing evidence‐based insights into the role hoarding plays within this group. There is a lack of understanding of the psychological processes involved, which hinders the creation of effective intervention models and strategies likely to succeed. In particular, little is known about how the core cognitive mechanisms associated with hoarding (Gordon et al., 2013) operate when individuals have also experienced homelessness, despite both phenomena being linked to vulnerability, material loss and heightened threat anticipation. Understanding whether specific beliefs (such as fear of material deprivation) are amplified in this context may help clarify why hoarding behaviours persist or escalate after housing loss. Such knowledge is directly relevant for services developing targeted psychological interventions, risk‐management procedures and tenancy‐sustainment approaches for people in temporary or supported accommodation.

The current study seeks to fill this gap, providing preliminary evidence on the cognitive processes that may underpin hoarding within homelessness contexts and thereby offering a theoretically informed basis for future intervention design. It tests the hypotheses that: (1) individuals experiencing hoarding and homelessness would report significantly higher levels of fear of material deprivation relative to those experiencing hoarding or homelessness only; this hypothesis follows from evidence suggesting that loss of possessions, housing disruption, and material insecurity can intensify beliefs about future scarcity. Accordingly, we focused specifically on fear of material deprivation, as it is the cognitive belief most theoretically aligned with the anticipated impact of recent and proximal losses associated with homelessness, in contrast to other hoarding‐related beliefs (harm avoidance, attachment disturbance) that do not show the same conceptual link to the experience of losing one's home or belongings. (2) those with experiences of hoarding and homelessness would report more negative experiences related to housing and accommodation relative to the other groups. Although we did not include a separate scale of adult material deprivation, homelessness and associated housing disruption were conceptualized as a proximal marker of material loss and insecurity.

## METHOD

### Study design

This study employed a cross‐sectional design with three groups of people: those with current hoarding difficulties who experienced homelessness within the past decade (H&H); those with hoarding issues but no history of homelessness (HD) and those with history/current experiences of homelessness but no hoarding difficulties. (HM). The primary variable was participants' responses on the three subscales (fear of future material deprivation, harm avoidance and attachment disturbance) of the Beliefs about Hoarding (BAH) instrument (Gordon et al., 2013) and between‐group differences in past experiences with housing and accommodation. Thus, the group to which the participant belonged represent the between‐subjects factor, with the measures being assessed (BAH subscales) as the within‐subjects factor.

### Sampling procedures

Data collection took place from 12 July 2023 and 19 April 2024 using (1) an anonymized online survey using Qualtrics XM Platform™ securely provided by the University of Oxford and (2) in‐person participation (through printed questionnaires). Participants were identified in collaboration with special interest groups on both hoarding and homelessness of multidisciplinary professionals across the country. These professionals included clinical psychologists, social workers, commissioners, policymakers, charities and service users. Front‐line staff and clinicians within these organizations identified individuals on their caseloads who, in their clinical and practical judgement, presented with marked hoarding‐related clutter and/or significant experiences of homelessness and invited them to take part in the study. For participants with hoarding difficulties, referral was therefore based on direct observation of clutter impairment or related safety/tenancy concerns rather than open self‐selection. Most of the in‐person data collection took place in homelessness shelters and temporary accommodation sites in the south of England. Participants were invited to contribute to the research project voluntarily and were given a thank‐you voucher (value of £20). The project received approval from the University of Oxford's Central University Research Ethics Committee (Reference: R86382/RE001).

Participants completing the online survey were also referred by collaborating professionals (e.g., clinicians, social workers, third‐sector organizations) rather than recruited through open social‐media advertising. Online, in‐person and postal recruitment were all used to maximize reach within a hard‐to‐engage population, and recruitment mode did not determine group allocation.

### Participants

The study sample comprised the three groups (H&H, HD, HM) and were eligible if they met the following inclusion criteria: (1) aged 18+, (2) fluent in English, (3) living in the UK or Ireland, (4) having experienced homelessness currently or within the past 10 years (groups H&H and HM) and (5) reporting significant hoarding symptoms (groups H&H and HD). Potential participants with hoarding difficulties were first identified by collaborating professionals (e.g., charities, clinicians and mental health workers in the third sector) who routinely support individuals experiencing hoarding‐related problems and who reported that visible clutter was causing significant distress, safety risks, or practical difficulties for the individual and/or the service.

Following referral, participants completed a brief screening questionnaire that directly mapped onto all DSM‐5 diagnostic criteria for Hoarding Disorder (American Psychiatric Association, 2013). Individuals were required to endorse every DSM‐5 criterion via self‐report (accumulation, difficulty discarding, clutter causing impairment, associated distress/impact and not attributable to another condition) to be eligible for the hoarding groups. Those who did not endorse all criteria were excluded. Because this process relied on self‐report and referrer judgement rather than a structured diagnostic interview or independent clinical assessment by the research team, we refer throughout to ‘hoarding difficulties’ rather than confirmed Hoarding Disorder.

Exclusion criteria comprised: (1) having a learning disability, (2) being under the effect of alcohol or other substances at the point of participation and (3) having a neurological condition that would impede full participation due to cognitive impairment. We did not systematically assess or exclude other mental health or medical diagnoses (e.g., psychosis, bipolar disorder, dementia, or major depressive disorder); instead, we recorded common symptoms of depression, anxiety and functional impairment using validated self‐report measures (see Measures) and relied on referrers' clinical judgement and exclusion criteria to ensure that participants were able to provide informed consent and valid responses.

The H&H group represents the intersection of hoarding difficulties and homelessness as factors influencing fear of future material deprivation and other hoarding belief, with the other groups allowing separation of these two factors.

In the absence of previous work, we sought to detect a moderate to small effect size in the analyses. The power analysis focused on our primary hypothesis, which employed a mixed‐model analysis of variance. Based on a Cohen's *f* effect size of .15 (between moderate‐small) with 85% power at an α level of .05, a total sample size of 102 participants (34 per group) was required to detect an interaction.

### Measures

In addition to demographic information and the primary analysis variables, the study included measures of common mental health difficulties, hoarding and early material deprivation to provide an understanding of participants' general mental health challenges and self‐reported past experiences. Table 1 outlines the psychometric properties of the continuous measures and the subsections below describe each instrument used in the study. Because eligibility for the hoarding groups was established through the two‐stage screening process described above (referrer identification and self‐report endorsement of all DSM‐5 criteria), the measures below were used to characterize symptom severity rather than to determine diagnostic status. No structured diagnostic interview (e.g., for Hoarding Disorder, psychotic disorders, or substance use disorders) was administered, and the research team did not conduct visual inspections of clutter; the study therefore relies on self‐report measures supplemented by collaborators' clinical judgement at referral, which should be considered when interpreting the findings.

**TABLE 1:** Psychometric properties of continuous measures employed in the study.

Measure	Whole sample (*N* = 129)				HD (*n* = 43)				HM (*n* = 39)				H&H (*n* = 47)			
	*α*	*SE*	95%CI		*α*	*SE*	95%CI		*α*	*SE*	95%CI		*α*	*SE*	95%CI	
			Lo.	Up.			Lo.	Up.			Lo.	Up.			Lo.	Up.
Beliefs about hoarding																
Harm avoidance	.81	.13	.75	.85	.71	.24	.54	.82	.79	.25	.65	.87	.81	.23	.71	.88
Fear of material deprivation	.87	.14	.83	.90	.86	.23	.79	.91	.82	.24	.71	.89	.84	.22	.76	.90
Attachment disturbance	.91	.13	.88	.95	.92	.23	.87	.95	.87	.24	.80	.92	.86	.78	.78	.91
Patient Health Questionnaire‐8	.85	.13	.80	.88	.84	.23	.75	.90	.85	.24	.76	.90	.82	.22	.72	.88
Generalized Anxiety Disorder‐7	.86	.13	.81	.89	.83	.23	.73	.89	.85	.25	.75	.90	.82	.22	.72	.88
Working and social adjustment	.74	.14	.66	.80	.76	.24	.61	.85	.74	.25	.57	.84	.69	.23	.51	.80
Savings‐Inventory Revised																
Excessive acquisition	.79	.13	.74	.84	.79	.23	.67	.87	.72	.25	.54	.82	.69	.22	.52	.80
Difficulty discarding	.90	.13	.87	.92	.79	.23	.66	.86	.84	.25	.54	.82	.79	.23	.66	.86
Cluttered living spaces	.93	.13	.92	.95	.89	.23	.83	.93	.84	.25	.54	.82	.90	.22	.85	.93
Early experiences of material deprivation	.93	.13	.91	.95	.87	.23	.80	.92	.93	.24	.88	.95	.94	.22	.92	.96

Abbreviations: H&H, Homelessness and hoarding group; HD, hoarding group; HM, Homelessness group; Lo. and Up., lower and upper bound values of 95% confidence interval; *SE*, standard error; *α*, Cronbach's alpha.

#### Demographic information

Demographic information was collected including age, gender, gender assigned at birth, sexual orientation, ethnicity, cohabitation, marital status, education level and working status. Based on participants screening information, we were able to ascertain their group membership (H&H, HD, or HM).

#### Beliefs about hoarding

The *Beliefs About Hoarding Questionnaire Revised*—BAH (Gordon et al., 2013) was our primary measure and it is a 28‐item self‐report measure designed to assess beliefs and experiences characteristic of hoarding difficulties. These include three subscales for hoarding motivated by harm avoidance, fear of material deprivation and attachment disturbance. Items are rated on a 7‐point Likert scale (from 1 = strongly disagree to 7 = strongly agree), with higher scores indicating stronger endorsement of the relevant belief domain. The harm avoidance subscale assesses beliefs that discarding items may lead to danger, mistakes, or negative outcomes; the attachment disturbance subscale captures the emotional significance ascribed to possessions and the distress associated with separation from them and the fear of material deprivation subscale measures anticipatory concerns about lacking resources or being unprepared for future needs. The BAH has demonstrated good internal consistency, convergent validity with established hoarding measures and sensitivity to clinical change, making it a widely used tool for assessing cognitive mechanisms associated with hoarding.

#### Depressive symptoms

*Patient Health Questionnaire 8*—PHQ8 (Kroenke et al., 2009) is an 8‐item self‐report measure of depressive symptoms—which does not include item 9 of the PHQ‐9 (on suicidal and self‐harm thoughts). Each item is rated on a four‐point scale from 0 (not at all) to 3 (nearly every day). Total scores range from 0 to 24, and previous studies demonstrate that total scores ≥10 indicate clinically relevant depressive symptoms (Kroenke et al., 2009).

#### Generalized anxiety symptoms

*Generalized Anxiety Disorder 7*—GAD7 (Spitzer et al., 2006) is a seven‐item self‐report measure employing a four‐point scale (same used in the PHQ‐8) for brief screening of severity of anxiety symptoms over the past two weeks.

#### Impairment in functioning

*Work and Social Adjustment Scale*—WSAS (Mundt et al., 2002) is a valid and dependable tool for assessing impaired functioning of five life domains: work, home management, social leisure activities, private leisure activities, family and relationships. Each of these domains is assessed via self‐reporting items ranging from 0 (not at all) to 8 (very severely). The wording of this tool's item 1 was adapted following the suggestions of a service user during a Patient and Public Involvement (PPI) session (a structured consultation with individuals with lived experience to inform study design and materials): ‘My problems make it difficult for me to work *or look for a job*’ instead of ‘My problems make it difficult for me to work’.

#### Experiences of hoarding

*Savings‐Inventory Revised*—SI‐R (Frost et al., 2004) is a 23‐item self‐report measure designed to assess the severity of the main symptoms of hoarding (clutter, difficulty discarding and excessive acquisition) and the distress and interference associated with each. Items are rated on a 5‐point Likert scale. The measure is made up of three subscales: excessive acquisition, difficulty discarding and cluttered living spaces. A sum of all items yields a total score for the measure, which ranges from 0 to 92. Reliability and validity have been established for the SI‐R (Frost et al., 2004). In the present study, SI‐R scores were used to characterize the severity and profile of hoarding‐related experiences across groups; they were not used in isolation to confer a formal diagnosis of Hoarding Disorder. The research team did not directly observe participants' living spaces or visually verify clutter levels. For participants in supported or temporary accommodation, however, identification by collaborating staff typically followed concerns about observable clutter and its impact on the safety or functioning of the accommodation.

#### Experiences of early material deprivation

*Experiences of Early Material Deprivation Questionnaire*—EEMD (Walji & Salkovskis, 2024) measures self‐reported levels of early experiences of material deprivation, including childhood poverty and access to essential possessions. This scale was developed based on the Early Financial Hardship Scale (Hezel & Hooley, 2014) and tailored with the input of individuals who have first‐hand experience with hoarding difficulties and homelessness. The instrument contains 12 items listing essential possessions where participants would rate from 0 = ‘I/we never struggled to obtain this’ to 4 = ‘I/we always struggled to obtain this’ for each item.

#### Past experiences of housing and accommodation

To estimate participants' prior experiences with housing and accommodation, we developed a checklist in collaboration with individuals who have current and past experiences of homelessness. The questionnaire includes nine items where participants indicate whether the stated experience applied to them (1) or not (0) (e.g., ‘I've lived in conventional housing that is unfit for human habitation’). A final score was obtained by summing the total number of experiences each participant had faced. This checklist was co‐developed with individuals with lived experience of homelessness during PPI sessions, and wording reflects terminology commonly used in UK housing and support services; during in‐person data collection, staff and researchers were available to clarify any items as needed.

### Data diagnostics and analytic strategy

**FIGURE 1:**
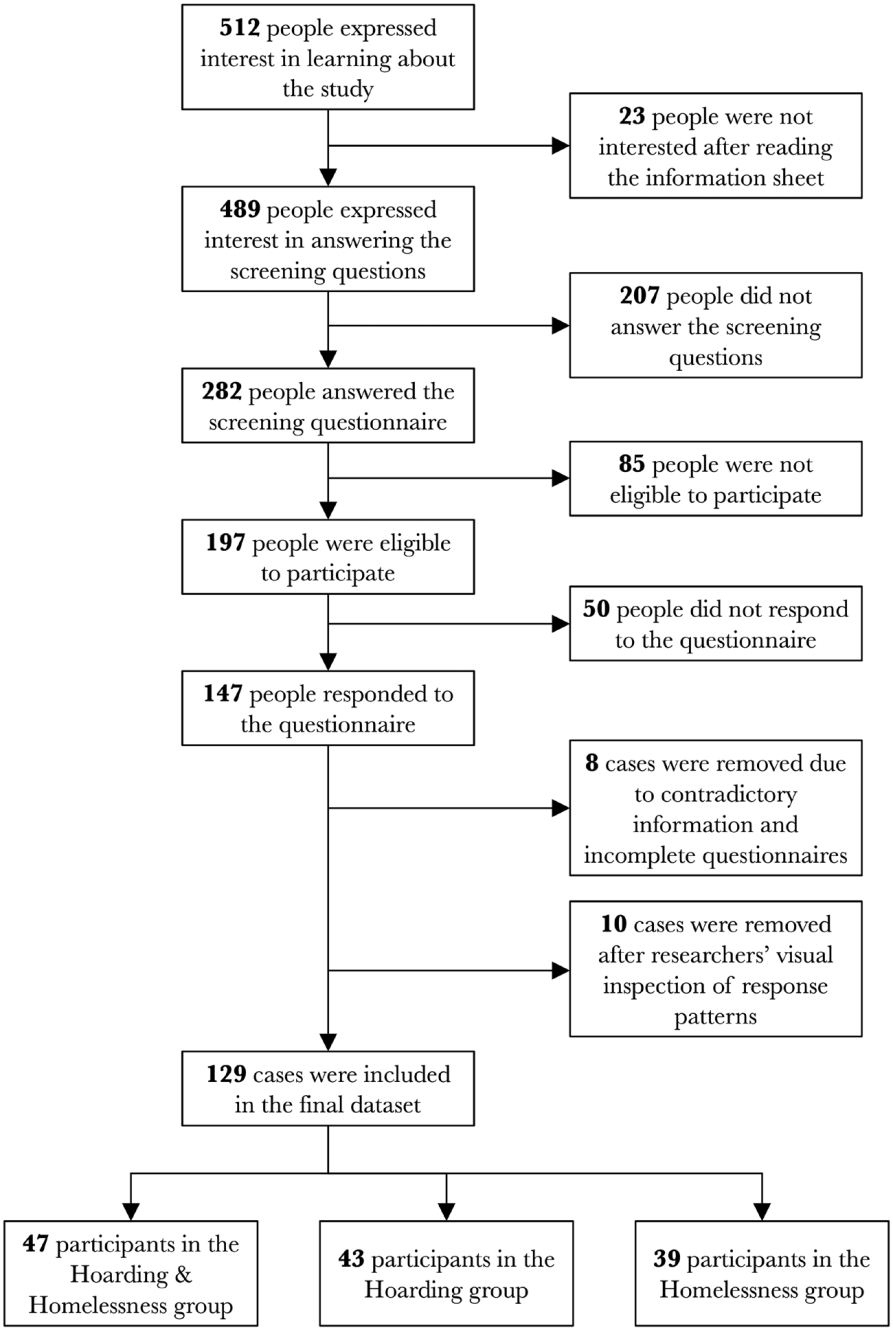
Participant flow and composition of the final dataset.

Following data collection, we conducted a series of diagnostic procedures to determine the final sample size. First, we matched information from the screening questionnaire with repeated questions about participants' homelessness experiences. Those with inconsistent answers to these questions were removed. Second, incomplete questionnaires were excluded, as participants only received compensation after completing all research activities, as stated in the information sheet and consent materials. Finally, to detect response bias (e.g., participants who responded with the same value throughout the entire questionnaire), we used the R package *responsePatterns* (Gottfried et al., 2022). This package employs an autocorrelation‐based algorithm to identify potential response biases. In total, 25 cases were flagged. The algorithm generated plots for each case's response pattern, which were then visually assessed by five independent researchers. Each researcher rated the inclusion or exclusion of each case based on the visual response pattern and provided a confidence level (0–100%) for their decision. The researchers reached a 90% overall agreement in their decisions, with an overall confidence level of 71.4% for excluded cases and 63.6% for included cases. A detailed flowchart of the final sample size composition is displayed in Figure 1 (Results section).

Data preparation and all psychometric, descriptive and inferential statistical analyses were conducted in R version 4.0.3 (2020‐10‐10) (R Core Team, 2020) via RStudio 2023.06.0 + 421. A complete and transparent reproducible code is available in the supplementary documents. Descriptive statistics, including frequencies, percentages, means and standard deviations, were calculated for all measures. To ascertain the demographic profile of each study group (H&H, HD, HM) and identify significant differences between them, we dichotomized categorical values (e.g., ‘heterosexual’ vs. ‘Other sexual orientations’) and employed the Chi‐Square test and Cramer's *V* effect size. Descriptive statistics for all levels of each categorical measure are fully reported in the supplementary materials.

Group mean comparisons for psychopathology variables (e.g., PHQ, GAD, WSAS, SI‐R) were conducted using analysis of variance (one‐way ANOVA) when assumptions of normality and homogeneity of variances (homoscedasticity) were met. If a statistically significant difference was identified, we followed up with Tukey HSD's post‐hoc tests for multiple comparisons. Games‐Howell post‐hoc correction test was employed when homoscedasticity was violated. When both assumptions were violated, we used the non‐parametric Kruskal–Wallis test with Dwass–Steel–Critchlow–Fligner pairwise median comparisons. To test our main hypothesis, we employed a mixed‐model analysis of variance following assumption checks and one‐way ANOVA to estimate simple main effects if the interaction were to be significant. For our second hypothesis, where data violated normality and homoscedasticity assumptions, we used the Kruskal‐Wallis test. The analysis plan for our hypotheses was pre‐registered with the University's PAS panel and at AsPredicted.org (#173686—available at https://aspredicted.org/e5cd4.pdf). All analyses beyond the hypotheses specified are exploratory.

## RESULTS

### Participants

From the start of recruitment, 512 people expressed interest in the study. Ultimately, data from 129 participants were included in the analysis: 47 in the H&H group, 43 in the HD group and 39 in the HM group. Figure 1 details the flow of participants throughout the recruitment process.

Overall, participants had a mean age of 45.3 years (*SD* = 13.5), ranging from 18 to 74 years. The sample consisted of 50.4% females (*n* = 65), 46.5% males (*n* = 60) and 3.1% non‐binary individuals (*n* = 4). Most participants identified as heterosexual (79.1%, *n* = 102) and white (76.7%, *n* = 99). Additionally, most participants were single/had no partner (58.1%, *n =* 75), shared a residence with someone (67.4%, *n* = 87), did not have a higher education degree (57.6%, *n* = 68) and were out of work (69%, *n* = 89). A more detailed description of participant demographics is available in the supplementary materials. Table 2 provides a breakdown of demographic information by study group, indicating any statistically significant differences between groups.

**TABLE 2:** Demographic and psychopathology information across study groups.

Measure	Whole sample (*N* = 129)	H&H (*n =* 47)	HD (*n =* 43)	HM (*n =* 39)	Between‐groups comparative statistics
Gender: *n* (%)					*χ* ^2^ (2, *N* = 129) = 43.8, ***p* < .0001**; Cramer's *V* = .597
Female	63 (51.2%)	18^a^ (40%)	37^b^ (92.5%)	8^a^ (20.5%)	
Male	60 (48.8%)	26^a^ (59%)	3^b^ (7.5%)	51^a^ (12.6%)	
Sexual orientation					*χ* ^2^ (2, *N* = 129) = 2.15, *p* = .341; Cramer's *V* = .129
Heterosexual	102 (79.1%)	34 (72.3%)	35 (81.4%)	33 (84.6%)	
Other	27 (20.9%)	13 (27.7%)	8 (18.6%)	6 (15.4%)	
Ethnicity					*χ* ^2^ (2, *N* = 129) = 1.87, *p =* .393; Cramer's *V* = .120
White	99 (76.7%)	33 (70%)	34 (79.1%)	32 (82.1%)	
Other	30 (23.3%)	14 (29.8%)	9 (20.9%)	7 (17.9%)	
Cohabitation status					*χ* ^2^ (2, *N* = 129) = 1.87, *p =* .393; Cramer's *V* = .036
Lives alone	42 (32.6%)	15 (31.9%)	15 (34.8%)	12 (30.7%)	
Shares residence	87 (67.4%)	32 (68%)	28 (65%)	27 (69.2%)	
Education					*χ* ^2^ (2, *N* = 129) = 12.68, ***p =*** .**001**; Cramer's *V* = .327
Higher education	50 (42.4%)	13^a^ (30.9%)	26^b^ (65%)	11^a^ (30.5%)	
Non‐higher education	68 (57.6%)	29^a^ (69%)	14^b^ (35%)	25^a^ (69.4%)	
Work status					*χ* ^2^ (2, *N* = 129) = 7.24, ***p =*** .**026**; Cramer's *V* = .237
Working	40 (31.0%)	11^a^ (23.4%)	20^b^ (46.5%)	9^ab^ (23%)	
Out of work	89 (69.0%)	36^a^ (76.5%)	23^b^ (53.4%)	30^ab^ (76.9%)	
Age: *M* (*SD*)	45.4 (13.5)	42.6^a^ (12.6)	53.2^b^ (12.8)	40^a^ (11.6)	*F*_(2,83)_ = 13.1, ***p* < .0001**.
Depressive symptoms	11.19 (5.83)	13.65^a^ (5.47)	10.67^b^ (5.32)	8.79^b^ (5.76)	*F*_(2,83)_ = 8.27, ***p* < .0001**.
Generalized anxiety	9.54 (5.33)	12.44^a^ (4.86)	7.67^b^ (4.53)	8.10^b^ (5.25)	*F*_(2,82)_ = 13.37, ***p* < .0001**.
Impaired functioning	17.95 (9.17)	20.93^a^ (8.91)	18.18^ab^ (8.34)	14.07^b^ (9.14)	*F*_(2,82)_ = 6.09, ***p* = .0033**.
Hoarding experiences					
Clutter	16.91 (9.94)	17.97^a^ (8.40)	24.58^b^ (6.49)	7.17^c^ (5.92)	*χ* ^2^ (2, *N* = 129) = 63.95, ***p <*** .**0001**; *ε* ^2^ = .499
Discarding	14.64 (7.33)	16.06^a^ (5.43)	19.93^b^ (4.16)	7.10^c^ (5.77)	*χ* ^2^ (2, *N* = 129) = 63.62, ***p <*** .**0001**; *ε* ^2^ = .497
Excess acquisition	13.09 (6.06)	15.14^a^ (5.43)	15.11^a^ (5.23)	8.38^b^ (5.01)	*χ* ^2^ (2, *N* = 129) = 32.87, ***p <*** .**0001**; *ε* ^2^ = .256
Hoarding (total)	44.65 (21.05)	49.19^a^ (17.05)	59.62^b^ (11.71)	22.66^c^ (14.61)	*χ* ^2^ (2, *N* = 129) = 63.29, ***p <*** .**0001**; *ε* ^2^ = .494
Early experiences of material deprivation	13.23 (11.49)	18.57^a^ (12.89)	9.23^b^ (7.94)	11.10^b^ (10.70)	*χ* ^2^ (2, *N* = 129) = 14.07, ***p =*** .**0008**; *ε* ^2^ = .110

*Note*: Values with differing superscript letters differ significantly (*p* < .05). Bold values highlight statistically significant differences (*p* < 0.05).

### Psychopathology and experiences of material deprivation

Significant differences were found among the groups in several areas. The HD group was, on average, older than the H&H and HM groups. The HD group also had a higher number of individuals with a higher education degree. In terms of mental health, the H&H group reported higher levels of depressive symptoms and generalized anxiety symptoms compared to the other groups. The H&H group also showed higher levels of impaired functioning than the HM group, but there was no significant difference in the level of impaired functioning between the H&H and HD groups. The HD group exhibited significantly higher levels of overall hoarding symptoms than the other groups, with the H&H group scoring higher than the HM group. The pattern was similar for levels of clutter and difficulties in discarding items (HD > H&H > HM). However, for the excess of acquisition, only the HM group differed significantly from the other groups (HD ≈ H&H > HM). Additionally, there was a statistically significant difference in self‐reported early experiences of material deprivation, with the H&H group scoring higher than the other groups, while no differences were found between the HD and HM groups.

### Hypothesis 1: The H&H group will score significantly higher for fear of material deprivation compared to those in the HD and HM groups

In the mixed‐model ANOVA of hoarding beliefs, there was a significant main effect of group, *F*_(2,126)_ = 19.02, *p* < .0001, *η* ^2^_G_ = .193. There was also a main effect of hoarding subscales and their interaction with group membership (H&H, HD, HM) *F*_(2,252)_ = 62.32, *p* < .0001, *η* ^2^_G_ = .093. These effects were modified by a significant group‐subscale interaction *F*_(4,252)_ = 7.74, *p* < .0001, *η* ^2^_G_ = .025, suggesting that *the pattern of differences among the subscales varied across the groups*.

Simple main effects analysis (Table 3) indicated that the H&H and HD groups exhibited higher scores for *fear of material deprivation* compared to the HM group, with no significant difference observed between themselves (H&H ≈ HD > HM). For *harm avoidance*, the H&H group displayed higher scores than the other groups, while no significant differences were found between the HD and HM groups (H&H > HD ≈ HM). Regarding the *attachment disturbance* subscale, both the H&H and HD groups scored significantly higher than the HM group, with no significant difference observed between themselves (H&H ≈ HD > HM).

**TABLE 3:** Simple main effects: means scores (SD) on each of the BAH subscales according to the group.

Subscale	HD (*n* = 43)	HM (*n* = 39)	H&H (*n* = 47)	Statistics		
	*Mean (SD)*	*Mean (SD)*	*Mean (SD)*	*F*	*df*	*p*
Fear of material deprivation	51.86^a^ (21.50)	32.22^b^ (20.63)	57.37^a^ (21.01)	16.62	2, 83	<.0001
Harm avoidance	26.70^b^ (16.11)	23.80^b^ (22.00)	46.06^a^ (23.06)	13.51	2, 80	<.0001
Attachment disturbance	49.60^a^ (24.57)	29.76^b^ (21.83)	58.28^a^ (19.88)	19.77	2, 81	<.0001

*Note*: Means with differing letters differ significantly (*p* < .05).

**FIGURE 2:**
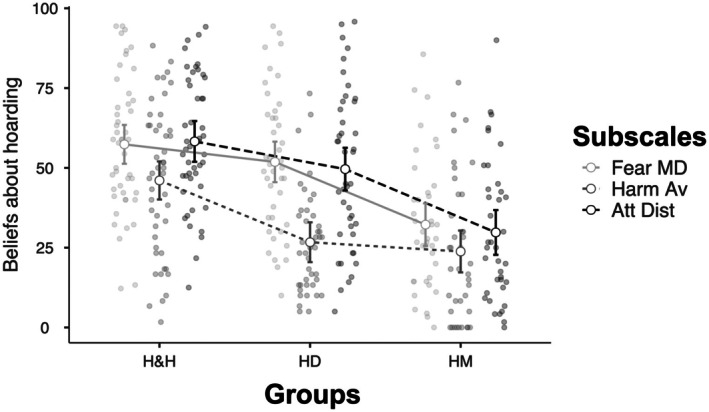
Graphic representation of the interaction between beliefs about hoarding subscales (Fear MD = Fear of material deprivation; Harm Av = Harm avoidance; Att Dist = Attachment disturbance) and study groups.

Post‐hoc comparisons using the Tukey HSD test indicated that the H&H group scored significantly higher than the HD group (*mean difference* = 11.18, *SE* = 3.998, *p* = .016) and significantly higher than the HM group (*mean difference* = 25.31, *SE* = 4.104, *p* < .0001). Additionally, the HD group scored significantly higher than the HM group (*mean difference* = 14.13, *SE* = 4.189, *p* = .003). These results suggest that the H&H group generally reported higher levels of fear of material deprivation, harm avoidance and attachment disturbance compared to the HD and HM groups (see Figure 2).

Post‐hoc comparisons using the Tukey HSD test indicated that the mean score for fear of material deprivation (*M* = 47.82, *SE* = 2.28) was significantly higher than harm avoidance (*M* = 32.86, *SE* = 2.20), *t*(126) = 9.54, *p* < .0001, but not for attachment disturbance (*M* = 45.56, *SE* = 2.44), *t*(126) = .88, *p* = .65. Harm Avoidance was also significantly lower than Attachment Disturbance, *t*(126) = −9.40, *p* < .0001.

### Hypothesis 2: The H&H will report a significantly higher number of negative experiences with housing and accommodation compared to those in the HD and HM groups

**FIGURE 3:**
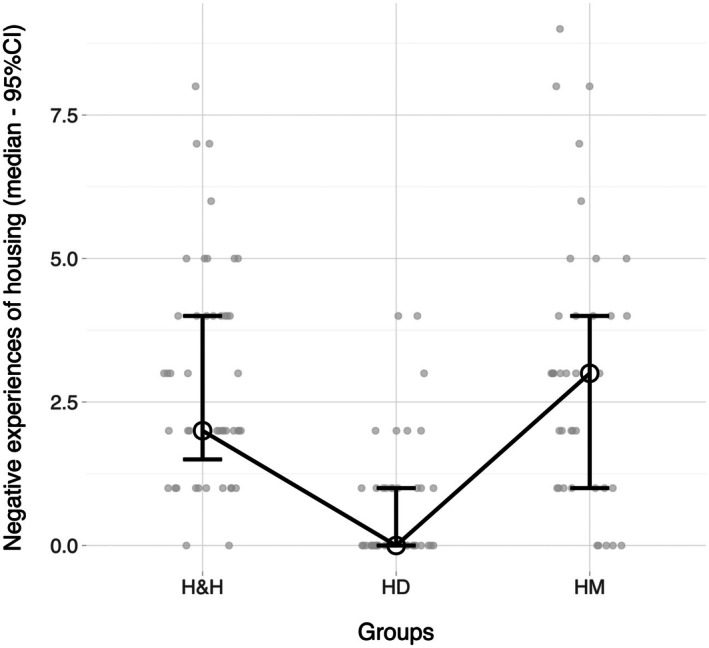
Graphic representation of self‐reported history of negative experiences with study groups.

A Kruskal‐Wallis test revealed a statistically significant difference in the number of negative experiences with housing and accommodation across the three groups, *χ* ^2^(2) = 42.188, *p* < .0001, *ε* ^2^ = .332. Pairwise comparisons using the Dwass‐Steel‐Critchlow‐Fligner procedure indicated that the H&H group reported significantly more negative housing experiences than the HD group (*W* = –8.607, *p* < .0001), but did not significantly differ from the HM group (*W* = –.031, *p* = .9997); similarly, the latter reported a significantly higher number of negative housing experiences than the HD group (*W* = 7.391, *p* < .0001) (H&H ≈ HM > HD, Figure 3).

## DISCUSSION

This study aimed to examine the intersection between hoarding and homelessness and to test the hypotheses that individuals experiencing both hoarding and homelessness would report higher fear of material deprivation than those with only hoarding or only homelessness. It was also predicted that they would report more negative housing experiences than the other two groups. Results partially confirmed our hypotheses. The H&H group reported higher levels of fear of material deprivation compared to the HM group but not the HD group. Similarly, the H&H group reported significantly more negative past housing experiences than the HD group, but no significant differences were found between the H&H and HM groups. Exploratory analysis indicated that the H&H group experienced significantly elevated levels of depressive and anxiety symptoms and greater impairment in work and social adjustment relative to other groups. The HD group had higher scores across all domains of hoarding‐related thoughts and behaviours, including overall hoarding severity, clutter and difficulty discarding. The only exception was excess of acquisition, which was similar to the H&H group and significantly higher compared to the HD group. Furthermore, the H&H group reported significantly higher levels of fear of material deprivation compared to both the HD and HM groups.

It is surprising that, despite the H&H group reporting higher levels of material hardship during childhood and adolescence, their current cognitions around fear of material deprivation do not distinguish them from the HD group. This implies that these fears may play a role in hoarding behaviours regardless of past financial struggles, suggesting that such beliefs may be intrinsic to hoarding difficulties rather than an exclusive product of deprivation history. These findings are consistent with recent research on the relationship between hoarding and experiences of material deprivation reported by Walji and Salkovskis (2024).

Notably, the only hoarding‐related domain that distinguished the H&H group from the others was harm avoidance (the belief that keeping items prevents harm to oneself or others). This aspect of hoarding may overlap with OCD (Seaman et al., 2010), a condition not assessed in this study. Pertusa et al. (2008) and Gordon et al. (2013) suggest that when hoarding co‐occurs with OCD, a common cognitive mechanism—such as perceiving threats and seeking safety—may drive both behaviours. If harm avoidance relates to seeking safety, this could partially explain its relevance to the H&H group, as accumulating possessions may provide a sense of security and control in an unpredictable environment, particularly regarding living conditions. Although this hypothesis requires empirical testing, it has been previously suggested by Cooke (2017).

In relation to the second hypothesis, past negative experiences with housing and accommodation did not distinguish the H&H group from the HM group, indicating that these groups were exposed to similar conditions in relation to their experience of homelessness. Our data may indicate that these negative experiences are more closely associated with homelessness than with hoarding and may suggest that past negative experiences with accommodation only impact hoarding behaviours when interacting with other psychological factors. Similar to early experiences of material deprivation, those negative experiences of housing and accommodation alone may not be sufficient to drive fear of material deprivation as the main function of hoarding behaviour.

Taken together, these findings suggest that the relationship between hoarding and homelessness is not driven solely by material deprivation or past housing experiences. Instead, the study points towards a more complex pattern of vulnerability. The H&H group showed markedly elevated levels of psychological distress (depression, anxiety), poorer functioning and greater exposure to adverse developmental experiences, indicating that it is the cumulative burden of social, emotional and psychological adversity, rather than deprivation alone, that distinguishes individuals who experience both hoarding difficulties and homelessness. This highlights the importance of considering *multifinality* and *cumulative risk*: homelessness and hoarding appear to converge not because of a single shared belief or experience, but because of the clustering of multiple vulnerability factors across the life course (e.g., threat sensitivity, traumatic loss, chronic instability and impaired social support). As such, the contribution of the present study lies in demonstrating that material deprivation alone is insufficient to explain this intersection and that interventions may need to target broader patterns of psychological vulnerability rather than focusing exclusively on deprivation‐related beliefs. The data also advances theoretical understanding by shifting attention away from deprivation as a sole explanatory mechanism and towards the interplay between cognitive beliefs, threat‐related processes and complex psychosocial histories.

Our findings should be considered in light of their limitations: (1) The cross‐sectional design means that findings should be interpreted with caution, and no causal relationships between the variables can be claimed. (2) Given that most participants were experiencing current homelessness, their lives can be highly unstable, leading some to respond indiscriminately and randomly, potentially introducing bias. However, we addressed this issue prior to data analysis by cross‐checking contradictory responses across different datasets (screening and completed questionnaires) and using the *responsePatterns* R algorithm (Gottfried et al., 2022) to identify patterns of indiscriminate questionnaire completion. (3) Diagnostic status for hoarding and other mental health conditions was based on self‐report and referrer judgement rather than structured clinical interviews with visual inspection of clutter. We therefore describe our sample as experiencing ‘hoarding difficulties’ rather than confirmed Hoarding Disorder, and we cannot definitively rule out alternative or comorbid explanations for hoarding‐related experiences (e.g., psychosis, substance use disorders, neurocognitive disorders). (4) We did not directly observe participants' living spaces or conduct standardized visual clutter ratings; instead, we relied on the day‐to‐day observations of collaborating staff, who identified individuals for whom clutter was sufficiently problematic to compromise safety, tenancy or service functioning. Although this is a reasonable pragmatic approach in homelessness settings, it falls short of gold‐standard multi‐method assessment. (5) In the context of homelessness and material scarcity, it is also possible that some elevation in hoarding measures, particularly clutter and saving, reflects pragmatically retaining items for survival rather than clinically maladaptive hoarding per se. Our design, while including a homelessness‐only comparison group, cannot fully disentangle adaptive saving in response to realistic deprivation from hoarding driven by dysfunctional beliefs.

A further limitation relates to the substantial drop‐off between initial expressions of interest and completed surveys. In this population, attrition primarily reflected the unpredictable and often chaotic living circumstances of individuals experiencing homelessness or hoarding difficulties (including interruptions within shared accommodation, competing demands and fluctuating emotional capacity) rather than differences in stability, organization or motivation. In addition, a proportion of cases were deliberately excluded during data‐validation procedures because of incomplete, inconsistent or patterned responding. Although this level of attrition is common in hard‐to‐engage or high‐risk populations, it may nonetheless have reduced the representativeness of the final sample. However, because attrition occurred prior to group allocation and was largely unrelated to hoarding severity or homelessness status, it is unlikely to have introduced systematic bias across groups.

Future research would benefit from combining structured diagnostic interviews (for Hoarding Disorder and major comorbidities), standardized visual clutter assessments (e.g., room‐based ratings by independent observers) and explicit measures of perceived necessity versus redundancy of possessions. Longitudinal designs that follow people through changes in housing status could help clarify how resource‐related necessity, cognitive beliefs (e.g., fear of material deprivation, harm avoidance) and observable clutter interact over time in driving or maintaining hoarding difficulties in homelessness contexts.

The findings presented here are consistent with the view that both hoarding and homelessness are complex phenomena that can result from a range of intricate life experiences, acting as psychological endpoints. It is likely that hoarding can be a pathway to homelessness, whereas the reverse (homelessness leading to hoarding) seems unlikely. However, the present study's assessment limitations mean that our conclusions should be understood as pertaining to hoarding‐related difficulties within a highly complex and comorbid homelessness population, rather than to ‘pure’ Hoarding Disorder with all differential diagnoses ruled out. Future research on the relationship between hoarding and homelessness could benefit from qualitative methods to delve deeply into the themes associated with this link. Such an approach would enhance our understanding of the psychological processes at play and facilitate the development of more nuanced and precise theoretical models.

In terms of potential clinical implications, The H&H group appears to be the most impacted by social, developmental and psychological difficulties (i.e., homelessness, early material deprivation and psychopathology), highlighting their vulnerability. Therefore, when working with this group, especially if the intervention targets hoarding difficulties, a personalized approach should be prioritized. This approach should identify all possible beliefs and functions attached to hoarding, considering harm avoidance and fear of material deprivation as potential safety‐seeking strategies. Given the complex needs of the H&H group, long‐term support with an emphasis on building trust should be considered, as brief interventions may not be appropriate and fruitful. In addition, clinicians working in homelessness services may need to distinguish, as far as possible, between possessions retained for realistic practical reasons (e.g., lack of resources, unstable housing) and possessions maintained primarily for psychological safety or driven by maladaptive beliefs, using multi‐informant and multi‐method assessment wherever feasible.

## AUTHOR CONTRIBUTIONS

**Tiago C. Zortea:** Conceptualization; investigation; writing – original draft; methodology; validation; visualization; writing – review and editing; software; formal analysis; project administration; data curation; resources. **Fiona Symington:** Project administration; resources; validation; writing – review and editing. **Alasdair Churchard:** Conceptualization; investigation; funding acquisition; resources; supervision; writing – review and editing; validation. **Paul M. Salkovskis:** Conceptualization; investigation; funding acquisition; writing – review and editing; validation; methodology; formal analysis; supervision; resources.

## CONFLICT OF INTEREST STATEMENT

The authors declare that they have no conflicts of interest.

## ETHICS STATEMENT

The authors assert that all procedures contributing to this work comply with the ethical standards of the relevant national and institutional committees on human experimentation and with the Helsinki Declaration of 1975, as revised in 2008.

## PRE‐REGISTRATION

The analysis plan for our hypotheses was pre‐registered with the University's PAS panel and at AsPredicted.org (#173686—available at https://aspredicted.org/e5cd4.pdf). All analyses beyond the hypotheses specified are exploratory.

## Supporting information


Data S1:

## Data Availability

The data that support the findings of this study are available from the corresponding author upon reasonable request.
