# Supplementary material for: Understanding hoarding behaviours in the context of homelessness
Source: Br J Clin Psychol. 2026 Mar 17;65(2):720–39. doi: 10.1111/bjc.70045 (PMC13159766; doi:10.1111/bjc.70045)
Supplement: Supplementary file 1 — Data S1: [file BJC-65-720-s001.docx]

**Supplementary Materials**

**Table of Contents**

[Appendix I: Reproducible analysis R code scripts 3](#_Toc178803862)

[R Script 1: Data preparation script 3](#_Toc178803863)

[R Script 2: Psychometric analysis script 23](#_Toc178803864)

[R Script 3: Main statistical analysis script 62](#_Toc178803865)

[Appendix II: Pre-registration of study hypotheses 100](#_Toc178803866)

[Appendix III: Blind eligibility rating of cases’ response patterns 101](#_Toc178803867)

[Appendix IV: Full descriptive statistics of the sample 112](#_Toc178803868)

##

## Appendix I: Reproducible analysis R code scripts

### R Script 1: Data preparation script

**Contents**

The current file establishes the code involved in data wrangling and preparation. This process includes:

**1. Upload of raw data files**

**2. Selection and renaming of variables of interest**

**3. Change of item responses from string text to numeric**

3.1. Socio-demographic variables
3.2. Patient Health Questionnaire-8 (PHQ-8)
3.3. Generalised Anxiety Disorder-7 (GAD-7)
3.4. Work and Social Adjustment Scale (WSAS)
3.5. The Beliefs about Hoarding Questionnaire (BAH)
3.6. The Savings Inventory-Revised (SIR)
3.7. The Interpersonal Needs Questionnaire (INQ)
3.8. Experience of Early Material Deprivation (Yes/No)
3.9. Experience of Early Material Deprivation Questionnaire (EEMDQ)
3.10. Material deprivation and hoarding beliefs (MDHB)
3.11. Experiences of Housing and Accommodation (Housing) (Yes/No)
3.12. Experiences of Homelessness (Yes/No)
3.13. Experiences of Homelessness: No Accommodation & Temporary/Crisis Accommodation
3.14. Experiences of Homelessness - Severity of Exposure

**4. Scoring of scales and subscales**

4.1. Patient Health Questionnaire-8 (PHQ-8)
4.2. Generalised Anxiety Disorder-7 (GAD-7)
4.3. Work and social adjustment scale (WSAS)
4.4. The Beliefs about Hoarding Questionnaire (BAH)
4.5. The Savings Inventory-Revised (SIR)
4.6. The Interpersonal Needs Questionnaire (INQ)
4.7. Experience of Early Material Deprivation Questionnaire (EEMDQ)
4.8. Material deprivation and hoarding beliefs (MDHB)
4.9. Experiences of Housing and Accommodation (Housing)
4.10. Experiences of Homelessness - No Accommodation
4.11. Experiences of Homelessness - Temporary/Crisis Accommodation
4.12. Experiences of Homelessness - Severity of Exposure

**5. Addition of group membership and “N” column to dataset**

**6. Identification of cases that do not match group membership and responses**

6.1. Justification for case removals
6.2. Removal of unsuitable cases from dataset

**7. Generation of datasets of separate scales for detection of potential response bias**

**8. Detection of potential response bias**

8.1. Detection of potential response bias across common scales
8.2. Detection of potential response bias for homelessness

**9. Research team’s decision-making: Removal of cases based on visual inspection of response patterns**

9.1. Researcher’s ratings
9.2. Final decision on eligibility

**10. Composition of final dataset**

**1. Upload of raw data files**

# Dependencies

library(tidyverse)

library(knitr)

library(TMB)

library(readxl)

library(readr)

library(markdown)

library(kableExtra)

library(htmltools)

library(rmarkdown)

library(summarytools)

library(haven)

library(writexl)

library(responsePatterns)

library(pacman)

library(scales)

library(gridExtra)

library(grid)

# Setting workd directory

setwd("~/Library/CloudStorage/Dropbox/DClinPsy/TDRP/Data Analysis")

# Upload datasets

data1 <- read_excel("data.sc.text.14.04.xlsx") # Screening data

data2 <- read_excel("data.sc.vouch.14.04.xlsx") # Screening voucher data

data3 <- read_excel("data.q.text.14.04.xlsx") # Screening voucher data

**2. Selection and renaming of variables of interest**

# Subset data with variables of interest and rename for data1

data1 <- data1 %>%

select(response.ID = ResponseId,

participation = `Yes/No`,

age = `SQ.1_10`,

a.Homelessness = `SQ.3.1`, # a) Homelessness

b.difficult.discarding = `SQ.3.2`, # b) Difficulty discarding

c.discarding.upsetting = `SQ.3.3`, # c) Discarding upsetting

d.save.need.future = `SQ.3.4`, # d) Save things for the future

e.items.clutter.space = `SQ.3.5`, # e) Items clutter spaces

f.accumulation.distress.life = `SQ.3.6`) # f) Accumulation distress

# Subset data with variables of interest and rename for data2

data2 <- data2 %>%

select(response.ID, age,

a.Homelessness, # a) Homelessness

b.difficult.discarding, # b) Difficulty discarding

c.discarding.upsetting, # c) Discarding upsetting

d.save.need.future, # d) Save things for the future

e.items.clutter.space, # e) Items clutter spaces

f.accumulation.distress.life, # f) Accumulation distress

eligible, # Eligibility

group, # Group

responded.questionnaire) # Whether completed questionnaire

# Remove any whitespace or invisible characters:

colnames(data3) <- gsub("\\s+", "", colnames(data3))

# Subset data with variables of interest and rename for data3

data3 <- data3 %>%

select(response.ID = `1._1`,

age = `1._4`,

gender = `2.`,

gender.same.birth = `3.`,

sexual.orient = `4.`,

ethnicity = `5.`,

live.with = `6.`,

marital.status = `7.`,

education = `8.`,

employment = `9.`,

phq.1 = `10._1`, # PHQ-8 (Depressive symptoms)

phq.2 = `10._2`,

phq.3 = `10._3`,

phq.4 = `10._4`,

phq.5 = `10._5`,

phq.6 = `10._6`,

phq.7 = `10._7`,

phq.8 = `10._8`,

gad.1 = `11_1`, # GAD-7 (Anxiety symptoms)

gad.2 = `11_2`,

gad.3 = `11_3`,

gad.4 = `11_4`,

gad.5 = `11_5`,

gad.6 = `11_6`,

gad.7 = `11_7`,

wsaj.1 = `12_1`, # Work and social adjustment scale

wsaj.2 = `12_2`,

wsaj.3 = `12_3`,

wsaj.4 = `12_4`,

wsaj.5 = `12_5`,

BAH.1 = `13_1`, # Beliefs About Hoarding Scale

BAH.2 = `13_2`,

BAH.3 = `13_3`,

BAH.4 = `13_4`,

BAH.5 = `13_5`,

BAH.6 = `13_6`,

BAH.7 = `13_7`,

BAH.8 = `13_8`,

BAH.9 = `13_9`,

BAH.10 = `13_10`,

BAH.11 = `13_11`,

BAH.12 = `13_12`,

BAH.13 = `13_13`,

BAH.14 = `13_14`,

BAH.15 = `13_15`,

BAH.16 = `13_16`,

BAH.17 = `13_17`,

BAH.18 = `13_18`,

BAH.19 = `13_19`,

BAH.20 = `13_20`,

BAH.21 = `13_21`,

BAH.22 = `13_22`,

BAH.23 = `13_23`,

BAH.24 = `13_24`,

BAH.25 = `13_25`,

BAH.26 = `13_26`,

BAH.27 = `13_27`,

BAH.28 = `13_28`,

SIR.1 = `14_1`, # The Savings Inventory-Revised

SIR.2 = `14_2`,

SIR.3 = `14_3`,

SIR.4 = `14_4`,

SIR.5 = `14_5`,

SIR.6 = `15_1`,

SIR.7 = `15_2`,

SIR.8 = `15_3`,

SIR.9 = `15_4`,

SIR.10 = `15_5`,

SIR.11 = `15_6`,

SIR.12 = `16_1`,

SIR.13 = `16_2`,

SIR.14 = `16_3`,

SIR.15 = `16_4`,

SIR.16 = `16_5`,

SIR.17 = `17_1`,

SIR.18 = `17_2`,

SIR.19 = `17_3`,

SIR.20 = `17_4`,

SIR.21 = `17_5`,

SIR.22 = `17_6`,

SIR.23 = `17_7`,

INQ.1 = `Interpersonalneeds_1`, # The Interpersonal Needs Questionnaire (INQ)

INQ.2 = `Interpersonalneeds_2`,

INQ.3 = `Interpersonalneeds_3`,

INQ.4 = `Interpersonalneeds_4`,

INQ.5 = `Interpersonalneeds_5`,

INQ.6 = `Interpersonalneeds_6`,

INQ.7 = `Interpersonalneeds_7`,

INQ.8 = `Interpersonalneeds_8`,

INQ.9 = `Interpersonalneeds_9`,

INQ.10 = `Interpersonalneeds_10`,

INQ.11 = `Interpersonalneeds_11`,

INQ.12 = `Interpersonalneeds_12`,

INQ.13 = `Interpersonalneeds_13`,

INQ.14 = `Interpersonalneeds_14`,

INQ.15 = `Interpersonalneeds_15`,

early.depriv.cat = `MatDepriv.1`, # Experience of Early Material Deprivation (Yes/No)

EEMDQ.1 = `MatDepriv.2_1`, # Experience of Early Material Deprivation Questionnaire

EEMDQ.2 = `MatDepriv.2_2`,

EEMDQ.3 = `MatDepriv.2_3`,

EEMDQ.4 = `MatDepriv.2_4`,

EEMDQ.5 = `MatDepriv.2_5`,

EEMDQ.6 = `MatDepriv.2_6`,

EEMDQ.7 = `MatDepriv.2_7`,

EEMDQ.8 = `MatDepriv.2_8`,

EEMDQ.9 = `MatDepriv.2_9`,

EEMDQ.10 = `MatDepriv.2_10`,

EEMDQ.11 = `MatDepriv.2_12`,

MDHB.1 = `Mat.Depriv.5_1`, # Material deprivation and hoarding beliefs

MDHB.2 = `Mat.Depriv.5_2`,

MDHB.3 = `Mat.Depriv.5_3`,

MDHB.4 = `Mat.Depriv.5_4`,

MDHB.5 = `Mat.Depriv.5_5`,

MDHB.6 = `Mat.Depriv.5_6`,

MDHB.7 = `Mat.Depriv.5_7`,

MDHB.8 = `Mat.Depriv.5_8`,

MDHB.9 = `Mat.Depriv.5_9`,

EHA.1 = `Housing1a`, # Experiences of Housing and Accommodation

EHA.2 = `Housing2a`,

EHA.3 = `Housing3a`,

EHA.4 = `Housing4a`,

EHA.5 = `Housing5a`,

EHA.6 = `Housing6a`,

EHA.7 = `Housing7a`,

EHA.8 = `Housing8a`,

EHA.9 = `Housing7a`,

homelessness = `SecondscreeningQue`, # Experiences of Homelessness Single Item

EHQ.NA.1 = `Homeless10a`, # Experiences of Homelessness - No Accommodation (Yes/No)

EHQ.NA.2 = `Homeless11a`,

EHQ.NA.3 = `Homeless12a`,

EHQ.NA.4 = `Homeless13a`,

EHQ.TA.1 = `Homeless15a`, # Experiences of Homelessness - Temporary/Crisis Accommodation

EHQ.TA.2 = `Homeless16a`,

EHQ.TA.3 = `Homeless17a`,

EHQ.TA.4 = `Homeless18a`,

EHQ.TA.5 = `Homeless19a`,

HOM.SEV.1 = `HomelessSeverity_1`, # Experiences of Homelessness - Severity of Exposure

HOM.SEV.2 = `HomelessSeverity_2`,

HOM.SEV.3 = `HomelessSeverity_3`,

HOM.SEV.4 = `HomelessSeverity_4`,

HOM.SEV.5 = `HomelessSeverity_5`,

HOM.SEV.6 = `HomelessSeverity_6`,

HOM.SEV.7 = `HomelessSeverity_7`,

HOM.SEV.8 = `HomelessSeverity_8`,

HOM.SEV.9 = `HomelessSeverity_9`,

HOM.SEV.10 = `HomelessSeverity_10`,

HOM.SEV.11 = `HomelessSeverity_11`,

HOM.SEV.12 = `HomelessSeverity_12`,

HOM.SEV.13 = `HomelessSeverity_13`,

HOM.SEV.14 = `HomelessSeverity_14`,

HOM.SEV.15 = `HomelessSeverity_15`,

HOM.SEV.16 = `HomelessSeverity_16`,

HOM.SEV.17 = `HomelessSeverity_17`,

HOM.SEV.18 = `HomelessSeverity_18`,

HOM.SEV.19 = `HomelessSeverity_19`,

HOM.SEV.20 = `HomelessSeverity_20`,

HOM.SEV.21 = `HomelessSeverity_21`,

HOM.SEV.22 = `HomelessSeverity_22`,

HOM.SEV.23 = `HomelessSeverity_23`,

HOM.SEV.24 = `HomelessSeverity_24`,

HOM.SEV.25 = `HomelessSeverity_25`,

HOM.SEV.26 = `HomelessSeverity_26`,

HOM.SEV.27 = `HomelessSeverity_27`,

HOM.SEV.28 = `HomelessSeverity_28`,

HOM.SEV.29 = `HomelessSeverity_29`,

HOM.SEV.30 = `HomelessSeverity_30`,

HOM.SEV.31 = `HomelessSeverity_31`,

HOM.SEV.32 = `HomelessSeverity_32`,

HOM.SEV.33 = `HomelessSeverity_33`,

HOM.SEV.34 = `HomelessSeverity_34`,

HOM.SEV.35 = `HomelessSeverity_35`,

HOM.SEV.36 = `HomelessSeverity_36`)

# Remove line 2 (variable subtitle)

data1 <- data1[-1, ]

data3 <- data3[-1, ]

# Remove testing cases and leaving only participant cases

data3 <- data3 %>%

slice(23:n())

# Filter out rows where response.ID is NA (participants who did not complete)

data3 <- data3 %>%

filter(!is.na(response.ID),

!is.na(phq.1))

# Parallel processing options are set appropriately for RStudio

# running on macOS with R version 4.0.0 or higher

if (Sys.getenv("RSTUDIO") == "1" && !nzchar(Sys.getenv("RSTUDIO_TERM")) &&

Sys.info()["sysname"] == "Darwin" && getRversion() >= "4.0.0") {

parallel:::setDefaultClusterOptions(setup_strategy = "sequential")}

**3. Change of item responses from string text to numeric**

*3.1. Socio-demographic variables*

data3 <- data3 %>%

mutate(response.ID = as.character(response.ID), # Convert response.ID to string

age = as.numeric(age), # Convert age to numeric

gender = factor( # Convert gender to factor with modified levels

gender,

levels = c("Female", "Male", "Non-binary / another gender"),

labels = c("Female", "Male", "Non-binary")),

sexual.orient = factor( # Convert sexual.orient to factor with modified levels

sexual.orient,

levels = c(

"Straight / Heterosexual",

"Gay / Homosexual",

"Bisexual",

"Asexual",

"Pansexual",

"Lesbian",

"Prefer not to say"),

labels = c("Heterosexual",

"Homosexual",

"Bisexual",

"Asexual",

"Pansexual",

"Homosexual",

"Prefer not to say")),

ethnicity = factor(ethnicity), # Convert ethnicity to factor

live.with = factor(live.with), # Convert live.with to factor

marital.status = factor(marital.status), # Convert marital.status to factor

education = factor(education), # Convert education to factor

employment = factor(employment) # Convert employment to factor

)

*3.2. Patient Health Questionnaire-8 (PHQ-8)*

# Define a function to convert text levels to numbers

convert_text_to_numbers <- function(x) {

factor(x,

levels = c("Not at all", "Several days",

"More than half the days", "Nearly every day"),

labels = c(0, 1, 2, 3),

ordered = TRUE)

}

# Apply the function to each PHQ variable and convert to numeric

data3 <- data3 %>%

mutate(across(starts_with("phq."), ~ as.numeric(as.character(convert_text_to_numbers(.)))))

*3.3. Generalised Anxiety Disorder-7 (GAD-7)*

# Apply the same function to the GAD variables and convert to numeric

data3 <- data3 %>%

mutate(across(starts_with("gad."), ~ as.numeric(as.character(convert_text_to_numbers(.)))))

*3.4. Work and social adjustment scale (WSAS)*

# Convert Work and social adjustment scale to numeric

data3 <- data3 %>%

mutate(across(starts_with("wsaj."), as.numeric))

*3.5. The Beliefs about Hoarding Questionnaire (BAH)*

# Convert The Beliefs about Hoarding Questionnaire to numeric

data3 <- data3 %>%

mutate(across(starts_with("BAH."), as.numeric))

*3.6. The Savings Inventory-Revised*

# Convert The Savings Inventory-Revised to numeric

# Define a function to recode levels and convert to numeric

recode_and_convert <- function(data, columns, levels) {

data <- data %>%

mutate_at(vars(all_of(columns)), ~recode(., !!!levels)) %>%

mutate(across(all_of(columns), as.numeric))

return(data)

}

# Recode and convert variables SIR.1 to SIR.5

data3 <- recode_and_convert(data3, c("SIR.1", "SIR.2", "SIR.3", "SIR.4", "SIR.5"),

list("None" = 0,

"A little" = 1,

"A moderate amount" = 2,

"Most / Much" = 3,

"Almost all / complete" = 4))

# Recode and convert variables SIR.6 to SIR.16

data3 <- recode_and_convert(data3, paste0("SIR.", 6:16),

list("Not at all" = 0,

"Mild" = 1,

"Moderate" = 2,

"Considerable / severe" = 3,

"Extreme" = 4))

# Recode and convert variables SIR.17 to SIR.23

data3 <- recode_and_convert(data3, paste0("SIR.", 17:23),

list("Never" = 0,

"Rarely" = 1,

"Sometimes / Occasionally" = 2,

"Frequently / Often" = 3,

"Very Often" = 4))

*3.7. The Interpersonal Needs Questionnaire (INQ)*

# Define a function to recode levels and convert to numeric

recode_and_convert <- function(data, columns, levels) {

data <- data %>%

mutate_at(vars(all_of(columns)), ~recode(., !!!levels)) %>%

mutate(across(all_of(columns), as.numeric))

return(data)}

# Recode and convert variables INQ.1 to INQ.12

data3 <- recode_and_convert(data3, c("INQ.1",

"INQ.2",

"INQ.3",

"INQ.4",

"INQ.5",

"INQ.6",

"INQ.9",

"INQ.11",

"INQ.12"),

list("1 = Not at all true for me" = 1,

"2" = 2,

"3" = 3,

"4 = Somewhat true for me" = 4,

"5" = 5,

"6" = 6,

"7 = Very true for me" = 7))

# Recode, convert, and reverse variables INQ.7, INQ.8, INQ.10, INQ.13, INQ.14, INQ.15

data3 <- recode_and_convert(data3, c("INQ.7",

"INQ.8",

"INQ.10",

"INQ.13",

"INQ.14",

"INQ.15"),

list("1 = Not at all true for me" = 7,

"2" = 6,

"3" = 5,

"4 = Somewhat true for me" = 4,

"5" = 3,

"6" = 2,

"7 = Very true for me" = 1))

*3.8. Experience of Early Material Deprivation (Yes/No)*

# Convert Experience of Early Material Deprivation (Yes/No) to factor

data3$early.depriv.cat <- as.factor(data3$early.depriv.cat)

*3.9. Experience of Early Material Deprivation Questionnaire (EEMDQ)*

# Recode and convert specified variables to numeric

data3 <- data3 %>%

mutate(across(.cols = starts_with("EEMDQ."),

.fns = ~case_when(

. == "never struggled to obtain" ~ 0,

. == "rarely struggled to obtain" ~ 1,

. == "sometimes struggled to obtain" ~ 2,

. == "often struggled to obtain" ~ 3,

. == "always struggled to obtain" ~ 4,

TRUE ~ NA_real_ # for any unexpected cases

))) %>%

mutate(across(.cols = starts_with("EEMDQ."), as.numeric))

*3.10. Material deprivation and hoarding beliefs (MDHB)*

# Apply changes to specific variables and convert to numeric

data3 <- data3 %>%

mutate(across(.cols = starts_with("MDHB."),

.fns = ~case_when(

. == "0 = no influence at all" ~ 0,

. == "1 = a slight influence" ~ 1,

. == "2 = a moderate influence" ~ 2,

. == "3 = a strong influence" ~ 3,

TRUE ~ as.numeric(.) # handles existing numeric values and converts NAs where needed

)))

*3.11. Experiences of Housing and Accommodation (Housing) (Yes/No)*

# Apply changes to specific variables and convert to numeric

data3 <- data3 %>%

mutate(across(.cols = starts_with("EHA."),

.fns = ~case_when(

. == "No" ~ 0,

. == "Yes" ~ 1,

TRUE ~ as.numeric(.) # handles existing numeric values and converts NAs where needed

)))

*3.12. Experiences of Homelessness (Yes/No)*

# Apply changes to specific variables and convert to factor

data3$homelessness <- as.factor(data3$homelessness)

*3.13. Experiences of Homelessness: No Accommodation & Temporary/Crisis Accommodation*

# Recode specified variables from "Yes"/"No" to 1/0 and convert to numeric

data3 <- data3 %>%

mutate(across(.cols = c(EHQ.NA.1,

EHQ.NA.2,

EHQ.NA.3,

EHQ.NA.4,

EHQ.TA.1,

EHQ.TA.2,

EHQ.TA.3,

EHQ.TA.4,

EHQ.TA.5),

.fns = ~case_when(

. == "Yes" ~ 1,

. == "No" ~ 0,

TRUE ~ as.numeric(.) # handles converting any unexpected values to NA

)))

*3.14. Experiences of Homelessness - Severity of Exposure (HOM.SEV)*

# Recode specified variables from textual descriptions to numbers and convert to numeric

data3 <- data3 %>%

mutate(across(.cols = starts_with("HOM.SEV."),

.fns = ~case_when(

. == "Does not describe me" ~ 0,

. == "Describes me slightly well" ~ 1,

. == "Describes me moderately well" ~ 2,

. == "Describes me very well" ~ 3,

. == "Describes me extremely well" ~ 4,

TRUE ~ as.numeric(.) # handles converting any unexpected values to NA

)))

**4. Scoring of scales and subscales**

*4.1. Patient Health Questionnaire-8 (PHQ-8)*

# Assuming data3 is your dataset

data3 <- data3 %>%

mutate(phq.total = phq.1 + phq.2 + phq.3 + phq.4 + phq.5 + phq.6 + phq.7 + phq.8)

*4.2. Generalised Anxiety Disorder-7 (GAD-7)*

# Assuming data3 is your dataset

data3 <- data3 %>%

mutate(gad.total = gad.1 + gad.2 + gad.3 + gad.4 + gad.5 + gad.6 + gad.7)

*4.3. Work and social adjustment scale (WSAS)*

# Assuming data3 is your dataset

data3 <- data3 %>%

mutate(wsas.total = wsaj.1 + wsaj.2 + wsaj.3 + wsaj.4 + wsaj.5)

# Scoring instructions: https://t.ly/hK-cl

*4.4. The Beliefs about Hoarding Questionnaire (BAH)*

# BAH Harm Avoidance

data3 <- data3 %>%

mutate(bah.harm.avoi = round((BAH.6 + BAH.12 + BAH.21 + BAH.24 + BAH.25 + BAH.28) / 6, 1))

# BAH Fear of Material Deprivation

data3 <- data3 %>%

mutate(bah.fear.mat.depr = round((BAH.1 + BAH.5 + BAH.7 + BAH.10 + BAH.13 + BAH.15 + BAH.18 + BAH.23 + BAH.27) / 9, 1))

# BAH Attachment Disturbance

data3 <- data3 %>%

mutate(bah.att.dist = round((BAH.2 + BAH.3 + BAH.4 +

BAH.8 + BAH.11 + BAH.14 +

BAH.16 + BAH.17 + BAH.19 +

BAH.20 + BAH.22 + BAH.27) / 12, 1))

# Scoring instructions: http://dx.doi.org/10.1016/j.janxdis.2013.02.009

#| Harm avoidance subscale: Items 6, 12, 21, 24, 25, 28.

#| Fear of material deprivation subscale: Items 1, 5, 7, 10, 13, 15, 18, 23, 27.

#| Attachment disturbance subscale: Items 2, 3, 4, 8, 11, 14, 16, 17, 19, 20, 22, 26.

#| Positive emotion associated with acquiring: Item 9.

*4.5. The Savings Inventory-Revised (SIR)*

# Total SIR:

data3 <- data3 %>%

mutate(

SIR.2 = 4 - SIR.2, # Reverse values for SIR.2

SIR.4 = 4 - SIR.4, # Reverse values for SIR.4

sir.total = SIR.1 + SIR.2 + SIR.3 + SIR.4 + SIR.5 + SIR.6 + SIR.7 + SIR.8 +

SIR.9 + SIR.10 + SIR.11 + SIR.12 + SIR.13 + SIR.14 + SIR.15 +

SIR.16 + SIR.17 + SIR.18 + SIR.19 + SIR.20 + SIR.21 + SIR.22 + SIR.23)

# Clutter scale:

data3 <- data3 %>%

mutate(sir.clutter = SIR.1 + SIR.3 + SIR.5 + SIR.8 +

SIR.10 + SIR.12 + SIR.15 + SIR.20 + SIR.22)

# Difficulty Discarding scale:

data3 <- data3 %>%

mutate(sir.diffic.discard = SIR.4 + SIR.6 + SIR.7 +

SIR.13 + SIR.17 + SIR.19 + SIR.23)

# Excessive Acquisition scale:

data3 <- data3 %>%

mutate(sir.exces.acquis = SIR.2 + SIR.9 + SIR.11 +

SIR.14 + SIR.16 + SIR.18 + SIR.21)

# Scoring instructions: https://academic.oup.com/book/1180/chapter/138282210?login=false

#| Scoring the SI-R:

#| - SI-R total, reverse score items 2 and 4 and sum with the remaining 21 items.

#| - Clutter scale, sum items 1, 3, 5, 8, 10, 12, 15, 20, and 22.

#| - Difficulty Discarding scale, reverse score item 4 and add 6, 7, 13, 17, 19, & 23.

#| - Excessive Acquisition scale, reverse score item 2 and add 9, 11, 14, 16, 18, & 21.

*4.6. The Interpersonal Needs Questionnaire (INQ)*

# Perceived Burdensomeness

data3 <- data3 %>%

mutate(inq.pb = round((INQ.1 + INQ.2 + INQ.3 + INQ.4 + INQ.5 + INQ.6) / 6, 1))

# Thwarted Belongingness

data3 <- data3 %>%

mutate(inq.tb = round((INQ.7 + INQ.8 + INQ.9 + INQ.10 + INQ.11 + INQ.12 + INQ.13 + INQ.14 + INQ.15) / 9, 1))

# Scoring instructions: https://psy.fsu.edu/~joinerlab/measures/INQ-15.pdf

#| Scoring the INQ:

#| Perceived Burdensomeness (inq.pb): Average of items 1 to 6.

#| Thwarted Belongingness (inq.tb): Averave of items 7 to 15.

*4.7. Experience of Early Material Deprivation Questionnaire (EEMDQ)*

# Scoring the EEMDQ

data3 <- data3 %>%

mutate(eemdq.total = EEMDQ.1 + EEMDQ.2 + EEMDQ.3 + EEMDQ.4 + EEMDQ.5 +

EEMDQ.6 + EEMDQ.7 + EEMDQ.8 + EEMDQ.9 + EEMDQ.10 + EEMDQ.11)

*4.8. Material deprivation and hoarding beliefs (MDHB)*

# Scoring the MDHB

data3 <- data3 %>%

mutate(mdhb.total = MDHB.1 + MDHB.2 + MDHB.3 + MDHB.4 +

MDHB.5 + MDHB.6 + MDHB.7 + MDHB.8 + MDHB.9)

*4.9. Experiences of Housing and Accommodation (Housing)*

# Scoring the Experiences of Housing and Accommodation

data3 <- data3 %>%

mutate(housing.total = EHA.1 + EHA.2 + EHA.3 + EHA.4 +

EHA.5 + EHA.6 + EHA.7 + EHA.8 + EHA.9)

*4.10. Experiences of Homelessness - No Accommodation*

# Scoring the Experiences of Housing and Accommodation

data3 <- data3 %>%

mutate(no.accom = EHQ.NA.1 + EHQ.NA.2 + EHQ.NA.3 + EHQ.NA.4)

*4.11. Experiences of Homelessness - Temporary/Crisis Accommodation*

# Scoring Experiences of Homelessness - Temporary/Crisis Accommodation

data3 <- data3 %>%

mutate(temp.accom = EHQ.TA.1 + EHQ.TA.2 + EHQ.TA.3 + EHQ.TA.4 + EHQ.TA.5)

*4.12. Experiences of Homelessness - Severity of Exposure*

# Experiences of Homelessness - Severity of Exposure

data3 <- data3 %>%

mutate(hom.sev.total = rowMeans(select(., starts_with("HOM.SEV.")), na.rm = TRUE)) %>%

mutate(hom.sev.total = ifelse(is.nan(hom.sev.total), NA, round(hom.sev.total, 1)))

**5. Addition of group membership and “N” column to dataset**

# Merging 'group' from data2 into data3 based on 'response.ID'

data3 <- data3 %>%

left_join(data2 %>% select(response.ID, group, a.Homelessness, b.difficult.discarding, c.discarding.upsetting, d.save.need.future, e.items.clutter.space, f.accumulation.distress.life, eligible), by = "response.ID")

# Specifying 'group' as factor

data3$group <- as.factor(data3$group)

# Add a column 'N' with row number and make it the first column in the dataset

data3 <- data3 %>%

mutate(N = row_number()) %>%

relocate(N, .before = 1) # Move 'N' to be the first column

**6. Identification of cases that do not match group membership and responses**

# Filter cases where 'group' is NA and select 'response.ID'

na_group_ids <- data3 %>%

filter(is.na(group)) %>%

select(response.ID)

# View the IDs

print(na_group_ids)

#| PROCEDURES TO CORRECT MATCHING:

#| 1. Identify the IDs listed below in the data.q.text.14.04 Excel File.

#| 2. Identify name of participant.

#| 3. Search participant's name on the data.sc.vouch.14.04 Excel File.

#| 4. Copy the ID code from data.sc.vouch.14.04.

#| 5. Replace the ID code in the data.q.text.14.04 Excel File.

# Identification of cases that don't match group membership and responses

id.group.checks <- data3 %>%

select(N, response.ID, group, eligible, homelessness, HOM.SEV.36, hom.sev.total,

a.Homelessness, b.difficult.discarding, c.discarding.upsetting,

d.save.need.future, e.items.clutter.space, f.accumulation.distress.life)

# Save data3 in SPSS format

write_sav(data3, "data3.sav")

# Save data3 in Excel format

write_xlsx(data3, "data3.xlsx")

# Save id.group.checks in Excel format

write_xlsx(id.group.checks, "id_group_checks.xlsx")

# Selection of cases that don't match group membership and responses

selected_rows <- c(32, 34, 46, 77, 80, 87, 98, 135) # Define the row numbers to be selected

inconsistencies <- data3[data3$N %in% selected_rows, ] # Select rows with the specified row numbers

kable(inconsistencies[, c("N", "response.ID", "group")]) %>%

kable_styling(full_width = FALSE) # View the 'inconsistencies' dataset

| **N** | **response.ID** | **group** |
| --- | --- | --- |
| 32 | R_32MhErVwg0eHz6m | H&H |
| 34 | R_tMc36yFHhExKXdf | HD |
| 46 | R_32MhErVwg0eHz6m | H&H |
| 77 | R_3qP5imd9onxn71U | H&H |
| 80 | R_5flV7yxgoPrXQDm | H&H |
| 87 | R_2jy1w3tWir7jMdQ | HM |
| 98 | R_7mm1g5SHaXHpPOC | H&H |
| 135 | R_2Xo3SrvaKHk85hL | H&H |

*6.1. Justification for case removals*

After careful analysis of each case with missing data and discussion with the research group, the following participants were removed from the final dataset:

- **Participant 32 (H&H)**: Reported that they experienced homelessness in the screening questionnaire. However, when the same question was asked in the study questionnaire, they said they had never experienced homelessness and did not complete any measure of experiences of homelessness. Keep in mind this is the same question, but asked in different stages (screening, then study questionnaire).
- **Participant 34 (HD)**: Opposite of Participant 32 – Never experienced homelessness in the screening questionnaire, but reported experiencing it in the main study questionnaire.
- **Participant 46 (H&H)**: Same as Participant 32.
- **Participant 77 (H&H)**: The same participant completed twice (online) – although records indicate that only one voucher was sent. This could have happened because of computer problems during survey completion.
- **Participant 68 (HD)**: Did not complete the questionnaire and was not sent the voucher.
- **Participants 80 (H&H) and 87 (HM)**: Same issue as Participant 32.
- **Participant 98 (H&H)**: Did not complete the questionnaire and was not sent the voucher.
- **Participant 135 (H&H)**: Did not complete the questionnaire and was not sent the voucher.

*6.2. Removal of unsuitable cases from dataset*

# Define the row numbers to be removed

rows_to_remove <- c(32, 34, 46, 77, 80, 87, 98, 135)

# Remove rows with the specified row numbers

data3 <- data3 %>%

filter(!N %in% rows_to_remove)

**7. Generation of datasets of separate scales for detection of potential response bias**

# PHQ and GAD

phq.gad.scales <- data3 %>%

select(N,

phq.1:phq.8,

gad.1:gad.7)

write_csv(phq.gad.scales, "phq.gad.scales.csv") # Saving PHQ and GAD

# WSAJ

wsaj.scale <- data3 %>%

select(N,

wsaj.1:wsaj.5)

write_csv(wsaj.scale, "wsaj.scale.csv") # Saving WSAJ

# BAH Scale

bah.scale <- data3 %>%

select(N,

BAH.1:BAH.28)

write_csv(bah.scale, "bah.scale.csv") # Saving BAH

# SIR Scales

sir.scale <- data3 %>%

select(N,

SIR.1:SIR.23)

write_csv(sir.scale, "sir.scale.csv") # Saving SIR

# INQ Scales

inq.scale <- data3 %>%

select(N,

INQ.1:INQ.15)

write_csv(inq.scale, "inq.scale.csv") # Saving INQ

# EEMDQ Scales

eemq.scale <- data3 %>%

select(N,

EEMDQ.1:EEMDQ.11)

write_csv(eemq.scale, "eemq.scale.csv") # Saving EEMDQ

# MDHB Scales

mdhb.scale <- data3 %>%

select(N,

MDHB.1:MDHB.9)

write_csv(mdhb.scale, "mdhb.scale.csv") # Saving MDHB

# HOM.SEV Scales

hom.sev.scale <- data3 %>%

select(N, HOM.SEV.1:HOM.SEV.36) %>%

na.omit()

**8. Detection of potential response bias**

To detect response bias across different participants (e.g., participants who responded to all items of a scale with the same values throughout the whole questionnaire) we employed Autocorrelation Screening via a ShinyApp that utilises the *responsePatterns* R package, which screens for careless responding patterns. The ShinyApp is availale online (URL provided below).

<https://jargottfried.shinyapps.io/Autocorrelation_screening/>

*8.1. Detection of potential response bias across common scales*

# Generate different dataset (due to NAs in the homelessness questionnaires)

data4 <- data3 %>%

select(N,

phq.1:phq.8, # PHQ

gad.1:gad.7, # GAD

wsaj.1:wsaj.5, # Work and social adjustment

BAH.1:BAH.28, # Beliefs about hoarding

SIR.1:SIR.23, # Savings nventory revised

INQ.1:INQ.15, # Interpersonal needs

EEMDQ.1:EEMDQ.11, # Early experiences of material deprivation questionnaire

MDHB.1:MDHB.9) %>% # Material deprivation and hoarding

mutate_at(vars(-N), as.numeric)

# Rescale (standardise data 4) all numeric columns except "N" to the range 0 to 100 to identify patterns of answers

rescaled_data4 <- data4 %>%

mutate(across(-N, ~ rescale(scale(.), to = c(0, 100)))) %>% # Rescale data

mutate(across(everything(), as.numeric)) # Ensure all data is numeric

# Create a Plot function to generate plots of patterns of responses

plot.pattern <- function(data, id) {

# Filter data for the specific case ID

case_data <- data[data$N == id, ]

# Exclude the "N" variable from the range

variable_range <- setdiff(1:ncol(case_data), which(names(case_data) == "N"))

# Define the ranges for each scale

scale_ranges <- list(

PHQ = 2:9,

GAD = 10:16,

WSAJ = 17:21,

BAH = 22:49,

SIR = 50:72,

INQ = 73:87,

EEMDQ = 88:98,

MDHB = 99:107,

HOM_SEV = 108:143

)

# Create a data frame to store the data for plotting

plot_data <- data.frame(

scale = rep(names(scale_ranges), each = length(variable_range)),

variable = rep(variable_range, times = length(scale_ranges)),

value = as.vector(t(case_data[, variable_range])) # Exclude "N" variable

)

# Plot

p <- ggplot(plot_data, aes(x = variable, y = value, group = scale)) +

geom_line(size = 0.4) + # Adjust thickness of the line

geom_point(size = 2.0) + # Adjust size of the points

scale_x_continuous(

breaks = c(2, 10, 17, 22, 50, 73, 88, 99, 108), # Set breaks to the start of each scale

labels = names(scale_ranges)

) +

labs(

title = paste("Patterns of response for case", id),

x = "Scales",

y = "Values"

) +

theme(axis.text.x = element_text(angle = 45, hjust = 1), # Rotate x-axis labels

axis.title.y = element_text(size = 15), # Increase y-axis label size

plot.title = element_text(size = 14), # Increase title size

plot.margin = margin(1, 1, 1, 1, "cm")) # Adjust plot margins

return(p)

}

p.pattern.1 <- plot.pattern(rescaled_data4, id = 76) # BAH, HOM.SEV, INQ, WSAJ

p.pattern.2 <- plot.pattern(rescaled_data4, id = 86) # BAH, HOM.SEV, WSAJ

p.pattern.3 <- plot.pattern(rescaled_data4, id = 120) # BAH, HOM.SEV, INQ, PHQ, GAD

p.pattern.4 <- plot.pattern(rescaled_data4, id = 122) # BAH, PHQ, GAD, SIR

p.pattern.5 <- plot.pattern(rescaled_data4, id = 140) # BAH

p.pattern.6 <- plot.pattern(rescaled_data4, id = 146) # BAH, SIR

p.pattern.7 <- plot.pattern(rescaled_data4, id = 42) # HOM.SEV

p.pattern.8 <- plot.pattern(rescaled_data4, id = 40) # INQ, WSAJ

p.pattern.9 <- plot.pattern(rescaled_data4, id = 68) # INQ, WSAJ

p.pattern.10 <- plot.pattern(rescaled_data4, id = 82) # INQ

p.pattern.11 <- plot.pattern(rescaled_data4, id = 83) # INQ

p.pattern.12 <- plot.pattern(rescaled_data4, id = 11) # MDHB

p.pattern.13 <- plot.pattern(rescaled_data4, id = 23) # MDHB

p.pattern.14 <- plot.pattern(rescaled_data4, id = 25) # MDHB

p.pattern.15 <- plot.pattern(rescaled_data4, id = 27) # MDHB

p.pattern.16 <- plot.pattern(rescaled_data4, id = 12) # PHQ, GAD

p.pattern.17 <- plot.pattern(rescaled_data4, id = 17) # PHQ, GAD, WSAJ

p.pattern.18 <- plot.pattern(rescaled_data4, id = 22) # PHQ, GAD

p.pattern.19 <- plot.pattern(rescaled_data4, id = 113) # PHQ, GAD

p.pattern.20 <- plot.pattern(rescaled_data4, id = 108) # SIR

p.pattern.21 <- plot.pattern(rescaled_data4, id = 115) # SIR

p.pattern.22 <- plot.pattern(rescaled_data4, id = 58) # SIR

p.pattern.23 <- plot.pattern(rescaled_data4, id = 26) # SIR

p.pattern.24 <- plot.pattern(rescaled_data4, id = 44) # WSAJ

p.pattern.25 <- plot.pattern(rescaled_data4, id = 106) # WSAJ

########| ALL PLOTS TOGETHER |######## OPTIONAL

# grid.arrange(p.pattern.1, p.pattern.2, p.pattern.3, p.pattern.4, p.pattern.5, p.pattern.6, p.pattern.7, p.pattern.8, p.pattern.9, p.pattern.10, p.pattern.11, p.pattern.12, p.pattern.13, p.pattern.14, p.pattern.15, p.pattern.16, p.pattern.17, p.pattern.18, p.pattern.19, p.pattern.20, p.pattern.21, p.pattern.22, p.pattern.23, p.pattern.24, p.pattern.25, ncol=2)

p.pattern.1

Plots generated and pasted into the rating questionnaire for researchers.

*8.2. Detection of potential response bias for homelessness*

# Selecting cases that experienced homelessness

data5 <- data3 %>%

select(N,

phq.1:phq.8, # PHQ Scale

gad.1:gad.7, # GAD Scale

wsaj.1:wsaj.5, # Work and social adjustment Scale

BAH.1:BAH.28, # Beliefs about hoarding Scale

SIR.1:SIR.23, # Savings Inventory revised scale

INQ.1:INQ.15, # Interpersonal needs scale

EEMDQ.1:EEMDQ.11, # Early experiences of material deprivation questionnaire

MDHB.1:MDHB.9, # Material deprivation and hoarding scale

HOM.SEV.1:HOM.SEV.36) %>% # Severity of exposure to homelessness scale

mutate_at(vars(-N), as.numeric) %>%

drop_na()

# Rescale (standardise data 5) all numeric columns except "N" to the range 0 to 100 to identify patterns of answers

rescaled_data5 <- data5 %>%

mutate(across(-N, ~ rescale(scale(.), to = c(0, 100)))) %>% # Rescale data

mutate(across(everything(), as.numeric)) # Ensure all data is numeric

p.pattern.1b <- plot.pattern(rescaled_data5, id = 76) # BAH, HOM.SEV, INQ, WSAJ

p.pattern.2b <- plot.pattern(rescaled_data5, id = 86) # BAH, HOM.SEV, WSAJ

p.pattern.3b <- plot.pattern(rescaled_data5, id = 120) # BAH, HOM.SEV, INQ, PHQ, GAD

p.pattern.4b <- plot.pattern(rescaled_data5, id = 122) # BAH, PHQ, GAD, SIR

p.pattern.5b <- plot.pattern(rescaled_data5, id = 140) # BAH

p.pattern.6b <- plot.pattern(rescaled_data5, id = 146) # BAH, SIR

p.pattern.7b <- plot.pattern(rescaled_data5, id = 42) # HOM.SEV

p.pattern.8b <- plot.pattern(rescaled_data5, id = 40) # INQ, WSAJ

p.pattern.9b <- plot.pattern(rescaled_data5, id = 82) # INQ

p.pattern.10b <- plot.pattern(rescaled_data5, id = 25) # MDHB

p.pattern.11b <- plot.pattern(rescaled_data5, id = 12) # PHQ, GAD

p.pattern.12b <- plot.pattern(rescaled_data5, id = 108) # SIR

p.pattern.13b <- plot.pattern(rescaled_data5, id = 115) # SIR

p.pattern.14b <- plot.pattern(rescaled_data5, id = 58) # SIR

p.pattern.15b <- plot.pattern(rescaled_data5, id = 44) # WSAJ

p.pattern.16b <- plot.pattern(rescaled_data5, id = 106) # WSAJ

########| ALL PLOTS TOGETHER |######## OPTIONAL

# grid.arrange(p.pattern.1b, p.pattern.2b, p.pattern.3b, p.pattern.4b, p.pattern.5b, p.pattern.6b, p.pattern.7b, p.pattern.8b, p.pattern.9b, p.pattern.10b, p.pattern.11b, p.pattern.12b, p.pattern.13b, p.pattern.14b, p.pattern.15b, p.pattern.16b, ncol=2)

p.pattern.1b

**9. Research team’s decision-making: Removal of cases based on visual inspection of response patterns**

Upon identification of such cases with potential response bias and the generation of graphical plots illustrating the response patterns of each potentially unreliable case, these plots were then presented to the research team (TZ, PS, FS, AC) for visual inspection and subsequent decision-making regarding the inclusion or exclusion of the identified cases from the dataset.

To mitigate bias in decision-making and ensure robustness and reliability, all members of the research team participated blindly in the evaluation process. Each plot depicted responses on a Likert-type scale standardized from 0 to 100, with the y-axis representing the scale and the x-axis indicating the range of each scale.

The research team was instructed to evaluate each plot based on the variation of responses across items and scales, with a particular emphasis on identifying an excess of response repetition indicative of unreliable responding. It was noted that certain scales, such as HOM_SEV, EEMDQ, and MDHB, might exhibit repeated responses due to the broad definition of homelessness and the inclusion of items related to the effects of rough sleeping. Therefore, the decision-making process required consideration of the entire plot and the underlying patterns of responses to ensure accurate judgement. An external referee (SA) was invited to contribute to this evaluation process to resolve existing disagreements.

Below we present the final decision on inclusion/exclusion of cases, following the decision-making process.

*9.1. Researcher’s ratings*

The plots below represent the decisions made by each researcher (AC, FS, PS, SA, and TZ) on whether including or excluding cases from the final dataset. The colours indicate those decisions, and the scale at the bottom represents the researchers’ self-reported level of confidence in their decision (ranging from 0 = Not confident at all to 10 = Extremely confident).

# Upload researchers decisions data

data6 <- read_excel("data.visual.inspection.xlsx") # Screening data

# 1. Remove the variable Timestamp

data6 <- data6 %>%

select(-Timestamp)

# 2. Change the name of the variable Your name:

data6 <- data6 %>%

rename(Researcher = `Your name:`) %>%

mutate(Researcher = factor(case_when(

Researcher == "Tiago" ~ "TZ",

Researcher == "Paul Salkovskis" ~ "PS",

Researcher == "Alasdair" ~ "AC",

Researcher == "Fiona" ~ "FS",

Researcher == "Saarim Aslam" ~ "SA"

)))

# 3. Change values in the variable Case 11 and Case 12

data6 <- data6 %>%

mutate(`Case 11` = factor(case_when(

`Case 11` == "Include in the dataset" ~ "Include",

TRUE ~ `Case 11`

)),

`Case 12` = factor(case_when(

`Case 12` == "Include in the dataset" ~ "Include",

TRUE ~ `Case 12`

)))

# 4. Change names and values of variables

data6 <- data6 %>%

rename_with(~ gsub("\\?", "", paste0("confidence.", gsub("How confident do you feel about your decision regarding case ", "", .))),

starts_with("How confident")) %>%

mutate(across(starts_with("confidence"), as.numeric))

# 5. Change variable types for Case X

data6 <- data6 %>%

mutate(across(starts_with("Case"), as.factor))

# Plotting of decision making

# Separating 'Case' columns and 'confidence' columns

case_data <- data6 %>% select(Researcher, starts_with("Case"))

confidence_data <- data6 %>% select(Researcher, starts_with("confidence"))

# Pivoting each dataset to long format

case_long <- pivot_longer(case_data, -Researcher, names_to = "Case", values_to = "Decision")

confidence_long <- pivot_longer(confidence_data, -Researcher, names_to = "Confidence_Case", values_to = "Confidence")

# Removing the 'Case' and 'confidence.' prefix to match the cases and confidence columns

case_long$Case <- sub("Case ", "", case_long$Case)

confidence_long$Confidence_Case <- sub("confidence.", "", confidence_long$Confidence_Case)

# Joining case and confidence data on the Case column after stripping prefix

final_data <- inner_join(case_long, confidence_long, by = c("Researcher", "Case" = "Confidence_Case"))

# Converting 'Decision' from factor to character for manual colour settings

final_data$Decision <- as.character(final_data$Decision)

# Convert 'Case' to a factor with levels ordered numerically

final_data$Case <- factor(final_data$Case, levels = sort(unique(final_data$Case)))

# Ensure the 'Case' column is ordered by extracting numbers and converting them to numeric for sorting

final_data <- final_data %>%

mutate(Case_numeric = as.numeric(str_extract(Case, "\\d+"))) %>%

arrange(Case_numeric) %>%

mutate(Case = factor(Case, levels = unique(Case)))

# Plotting

ggplot(final_data, aes(x = Confidence, y = Case, color = Decision)) +

geom_point(size = 2.5) +

scale_color_manual(values = c("Include" = "#0CB702", "Exclude" = "red")) +

facet_grid(. ~ Researcher) +

labs(x = "Level of confidence in the decision", color = "Decision", y = "Case ID") +

theme_bw()


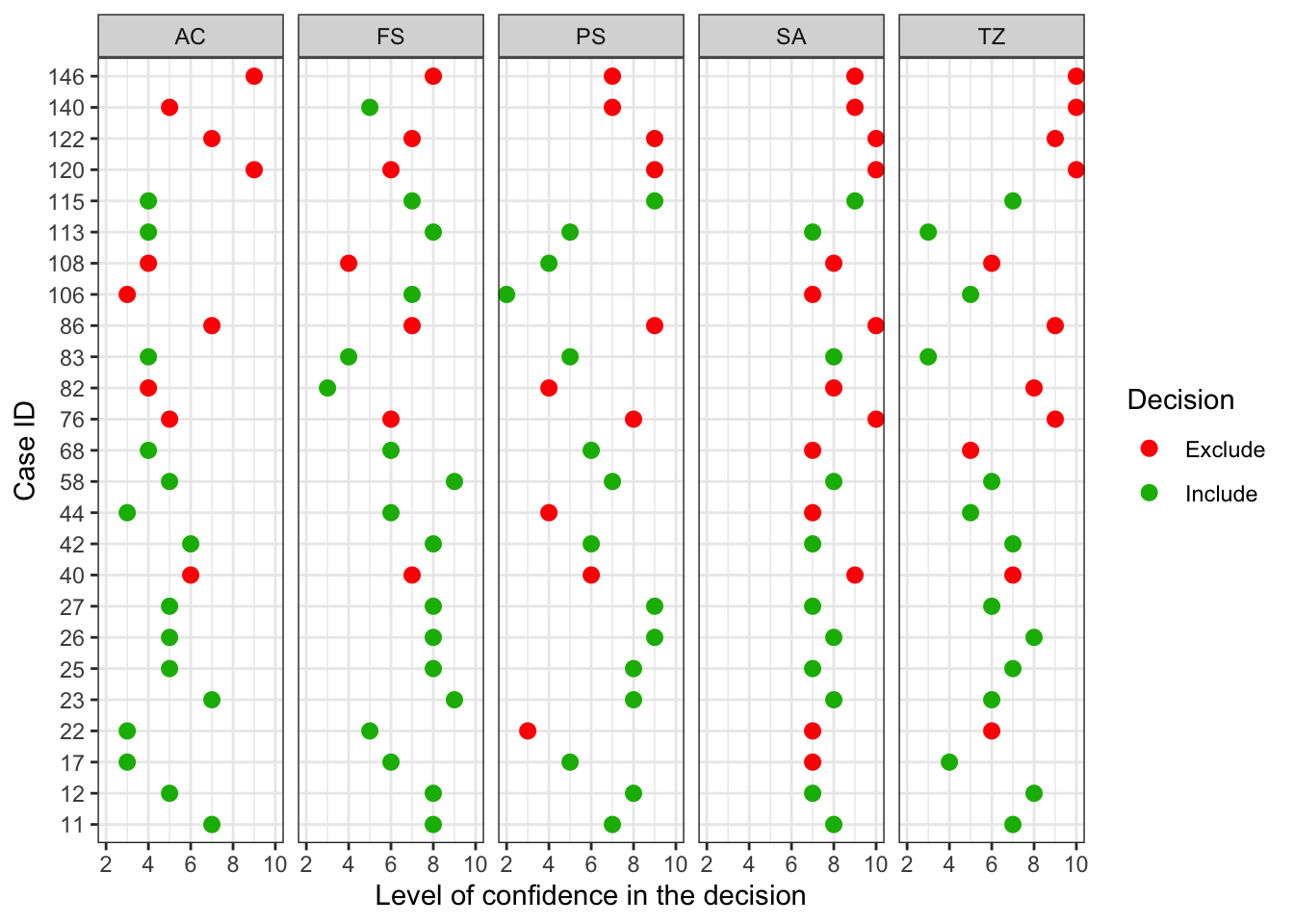


*9.2. Final decision on eligibility*

# Processing the dataset

analysis_results <- final_data %>%

group_by(Case) %>%

summarise(

`Final Decision` = ifelse(sum(Decision == "Include") >= 3, "Include", "Exclude"),

`Agreement Rate` = max(sum(Decision == "Include"), sum(Decision == "Exclude")) / 5 * 100,

`Confidence Level` = mean(Confidence) * 10

) %>%

ungroup()

# Display the results as a kableExtra table

kable(analysis_results, "html") %>%

kable_styling(bootstrap_options = c("striped", "hover", "condensed", "responsive")) %>%

column_spec(1, bold = TRUE) %>%

column_spec(2, width = "100px") %>%

column_spec(3, width = "150px", color = "blue") %>%

column_spec(4, width = "150px", color = "green")

| **Case** | **Final Decision** | **Agreement Rate** | **Confidence Level** |
| --- | --- | --- | --- |
| **11** | Include | 100 | 74 |
| **12** | Include | 100 | 72 |
| **17** | Include | 80 | 50 |
| **22** | Exclude | 60 | 48 |
| **23** | Include | 100 | 76 |
| **25** | Include | 100 | 70 |
| **26** | Include | 100 | 76 |
| **27** | Include | 100 | 70 |
| **40** | Exclude | 100 | 70 |
| **42** | Include | 100 | 68 |
| **44** | Include | 60 | 50 |
| **58** | Include | 100 | 70 |
| **68** | Include | 60 | 56 |
| **76** | Exclude | 100 | 76 |
| **82** | Exclude | 80 | 54 |
| **83** | Include | 100 | 48 |
| **86** | Exclude | 100 | 84 |
| **106** | Include | 60 | 48 |
| **108** | Exclude | 80 | 52 |
| **113** | Include | 100 | 54 |
| **115** | Include | 100 | 72 |
| **120** | Exclude | 100 | 88 |
| **122** | Exclude | 100 | 84 |
| **140** | Exclude | 80 | 72 |
| **146** | Exclude | 100 | 86 |

*9.3. Overall summary of final decision on eligibility*

# Calculating summary statistics

summary_stats <- analysis_results %>%

summarise(

`Total Number of Cases` = n(),

`Number of Cases Included` = sum(`Final Decision` == "Include"),

`Number of Cases Excluded` = sum(`Final Decision` == "Exclude"),

`Overall Confidence for Included Cases` = mean(`Confidence Level`[`Final Decision` == "Include"]),

`Overall Confidence for Excluded Cases` = mean(`Confidence Level`[`Final Decision` == "Exclude"]),

`Overall Agreement for Included Cases` = mean(`Agreement Rate`[`Final Decision` == "Include"]),

`Overall Agreement for Excluded Cases` = mean(`Agreement Rate`[`Final Decision` == "Exclude"])

)

# Display the results as a kableExtra table

kable(summary_stats, "html") %>%

kable_styling(bootstrap_options = c("striped", "hover", "condensed", "responsive")) %>%

column_spec(1, width = "150px") %>%

column_spec(2, width = "150px") %>%

column_spec(3, width = "150px") %>%

column_spec(4, width = "200px") %>%

column_spec(5, width = "200px") %>%

column_spec(6, width = "200px") %>%

column_spec(7, width = "200px")

| **Total Number of Cases** | 25 |
| --- | --- |
| **Number of Cases Included** | 15 |
| **Number of Cases Excluded** | 10 |
| **Overall Confidence for Included Cases** | 63.6 |
| **Overall Confidence for Excluded Cases** | 71.4 |
| **Overall Agreement for Included Cases** | 90.66667 |
| **Overall Agreement for Excluded Cases** | 90 |

**10. Composition of final dataset**

# Remove cases with response bias

# 22, 40, 76, 82, 86, 108, 120, 122, 140, 146.

# Define the case numbers to be removed

cases_to_remove <- c(22, 40, 76, 82, 86, 108, 120, 122, 140, 146)

# Remove rows with the specified row numbers

data3 <- data3 %>%

filter(!N %in% cases_to_remove)

| **Composition of final dataset** | **Number** |
| --- | --- |
| People who expressed interest in learning about the study | 512 |
| People who were not interested after reading the information sheet | 23 |
| People who expressed interest in answering the screening questions | 489 |
| People who did not answer the screening questions | 207 |
| People who answered the screening questionnaire | 282 |
| People who were not eligible to participate | 85 |
| People who were eligible to participate | 197 |
| People who did not respond to the questionnaire | 50 |
| People who responded to the questionnaire | 147 |
| Cases removed due to contradictory information and incomplete questionnaires | 8 |
| Cases removed after researchers’ visual inspection of response patterns | 10 |
| Final number of participants included in the dataset | 129 |
|  |  |

### R Script 2: Psychometric analysis script

**Contents**

This RMarkdown HTML document details the methodology employed to evaluate the psychometric properties of scales used in our study, focusing on internal consistency measures such as Cronbach’s Alpha and Omega. Specifically, the document outlines the R code used to calculate these measures for the entire sample as well as for each specific subgroup within the study: the hoarding group, the homelessness and hoarding group, and the homeless group. These analyses are crucial for ensuring the reliability of the scales across different clinical profiles represented in the sample, thereby underpinning the validity of our findings and supporting the robustness of our conclusions. The scales included in this file are:

- **Patient Health Questionnaire-8**
- **Generalised Anxiety Disorder-7**
- **Work and Social Adjustment Scale**
- **Beliefs about Hoarding Questionnaire**
  - Harm Avoidance
  - Fear of Material Deprivation
  - Attachment Disturbance
- **Savings Inventory-Revised**
  - Clutter
  - Difficulty Discarding
  - Excessive Acquisition
- **Interpersonal Needs Questionnaire**
  - Perceived Burdensomeness
  - Thwarted Belongingness
- **Experiences of Early Material Deprivation Questionnaire**
- **Material Deprivation and Hoarding Beliefs**

**1. Upload of prepared data file**

# Dependencies

library(tidyverse)

library(knitr)

library(TMB)

library(readxl)

library(readr)

library(markdown)

library(kableExtra)

library(htmltools)

library(rmarkdown)

library(summarytools)

library(writexl)

library(pacman)

library(scales)

library(gridExtra)

library(grid)

library(MBESS)

library(lavaan)

library(userfriendlyscience)

library(purrr)

# Parallel processing options are set appropriately for RStudio

# running on macOS with R version 4.0.0 or higher

if (Sys.getenv("RSTUDIO") == "1" && !nzchar(Sys.getenv("RSTUDIO_TERM")) &&

Sys.info()["sysname"] == "Darwin" && getRversion() >= "4.0.0") {

parallel:::setDefaultClusterOptions(setup_strategy = "sequential")}

# Setting workd directory

setwd("~/Library/CloudStorage/Dropbox/DClinPsy/TDRP/Data Analysis")

# Upload dataset

data3 <- read_excel("data3.xlsx") #

**2. Psychometrics**

*2.1. Patient Health Questionnaire-8 (PHQ-8)*

2.1.1. PHQ-8 for the whole sample

# Subsetting subscale

phq <- data3 %>% select(phq.1:phq.8)

## Cronbach's Alpha

phq.alpha_result <- ci.reliability(data = phq, type = "alpha", interval.type = "bonett")

phq.alpha_table <- data.frame(

Estimate = phq.alpha_result$est,

SE = phq.alpha_result$se,

CI_Lower = phq.alpha_result$ci.lower,

CI_Upper = phq.alpha_result$ci.upper

)

## Omega

phq.omega_result <- ci.reliability(data = phq, type = "omega", conf.level = 0.95)

phq.omega_table <- data.frame(

Estimate = phq.omega_result$est,

SE = phq.omega_result$se,

CI_Lower = phq.omega_result$ci.lower,

CI_Upper = phq.omega_result$ci.upper

)

# Combine results into a single table

phq.combined_table <- bind_rows(

data.frame(Measure = "Cronbach's Alpha", phq.alpha_table),

data.frame(Measure = "Omega", phq.omega_table)

)

# Create a kable table

phq.kable_table <- phq.combined_table %>%

kable("html", escape = FALSE, align = "c") %>%

kable_styling(full_width = FALSE)

phq.kable_table

| **Measure** | **Estimate** | **SE** | **CI_Lower** | **CI_Upper** |
| --- | --- | --- | --- | --- |
| Cronbach’s Alpha | 0.8505657 | 0.1341557 | 0.8056235 | 0.8851168 |
| Omega | 0.8532595 | 0.0202071 | 0.8136544 | 0.8928647 |

2.1.2. PHQ-8 for the hoarding group

# Subset items for hoarding group

phq.hd <- data3 %>%

filter(group == "HD") %>%

select(phq.1:phq.8)

## Cronbach's Alpha

phq.hd.alpha_result <- ci.reliability(data = phq.hd, type = "alpha", interval.type = "bonett")

phq.hd.alpha_table <- data.frame(

Estimate = phq.hd.alpha_result$est,

SE = phq.hd.alpha_result$se,

CI_Lower = phq.hd.alpha_result$ci.lower,

CI_Upper = phq.hd.alpha_result$ci.upper

)

## Omega

phq.hd.omega_result <- ci.reliability(data = phq.hd, type = "omega", conf.level = 0.95)

phq.hd.omega_table <- data.frame(

Estimate = phq.hd.omega_result$est,

SE = phq.hd.omega_result$se,

CI_Lower = phq.hd.omega_result$ci.lower,

CI_Upper = phq.hd.omega_result$ci.upper

)

# Combine results into a single table

phq.hd.combined_table <- bind_rows(

data.frame(Measure = "Cronbach's Alpha", phq.hd.alpha_table),

data.frame(Measure = "Omega", phq.hd.omega_table)

)

# Create a kable table

phq.hd.kable_table <- phq.hd.combined_table %>%

kable("html", escape = FALSE, align = "c") %>%

kable_styling(full_width = FALSE)

phq.hd.kable_table

| **Measure** | **Estimate** | **SE** | **CI_Lower** | **CI_Upper** |
| --- | --- | --- | --- | --- |
| Cronbach’s Alpha | 0.8427768 | 0.2361125 | 0.7502555 | 0.9010223 |
| Omega | 0.8429364 | 0.0389089 | 0.7666763 | 0.9191965 |

2.1.3. PHQ-8 for the hoarding and homelessness group

# Subset items for hoarding and homelessness group

phq.h_and_h <- data3 %>%

filter(group == "H&H") %>%

select(phq.1:phq.8)

## Cronbach's Alpha

phq.h_and_h.alpha_result <- ci.reliability(data = phq.h_and_h, type = "alpha", interval.type = "bonett")

phq.h_and_h.alpha_table <- data.frame(

Estimate = phq.h_and_h.alpha_result$est,

SE = phq.h_and_h.alpha_result$se,

CI_Lower = phq.h_and_h.alpha_result$ci.lower,

CI_Upper = phq.h_and_h.alpha_result$ci.upper

)

## Omega

phq.h_and_h.omega_result <- ci.reliability(data = phq.h_and_h, type = "omega", conf.level = 0.95)

phq.h_and_h.omega_table <- data.frame(

Estimate = phq.h_and_h.omega_result$est,

SE = phq.h_and_h.omega_result$se,

CI_Lower = phq.h_and_h.omega_result$ci.lower,

CI_Upper = phq.h_and_h.omega_result$ci.upper

)

# Combine results into a single table

phq.h_and_h.combined_table <- bind_rows(

data.frame(Measure = "Cronbach's Alpha", phq.h_and_h.alpha_table),

data.frame(Measure = "Omega", phq.h_and_h.omega_table)

)

# Create a kable table

phq.h_and_h.kable_table <- phq.h_and_h.combined_table %>%

kable("html", escape = FALSE, align = "c") %>%

kable_styling(full_width = FALSE)

phq.h_and_h.kable_table

| **Measure** | **Estimate** | **SE** | **CI_Lower** | **CI_Upper** |
| --- | --- | --- | --- | --- |
| Cronbach’s Alpha | 0.8239778 | 0.2253745 | 0.7262169 | 0.8868308 |
| Omega | 0.8337913 | 0.0391556 | 0.7570476 | 0.9105350 |

2.1.3. PHQ-8 for the hoarding and homelessness group

# Subset items for hoarding and homelessness group

phq.h_and_h <- data3 %>%

filter(group == "H&H") %>%

select(phq.1:phq.8)

## Cronbach's Alpha

phq.h_and_h.alpha_result <- ci.reliability(data = phq.h_and_h, type = "alpha", interval.type = "bonett")

phq.h_and_h.alpha_table <- data.frame(

Estimate = phq.h_and_h.alpha_result$est,

SE = phq.h_and_h.alpha_result$se,

CI_Lower = phq.h_and_h.alpha_result$ci.lower,

CI_Upper = phq.h_and_h.alpha_result$ci.upper

)

## Omega

phq.h_and_h.omega_result <- ci.reliability(data = phq.h_and_h, type = "omega", conf.level = 0.95)

phq.h_and_h.omega_table <- data.frame(

Estimate = phq.h_and_h.omega_result$est,

SE = phq.h_and_h.omega_result$se,

CI_Lower = phq.h_and_h.omega_result$ci.lower,

CI_Upper = phq.h_and_h.omega_result$ci.upper

)

# Combine results into a single table

phq.h_and_h.combined_table <- bind_rows(

data.frame(Measure = "Cronbach's Alpha", phq.h_and_h.alpha_table),

data.frame(Measure = "Omega", phq.h_and_h.omega_table)

)

# Create a kable table

phq.h_and_h.kable_table <- phq.h_and_h.combined_table %>%

kable("html", escape = FALSE, align = "c") %>%

kable_styling(full_width = FALSE)

phq.h_and_h.kable_table

| **Measure** | **Estimate** | **SE** | **CI_Lower** | **CI_Upper** |
| --- | --- | --- | --- | --- |
| Cronbach’s Alpha | 0.8239778 | 0.2253745 | 0.7262169 | 0.8868308 |
| Omega | 0.8337913 | 0.0391556 | 0.7570476 | 0.9105350 |

2.1.4. PHQ-8 for the homelessness group

# Subset items for the homelessness group

phq.hm <- data3 %>%

filter(group == "HM") %>%

select(phq.1:phq.8)

## Cronbach's Alpha

phq.hm.alpha_result <- ci.reliability(data = phq.hm, type = "alpha", interval.type = "bonett")

phq.hm.alpha_table <- data.frame(

Estimate = phq.hm.alpha_result$est,

SE = phq.hm.alpha_result$se,

CI_Lower = phq.hm.alpha_result$ci.lower,

CI_Upper = phq.hm.alpha_result$ci.upper

)

## Omega

phq.hm.omega_result <- ci.reliability(data = phq.hm, type = "omega", conf.level = 0.95)

phq.hm.omega_table <- data.frame(

Estimate = phq.hm.omega_result$est,

SE = phq.hm.omega_result$se,

CI_Lower = phq.hm.omega_result$ci.lower,

CI_Upper = phq.hm.omega_result$ci.upper

)

# Combine results into a single table

phq.hm.combined_table <- bind_rows(

data.frame(Measure = "Cronbach's Alpha", phq.hm.alpha_table),

data.frame(Measure = "Omega", phq.hm.omega_table)

)

# Create a kable table

phq.hm.kable_table <- phq.hm.combined_table %>%

kable("html", escape = FALSE, align = "c") %>%

kable_styling(full_width = FALSE)

phq.hm.kable_table

| **Measure** | **Estimate** | **SE** | **CI_Lower** | **CI_Upper** |
| --- | --- | --- | --- | --- |
| Cronbach’s Alpha | 0.8526813 | 0.2485479 | 0.7602149 | 0.9094906 |
| Omega | 0.8585430 | 0.0341858 | 0.7915401 | 0.9255459 |

*2.2. Generalised Anxiety Disorder-7 (GAD-7)*

2.2.1. GAD-7 for the whole sample

# Subsetting subscale

gad <- data3 %>% select(gad.1:gad.7)

## Cronbach's Alpha

gad.alpha_result <- ci.reliability(data = gad, type = "alpha", interval.type = "bonett")

gad.alpha_table <- data.frame(

Estimate = gad.alpha_result$est,

SE = gad.alpha_result$se,

CI_Lower = gad.alpha_result$ci.lower,

CI_Upper = gad.alpha_result$ci.upper

)

## Omega

gad.omega_result <- ci.reliability(data = gad, type = "omega", conf.level = 0.95)

gad.omega_table <- data.frame(

Estimate = gad.omega_result$est,

SE = gad.omega_result$se,

CI_Lower = gad.omega_result$ci.lower,

CI_Upper = gad.omega_result$ci.upper

)

# Combine results into a single table

gad.combined_table <- bind_rows(

data.frame(Measure = "Cronbach's Alpha", gad.alpha_table),

data.frame(Measure = "Omega", gad.omega_table)

)

# Create a kable table

gad.kable_table <- gad.combined_table %>%

kable("html", escape = FALSE, align = "c") %>%

kable_styling(full_width = FALSE)

gad.kable_table

| **Measure** | **Estimate** | **SE** | **CI_Lower** | **CI_Upper** |
| --- | --- | --- | --- | --- |
| Cronbach’s Alpha | 0.8600664 | 0.1355459 | 0.8174848 | 0.8927135 |
| Omega | 0.8670728 | 0.0192971 | 0.8292511 | 0.9048945 |

2.2.2. GAD-7 for the hoarding group

# Subset items for hoarding group

gad.hd <- data3 %>%

filter(group == "HD") %>%

select(gad.1:gad.7)

## Cronbach's Alpha

gad.hd.alpha_result <- ci.reliability(data = gad.hd, type = "alpha", interval.type = "bonett")

gad.hd.alpha_table <- data.frame(

Estimate = gad.hd.alpha_result$est,

SE = gad.hd.alpha_result$se,

CI_Lower = gad.hd.alpha_result$ci.lower,

CI_Upper = gad.hd.alpha_result$ci.upper

)

## Omega

gad.hd.omega_result <- ci.reliability(data = gad.hd, type = "omega", conf.level = 0.95)

gad.hd.omega_table <- data.frame(

Estimate = gad.hd.omega_result$est,

SE = gad.hd.omega_result$se,

CI_Lower = gad.hd.omega_result$ci.lower,

CI_Upper = gad.hd.omega_result$ci.upper

)

# Combine results into a single table

gad.hd.combined_table <- bind_rows(

data.frame(Measure = "Cronbach's Alpha", gad.hd.alpha_table),

data.frame(Measure = "Omega", gad.hd.omega_table)

)

# Create a kable table

gad.hd.kable_table <- gad.hd.combined_table %>%

kable("html", escape = FALSE, align = "c") %>%

kable_styling(full_width = FALSE)

gad.hd.kable_table

| **Measure** | **Estimate** | **SE** | **CI_Lower** | **CI_Upper** |
| --- | --- | --- | --- | --- |
| Cronbach’s Alpha | 0.8332705 | 0.2385594 | 0.7338818 | 0.8955399 |
| Omega | 0.8496087 | 0.0393249 | 0.7725333 | 0.9266840 |

2.2.3. GAD-7 for the hoarding and homelessness group

# Subset items for hoarding and homelessness group

gad.h_and_h <- data3 %>%

filter(group == "H&H") %>%

select(gad.1:gad.7)

## Cronbach's Alpha

gad.h_and_h.alpha_result <- ci.reliability(data = gad.h_and_h, type = "alpha", interval.type = "bonett")

gad.h_and_h.alpha_table <- data.frame(

Estimate = gad.h_and_h.alpha_result$est,

SE = gad.h_and_h.alpha_result$se,

CI_Lower = gad.h_and_h.alpha_result$ci.lower,

CI_Upper = gad.h_and_h.alpha_result$ci.upper

)

## Omega

gad.h_and_h.omega_result <- ci.reliability(data = gad.h_and_h, type = "omega", conf.level = 0.95)

gad.h_and_h.omega_table <- data.frame(

Estimate = gad.h_and_h.omega_result$est,

SE = gad.h_and_h.omega_result$se,

CI_Lower = gad.h_and_h.omega_result$ci.lower,

CI_Upper = gad.h_and_h.omega_result$ci.upper

)

# Combine results into a single table

gad.h_and_h.combined_table <- bind_rows(

data.frame(Measure = "Cronbach's Alpha", gad.h_and_h.alpha_table),

data.frame(Measure = "Omega", gad.h_and_h.omega_table)

)

# Create a kable table

gad.h_and_h.kable_table <- gad.h_and_h.combined_table %>%

kable("html", escape = FALSE, align = "c") %>%

kable_styling(full_width = FALSE)

gad.h_and_h.kable_table

| **Measure** | **Estimate** | **SE** | **CI_Lower** | **CI_Upper** |
| --- | --- | --- | --- | --- |
| Cronbach’s Alpha | 0.8270002 | 0.2277100 | 0.7296834 | 0.8892819 |
| Omega | 0.8362612 | 0.0402911 | 0.7572920 | 0.9152303 |

2.2.4. GAD-7 for the homelessness group

# Subset items for the homelessness group

gad.hm <- data3 %>%

filter(group == "HM") %>%

select(gad.1:gad.7)

## Cronbach's Alpha

gad.hm.alpha_result <- ci.reliability(data = gad.hm, type = "alpha", interval.type = "bonett")

gad.hm.alpha_table <- data.frame(

Estimate = gad.hm.alpha_result$est,

SE = gad.hm.alpha_result$se,

CI_Lower = gad.hm.alpha_result$ci.lower,

CI_Upper = gad.hm.alpha_result$ci.upper

)

## Omega

gad.hm.omega_result <- ci.reliability(data = gad.hm, type = "omega", conf.level = 0.95)

gad.hm.omega_table <- data.frame(

Estimate = gad.hm.omega_result$est,

SE = gad.hm.omega_result$se,

CI_Lower = gad.hm.omega_result$ci.lower,

CI_Upper = gad.hm.omega_result$ci.upper

)

# Combine results into a single table

gad.hm.combined_table <- bind_rows(

data.frame(Measure = "Cronbach's Alpha", gad.hm.alpha_table),

data.frame(Measure = "Omega", gad.hm.omega_table)

)

# Create a kable table

gad.hm.kable_table <- gad.hm.combined_table %>%

kable("html", escape = FALSE, align = "c") %>%

kable_styling(full_width = FALSE)

gad.hm.kable_table

| **Measure** | **Estimate** | **SE** | **CI_Lower** | **CI_Upper** |
| --- | --- | --- | --- | --- |
| Cronbach’s Alpha | 0.8520138 | 0.2511236 | 0.7579094 | 0.9095384 |
| Omega | 0.8618983 | 0.0390517 | 0.7853583 | 0.9384382 |

*2.3. Work and Social Adjustment Scale (WSAJ)*

2.3.1. WSAJ for the whole sample

# Subsetting subscale

wsaj <- data3 %>% select(wsaj.1:wsaj.5)

## Cronbach's Alpha

wsaj.alpha_result <- ci.reliability(data = wsaj, type = "alpha", interval.type = "bonett")

wsaj.alpha_table <- data.frame(

Estimate = wsaj.alpha_result$est,

SE = wsaj.alpha_result$se,

CI_Lower = wsaj.alpha_result$ci.lower,

CI_Upper = wsaj.alpha_result$ci.upper

)

## Omega

wsaj.omega_result <- ci.reliability(data = wsaj, type = "omega", conf.level = 0.95)

wsaj.omega_table <- data.frame(

Estimate = wsaj.omega_result$est,

SE = wsaj.omega_result$se,

CI_Lower = wsaj.omega_result$ci.lower,

CI_Upper = wsaj.omega_result$ci.upper

)

# Combine results into a single table

wsaj.combined_table <- bind_rows(

data.frame(Measure = "Cronbach's Alpha", wsaj.alpha_table),

data.frame(Measure = "Omega", wsaj.omega_table)

)

# Create a kable table

wsaj.kable_table <- wsaj.combined_table %>%

kable("html", escape = FALSE, align = "c") %>%

kable_styling(full_width = FALSE)

wsaj.kable_table

| **Measure** | **Estimate** | **SE** | **CI_Lower** | **CI_Upper** |
| --- | --- | --- | --- | --- |
| Cronbach’s Alpha | 0.7431460 | 0.1403034 | 0.6618473 | 0.8048988 |
| Omega | 0.7481318 | 0.0390763 | 0.6715437 | 0.8247200 |

2.3.2. WSAJ for the hoarding group

# Subset items for hoarding group

wsaj.hd <- data3 %>%

filter(group == "HD") %>%

select(wsaj.1:wsaj.5)

## Cronbach's Alpha

wsaj.hd.alpha_result <- ci.reliability(data = wsaj.hd, type = "alpha", interval.type = "bonett")

wsaj.hd.alpha_table <- data.frame(

Estimate = wsaj.hd.alpha_result$est,

SE = wsaj.hd.alpha_result$se,

CI_Lower = wsaj.hd.alpha_result$ci.lower,

CI_Upper = wsaj.hd.alpha_result$ci.upper

)

## Omega

wsaj.hd.omega_result <- ci.reliability(data = wsaj.hd, type = "omega", conf.level = 0.95)

wsaj.hd.omega_table <- data.frame(

Estimate = wsaj.hd.omega_result$est,

SE = wsaj.hd.omega_result$se,

CI_Lower = wsaj.hd.omega_result$ci.lower,

CI_Upper = wsaj.hd.omega_result$ci.upper

)

# Combine results into a single table

wsaj.hd.combined_table <- bind_rows(

data.frame(Measure = "Cronbach's Alpha", wsaj.hd.alpha_table),

data.frame(Measure = "Omega", wsaj.hd.omega_table)

)

# Create a kable table

wsaj.hd.kable_table <- wsaj.hd.combined_table %>%

kable("html", escape = FALSE, align = "c") %>%

kable_styling(full_width = FALSE)

wsaj.hd.kable_table

| **Measure** | **Estimate** | **SE** | **CI_Lower** | **CI_Upper** |
| --- | --- | --- | --- | --- |
| Cronbach’s Alpha | 0.7618089 | 0.2469324 | 0.6135309 | 0.8531965 |
| Omega | 0.7687991 | 0.0638154 | 0.6437233 | 0.8938749 |

2.3.3. WSAJ for the hoarding and homelessness group

# Subset items for hoarding and homelessness group

wsaj.h_and_h <- data3 %>%

filter(group == "H&H") %>%

select(wsaj.1:wsaj.5)

## Cronbach's Alpha

wsaj.h_and_h.alpha_result <- ci.reliability(data = wsaj.h_and_h, type = "alpha", interval.type = "bonett")

wsaj.h_and_h.alpha_table <- data.frame(

Estimate = wsaj.h_and_h.alpha_result$est,

SE = wsaj.h_and_h.alpha_result$se,

CI_Lower = wsaj.h_and_h.alpha_result$ci.lower,

CI_Upper = wsaj.h_and_h.alpha_result$ci.upper

)

## Omega

wsaj.h_and_h.omega_result <- ci.reliability(data = wsaj.h_and_h, type = "omega", conf.level = 0.95)

wsaj.h_and_h.omega_table <- data.frame(

Estimate = wsaj.h_and_h.omega_result$est,

SE = wsaj.h_and_h.omega_result$se,

CI_Lower = wsaj.h_and_h.omega_result$ci.lower,

CI_Upper = wsaj.h_and_h.omega_result$ci.upper

)

# Combine results into a single table

wsaj.h_and_h.combined_table <- bind_rows(

data.frame(Measure = "Cronbach's Alpha", wsaj.h_and_h.alpha_table),

data.frame(Measure = "Omega", wsaj.h_and_h.omega_table)

)

# Create a kable table

wsaj.h_and_h.kable_table <- wsaj.h_and_h.combined_table %>%

kable("html", escape = FALSE, align = "c") %>%

kable_styling(full_width = FALSE)

wsaj.h_and_h.kable_table

| **Measure** | **Estimate** | **SE** | **CI_Lower** | **CI_Upper** |
| --- | --- | --- | --- | --- |
| Cronbach’s Alpha | 0.6925013 | 0.2357023 | 0.5119397 | 0.8062627 |
| Omega | 0.7070273 | 0.0755324 | 0.5589864 | 0.8550682 |

2.3.4. WSAJ for the homelessness group

# Subset items for the homelessness group

wsaj.hm <- data3 %>%

filter(group == "HM") %>%

select(wsaj.1:wsaj.5)

## Cronbach's Alpha

wsaj.hm.alpha_result <- ci.reliability(data = wsaj.hm, type = "alpha", interval.type = "bonett")

wsaj.hm.alpha_table <- data.frame(

Estimate = wsaj.hm.alpha_result$est,

SE = wsaj.hm.alpha_result$se,

CI_Lower = wsaj.hm.alpha_result$ci.lower,

CI_Upper = wsaj.hm.alpha_result$ci.upper

)

## Omega

wsaj.hm.omega_result <- ci.reliability(data = wsaj.hm, type = "omega", conf.level = 0.95)

wsaj.hm.omega_table <- data.frame(

Estimate = wsaj.hm.omega_result$est,

SE = wsaj.hm.omega_result$se,

CI_Lower = wsaj.hm.omega_result$ci.lower,

CI_Upper = wsaj.hm.omega_result$ci.upper

)

# Combine results into a single table

wsaj.hm.combined_table <- bind_rows(

data.frame(Measure = "Cronbach's Alpha", wsaj.hm.alpha_table),

data.frame(Measure = "Omega", wsaj.hm.omega_table)

)

# Create a kable table

wsaj.hm.kable_table <- wsaj.hm.combined_table %>%

kable("html", escape = FALSE, align = "c") %>%

kable_styling(full_width = FALSE)

wsaj.hm.kable_table

| **Measure** | **Estimate** | **SE** | **CI_Lower** | **CI_Upper** |
| --- | --- | --- | --- | --- |
| Cronbach’s Alpha | 0.7474405 | 0.2599376 | 0.5796385 | 0.8482585 |
| Omega | 0.7628890 | 0.0616709 | 0.6420162 | 0.8837618 |

*2.4. Beliefs about Hoarding Questionnaire: Harm Avoidance*

2.4.1. Harm Avoidance for the whole sample

# Subsetting subscale

bah.harm.avoi <- data3 %>% select(BAH.6, BAH.12, BAH.21, BAH.24, BAH.25, BAH.28)

## Cronbach's Alpha

bah.ha.alpha_result <- ci.reliability(data = bah.harm.avoi, type = "alpha", interval.type = "bonett")

bah.ha.alpha_table <- data.frame(

Estimate = bah.ha.alpha_result$est,

SE = bah.ha.alpha_result$se,

CI_Lower = bah.ha.alpha_result$ci.lower,

CI_Upper = bah.ha.alpha_result$ci.upper

)

## Omega

bah.ha.omega_result <- ci.reliability(data = bah.harm.avoi, type = "omega", conf.level = 0.95)

bah.ha.omega_table <- data.frame(

Estimate = bah.ha.omega_result$est,

SE = bah.ha.omega_result$se,

CI_Lower = bah.ha.omega_result$ci.lower,

CI_Upper = bah.ha.omega_result$ci.upper

)

# Combine results into a single table

bah.ha.combined_table <- bind_rows(

data.frame(Measure = "Cronbach's Alpha", bah.ha.alpha_table),

data.frame(Measure = "Omega", bah.ha.omega_table)

)

# Create a kable table

bah.ha.kable_table <- bah.ha.combined_table %>%

kable("html", escape = FALSE, align = "c") %>%

kable_styling(full_width = FALSE)

bah.ha.kable_table

| **Measure** | **Estimate** | **SE** | **CI_Lower** | **CI_Upper** |
| --- | --- | --- | --- | --- |
| Cronbach’s Alpha | 0.8122942 | 0.1374687 | 0.7542512 | 0.8566280 |
| Omega | 0.8111878 | 0.0251552 | 0.7618844 | 0.8604912 |

2.4.2. Harm Avoidance for the hoarding group

# Subset items for hoarding group

bah.ha.hd <- data3 %>%

filter(group == "HD") %>%

select(BAH.6, BAH.12, BAH.21, BAH.24, BAH.25, BAH.28)

## Cronbach's Alpha

bah.ha.hd.alpha_result <- ci.reliability(data = bah.ha.hd, type = "alpha", interval.type = "bonett")

bah.ha.hd.alpha_table <- data.frame(

Estimate = bah.ha.hd.alpha_result$est,

SE = bah.ha.hd.alpha_result$se,

CI_Lower = bah.ha.hd.alpha_result$ci.lower,

CI_Upper = bah.ha.hd.alpha_result$ci.upper

)

## Omega

bah.ha.hd.omega_result <- ci.reliability(data = bah.ha.hd, type = "omega", conf.level = 0.95)

bah.ha.hd.omega_table <- data.frame(

Estimate = bah.ha.hd.omega_result$est,

SE = bah.ha.hd.omega_result$se,

CI_Lower = bah.ha.hd.omega_result$ci.lower,

CI_Upper = bah.ha.hd.omega_result$ci.upper

)

# Combine results into a single table

bah.ha.hd.combined_table <- bind_rows(

data.frame(Measure = "Cronbach's Alpha", bah.ha.hd.alpha_table),

data.frame(Measure = "Omega", bah.ha.hd.omega_table)

)

# Create a kable table

bah.ha.hd.kable_table <- bah.ha.hd.combined_table %>%

kable("html", escape = FALSE, align = "c") %>%

kable_styling(full_width = FALSE)

bah.ha.hd.kable_table

| **Measure** | **Estimate** | **SE** | **CI_Lower** | **CI_Upper** |
| --- | --- | --- | --- | --- |
| Cronbach’s Alpha | 0.7149118 | 0.2419434 | 0.5419405 | 0.8225660 |
| Omega | 0.6269305 | 0.0948981 | 0.4409338 | 0.8129273 |

2.4.3. Harm Avoidance for the hoarding and homelessness group

# Subset items for hoarding and homelessness group

bah.ha.h_and_h <- data3 %>%

filter(group == "H&H") %>%

select(BAH.6, BAH.12, BAH.21, BAH.24, BAH.25, BAH.28)

## Cronbach's Alpha

bah.ha.h_and_h.alpha_result <- ci.reliability(data = bah.ha.h_and_h, type = "alpha", interval.type = "bonett")

bah.ha.h_and_h.alpha_table <- data.frame(

Estimate = bah.ha.h_and_h.alpha_result$est,

SE = bah.ha.h_and_h.alpha_result$se,

CI_Lower = bah.ha.h_and_h.alpha_result$ci.lower,

CI_Upper = bah.ha.h_and_h.alpha_result$ci.upper

)

## Omega

bah.ha.h_and_h.omega_result <- ci.reliability(data = bah.ha.h_and_h, type = "omega", conf.level = 0.95)

bah.ha.h_and_h.omega_table <- data.frame(

Estimate = bah.ha.h_and_h.omega_result$est,

SE = bah.ha.h_and_h.omega_result$se,

CI_Lower = bah.ha.h_and_h.omega_result$ci.lower,

CI_Upper = bah.ha.h_and_h.omega_result$ci.upper

)

# Combine results into a single table

bah.ha.h_and_h.combined_table <- bind_rows(

data.frame(Measure = "Cronbach's Alpha", bah.ha.h_and_h.alpha_table),

data.frame(Measure = "Omega", bah.ha.h_and_h.omega_table)

)

# Create a kable table

bah.ha.h_and_h.kable_table <- bah.ha.h_and_h.combined_table %>%

kable("html", escape = FALSE, align = "c") %>%

kable_styling(full_width = FALSE)

bah.ha.h_and_h.kable_table

| **Measure** | **Estimate** | **SE** | **CI_Lower** | **CI_Upper** |
| --- | --- | --- | --- | --- |
| Cronbach’s Alpha | 0.8188372 | 0.2309401 | 0.7151308 | 0.8847894 |
| Omega | 0.8266080 | 0.0365242 | 0.7550220 | 0.8981941 |

2.4.4. Harm Avoidance for the homelessness group

# Subset items for the homelessness group

bah.ha.hm <- data3 %>%

filter(group == "HM") %>%

select(BAH.6, BAH.12, BAH.21, BAH.24, BAH.25, BAH.28)

## Cronbach's Alpha

bah.ha.hm.alpha_result <- ci.reliability(data = bah.ha.hm, type = "alpha", interval.type = "bonett")

bah.ha.hm.alpha_table <- data.frame(

Estimate = bah.ha.hm.alpha_result$est,

SE = bah.ha.hm.alpha_result$se,

CI_Lower = bah.ha.hm.alpha_result$ci.lower,

CI_Upper = bah.ha.hm.alpha_result$ci.upper

)

## Omega

bah.ha.hm.omega_result <- ci.reliability(data = bah.ha.hm, type = "omega", conf.level = 0.95)

bah.ha.hm.omega_table <- data.frame(

Estimate = bah.ha.hm.omega_result$est,

SE = bah.ha.hm.omega_result$se,

CI_Lower = bah.ha.hm.omega_result$ci.lower,

CI_Upper = bah.ha.hm.omega_result$ci.upper

)

# Combine results into a single table

bah.ha.hm.combined_table <- bind_rows(

data.frame(Measure = "Cronbach's Alpha", bah.ha.hm.alpha_table),

data.frame(Measure = "Omega", bah.ha.hm.omega_table)

)

# Create a kable table

bah.ha.hm.kable_table <- bah.ha.hm.combined_table %>%

kable("html", escape = FALSE, align = "c") %>%

kable_styling(full_width = FALSE)

bah.ha.hm.kable_table

| **Measure** | **Estimate** | **SE** | **CI_Lower** | **CI_Upper** |
| --- | --- | --- | --- | --- |
| Cronbach’s Alpha | 0.7921448 | 0.2546858 | 0.6575873 | 0.8738254 |
| Omega | 0.8016355 | 0.0522072 | 0.6993114 | 0.9039597 |

*2.5. Beliefs about Hoarding Questionnaire: Fear of Material Deprivation*

2.5.1. Fear of Material Deprivation for the whole sample

# Subsetting subscale

bah.fmd <- data3 %>% select(BAH.1, BAH.5, BAH.7, BAH.10, BAH.13, BAH.15, BAH.18, BAH.23, BAH.27)

## Cronbach's Alpha

bah.fmd.alpha_result <- ci.reliability(data = bah.fmd, type = "alpha", interval.type = "bonett")

bah.fmd.alpha_table <- data.frame(

Estimate = bah.fmd.alpha_result$est,

SE = bah.fmd.alpha_result$se,

CI_Lower = bah.fmd.alpha_result$ci.lower,

CI_Upper = bah.fmd.alpha_result$ci.upper

)

## Omega

bah.fmd.omega_result <- ci.reliability(data = bah.fmd, type = "omega", conf.level = 0.95)

bah.fmd.omega_table <- data.frame(

Estimate = bah.fmd.omega_result$est,

SE = bah.fmd.omega_result$se,

CI_Lower = bah.fmd.omega_result$ci.lower,

CI_Upper = bah.fmd.omega_result$ci.upper

)

# Combine results into a single table

bah.fmd.combined_table <- bind_rows(

data.frame(Measure = "Cronbach's Alpha", bah.fmd.alpha_table),

data.frame(Measure = "Omega", bah.fmd.omega_table)

)

# Create a kable table

bah.fmd.kable_table <- bah.fmd.combined_table %>%

kable("html", escape = FALSE, align = "c") %>%

kable_styling(full_width = FALSE)

bah.fmd.kable_table

| **Measure** | **Estimate** | **SE** | **CI_Lower** | **CI_Upper** |
| --- | --- | --- | --- | --- |
| Cronbach’s Alpha | 0.8734141 | 0.1331035 | 0.8356827 | 0.9024814 |
| Omega | 0.8751034 | 0.0174687 | 0.8408655 | 0.9093414 |

2.5.2. Fear of Material Deprivation for the hoarding group

# Subset items for hoarding group

bah.fmd.hd <- data3 %>%

filter(group == "HD") %>%

select(BAH.1, BAH.5, BAH.7, BAH.10, BAH.13, BAH.15, BAH.18, BAH.23, BAH.27)

## Cronbach's Alpha

bah.fmd.hd.alpha_result <- ci.reliability(data = bah.fmd.hd, type = "alpha", interval.type = "bonett")

bah.fmd.hd.alpha_table <- data.frame(

Estimate = bah.fmd.hd.alpha_result$est,

SE = bah.fmd.hd.alpha_result$se,

CI_Lower = bah.fmd.hd.alpha_result$ci.lower,

CI_Upper = bah.fmd.hd.alpha_result$ci.upper

)

## Omega

bah.fmd.hd.omega_result <- ci.reliability(data = bah.fmd.hd, type = "omega", conf.level = 0.95)

bah.fmd.hd.omega_table <- data.frame(

Estimate = bah.fmd.hd.omega_result$est,

SE = bah.fmd.hd.omega_result$se,

CI_Lower = bah.fmd.hd.omega_result$ci.lower,

CI_Upper = bah.fmd.hd.omega_result$ci.upper

)

# Combine results into a single table

bah.fmd.hd.combined_table <- bind_rows(

data.frame(Measure = "Cronbach's Alpha", bah.fmd.hd.alpha_table),

data.frame(Measure = "Omega", bah.fmd.hd.omega_table)

)

# Create a kable table

bah.fmd.hd.kable_table <- bah.fmd.hd.combined_table %>%

kable("html", escape = FALSE, align = "c") %>%

kable_styling(full_width = FALSE)

bah.fmd.hd.kable_table

| **Measure** | **Estimate** | **SE** | **CI_Lower** | **CI_Upper** |
| --- | --- | --- | --- | --- |
| Cronbach’s Alpha | 0.8676165 | 0.2342606 | 0.7904745 | 0.9163567 |
| Omega | 0.8672380 | 0.0335053 | 0.8015689 | 0.9329072 |

2.5.3. Fear of Material Deprivation for the hoarding and homelessness group

# Subset items for hoarding and homelessness group

bah.fmd.h_and_h <- data3 %>%

filter(group == "H&H") %>%

select(BAH.1, BAH.5, BAH.7, BAH.10, BAH.13, BAH.15, BAH.18, BAH.23, BAH.27)

## Cronbach's Alpha

bah.fmd.h_and_h.alpha_result <- ci.reliability(data = bah.fmd.h_and_h, type = "alpha", interval.type = "bonett")

bah.fmd.h_and_h.alpha_table <- data.frame(

Estimate = bah.fmd.h_and_h.alpha_result$est,

SE = bah.fmd.h_and_h.alpha_result$se,

CI_Lower = bah.fmd.h_and_h.alpha_result$ci.lower,

CI_Upper = bah.fmd.h_and_h.alpha_result$ci.upper

)

## Omega

bah.fmd.h_and_h.omega_result <- ci.reliability(data = bah.fmd.h_and_h, type = "omega", conf.level = 0.95)

bah.fmd.h_and_h.omega_table <- data.frame(

Estimate = bah.fmd.h_and_h.omega_result$est,

SE = bah.fmd.h_and_h.omega_result$se,

CI_Lower = bah.fmd.h_and_h.omega_result$ci.lower,

CI_Upper = bah.fmd.h_and_h.omega_result$ci.upper

)

# Combine results into a single table

bah.fmd.h_and_h.combined_table <- bind_rows(

data.frame(Measure = "Cronbach's Alpha", bah.fmd.h_and_h.alpha_table),

data.frame(Measure = "Omega", bah.fmd.h_and_h.omega_table)

)

# Create a kable table

bah.fmd.h_and_h.kable_table <- bah.fmd.h_and_h.combined_table %>%

kable("html", escape = FALSE, align = "c") %>%

kable_styling(full_width = FALSE)

bah.fmd.h_and_h.kable_table

| **Measure** | **Estimate** | **SE** | **CI_Lower** | **CI_Upper** |
| --- | --- | --- | --- | --- |
| Cronbach’s Alpha | 0.8482803 | 0.2236068 | 0.7648330 | 0.9021170 |
| Omega | 0.8545645 | 0.0325285 | 0.7908098 | 0.9183193 |

2.5.4. Fear of Material Deprivation for the homelessness group

# Subset items for the homelessness group

bah.fmd.hm <- data3 %>%

filter(group == "HM") %>%

select(BAH.1, BAH.5, BAH.7, BAH.10, BAH.13, BAH.15, BAH.18, BAH.23, BAH.27)

## Cronbach's Alpha

bah.fmd.hm.alpha_result <- ci.reliability(data = bah.fmd.hm, type = "alpha", interval.type = "bonett")

bah.fmd.hm.alpha_table <- data.frame(

Estimate = bah.fmd.hm.alpha_result$est,

SE = bah.fmd.hm.alpha_result$se,

CI_Lower = bah.fmd.hm.alpha_result$ci.lower,

CI_Upper = bah.fmd.hm.alpha_result$ci.upper

)

## Omega

bah.fmd.hm.omega_result <- ci.reliability(data = bah.fmd.hm, type = "omega", conf.level = 0.95)

bah.fmd.hm.omega_table <- data.frame(

Estimate = bah.fmd.hm.omega_result$est,

SE = bah.fmd.hm.omega_result$se,

CI_Lower = bah.fmd.hm.omega_result$ci.lower,

CI_Upper = bah.fmd.hm.omega_result$ci.upper

)

# Combine results into a single table

bah.fmd.hm.combined_table <- bind_rows(

data.frame(Measure = "Cronbach's Alpha", bah.fmd.hm.alpha_table),

data.frame(Measure = "Omega", bah.fmd.hm.omega_table)

)

# Create a kable table

bah.fmd.hm.kable_table <- bah.fmd.hm.combined_table %>%

kable("html", escape = FALSE, align = "c") %>%

kable_styling(full_width = FALSE)

bah.fmd.hm.kable_table

| **Measure** | **Estimate** | **SE** | **CI_Lower** | **CI_Upper** |
| --- | --- | --- | --- | --- |
| Cronbach’s Alpha | 0.8266833 | 0.2465985 | 0.7189747 | 0.8931104 |
| Omega | 0.8463314 | 0.0384484 | 0.7709740 | 0.9216888 |

*2.6. Beliefs about Hoarding Questionnaire: Attachment Disturbance*

2.6.1. Attachment Disturbance for the whole sample

# Subsetting subscale

bah.attd <- data3 %>% select(BAH.2, BAH.3, BAH.4, BAH.8, BAH.11, BAH.14, BAH.16, BAH.17, BAH.19, BAH.20, BAH.22, BAH.27)

## Cronbach's Alpha

bah.attd.alpha_result <- ci.reliability(data = bah.attd, type = "alpha", interval.type = "bonett")

bah.attd.alpha_table <- data.frame(

Estimate = bah.attd.alpha_result$est,

SE = bah.attd.alpha_result$se,

CI_Lower = bah.attd.alpha_result$ci.lower,

CI_Upper = bah.attd.alpha_result$ci.upper

)

## Omega

bah.attd.omega_result <- ci.reliability(data = bah.attd, type = "omega", conf.level = 0.95)

bah.attd.omega_table <- data.frame(

Estimate = bah.attd.omega_result$est,

SE = bah.attd.omega_result$se,

CI_Lower = bah.attd.omega_result$ci.lower,

CI_Upper = bah.attd.omega_result$ci.upper

)

# Combine results into a single table

bah.attd.combined_table <- bind_rows(

data.frame(Measure = "Cronbach's Alpha", bah.attd.alpha_table),

data.frame(Measure = "Omega", bah.attd.omega_table)

)

# Create a kable table

bah.attd.kable_table <- bah.attd.combined_table %>%

kable("html", escape = FALSE, align = "c") %>%

kable_styling(full_width = FALSE)

bah.attd.kable_table

| **Measure** | **Estimate** | **SE** | **CI_Lower** | **CI_Upper** |
| --- | --- | --- | --- | --- |
| Cronbach’s Alpha | 0.9133827 | 0.1310712 | 0.8880117 | 0.9330059 |
| Omega | 0.9138026 | 0.0119197 | 0.8904405 | 0.9371648 |

2.6.2. Attachment Disturbance for the hoarding group

# Subset items for hoarding group

bah.attd.hd <- data3 %>%

filter(group == "HD") %>%

select(BAH.2, BAH.3, BAH.4, BAH.8, BAH.11, BAH.14, BAH.16, BAH.17, BAH.19, BAH.20, BAH.22, BAH.27)

## Cronbach's Alpha

bah.attd.hd.alpha_result <- ci.reliability(data = bah.attd.hd, type = "alpha", interval.type = "bonett")

bah.attd.hd.alpha_table <- data.frame(

Estimate = bah.attd.hd.alpha_result$est,

SE = bah.attd.hd.alpha_result$se,

CI_Lower = bah.attd.hd.alpha_result$ci.lower,

CI_Upper = bah.attd.hd.alpha_result$ci.upper

)

## Omega

bah.attd.hd.omega_result <- ci.reliability(data = bah.attd.hd, type = "omega", conf.level = 0.95)

bah.attd.hd.omega_table <- data.frame(

Estimate = bah.attd.hd.omega_result$est,

SE = bah.attd.hd.omega_result$se,

CI_Lower = bah.attd.hd.omega_result$ci.lower,

CI_Upper = bah.attd.hd.omega_result$ci.upper

)

# Combine results into a single table

bah.attd.hd.combined_table <- bind_rows(

data.frame(Measure = "Cronbach's Alpha", bah.attd.hd.alpha_table),

data.frame(Measure = "Omega", bah.attd.hd.omega_table)

)

# Create a kable table

bah.attd.hd.kable_table <- bah.attd.hd.combined_table %>%

kable("html", escape = FALSE, align = "c") %>%

kable_styling(full_width = FALSE)

bah.attd.hd.kable_table

| **Measure** | **Estimate** | **SE** | **CI_Lower** | **CI_Upper** |
| --- | --- | --- | --- | --- |
| Cronbach’s Alpha | 0.9231526 | 0.2306839 | 0.8792220 | 0.9511043 |
| Omega | 0.9256868 | 0.0167407 | 0.8928758 | 0.9584979 |

2.6.3. Attachment Disturbance for the hoarding and homelessness group

# Subset items for hoarding and homelessness group

bah.attd.h_and_h <- data3 %>%

filter(group == "H&H") %>%

select(BAH.2, BAH.3, BAH.4, BAH.8, BAH.11, BAH.14, BAH.16, BAH.17, BAH.19, BAH.20, BAH.22, BAH.27)

## Cronbach's Alpha

bah.attd.h_and_h.alpha_result <- ci.reliability(data = bah.attd.h_and_h, type = "alpha", interval.type = "bonett")

bah.attd.h_and_h.alpha_table <- data.frame(

Estimate = bah.attd.h_and_h.alpha_result$est,

SE = bah.attd.h_and_h.alpha_result$se,

CI_Lower = bah.attd.h_and_h.alpha_result$ci.lower,

CI_Upper = bah.attd.h_and_h.alpha_result$ci.upper

)

## Omega

bah.attd.h_and_h.omega_result <- ci.reliability(data = bah.attd.h_and_h, type = "omega", conf.level = 0.95)

bah.attd.h_and_h.omega_table <- data.frame(

Estimate = bah.attd.h_and_h.omega_result$est,

SE = bah.attd.h_and_h.omega_result$se,

CI_Lower = bah.attd.h_and_h.omega_result$ci.lower,

CI_Upper = bah.attd.h_and_h.omega_result$ci.upper

)

# Combine results into a single table

bah.attd.h_and_h.combined_table <- bind_rows(

data.frame(Measure = "Cronbach's Alpha", bah.attd.h_and_h.alpha_table),

data.frame(Measure = "Omega", bah.attd.h_and_h.omega_table)

)

# Create a kable table

bah.attd.h_and_h.kable_table <- bah.attd.h_and_h.combined_table %>%

kable("html", escape = FALSE, align = "c") %>%

kable_styling(full_width = FALSE)

bah.attd.h_and_h.kable_table

| **Measure** | **Estimate** | **SE** | **CI_Lower** | **CI_Upper** |
| --- | --- | --- | --- | --- |
| Cronbach’s Alpha | 0.8620982 | 0.2201928 | 0.7876763 | 0.9104343 |
| Omega | 0.8598786 | 0.0347829 | 0.7917054 | 0.9280519 |

2.6.4. Attachment Disturbance for the homelessness group

# Subset items for the homelessness group

bah.attd.hm <- data3 %>%

filter(group == "HM") %>%

select(BAH.2, BAH.3, BAH.4, BAH.8, BAH.11, BAH.14, BAH.16, BAH.17, BAH.19, BAH.20, BAH.22, BAH.27)

## Cronbach's Alpha

bah.attd.hm.alpha_result <- ci.reliability(data = bah.attd.hm, type = "alpha", interval.type = "bonett")

bah.attd.hm.alpha_table <- data.frame(

Estimate = bah.attd.hm.alpha_result$est,

SE = bah.attd.hm.alpha_result$se,

CI_Lower = bah.attd.hm.alpha_result$ci.lower,

CI_Upper = bah.attd.hm.alpha_result$ci.upper

)

## Omega

bah.attd.hm.omega_result <- ci.reliability(data = bah.attd.hm, type = "omega", conf.level = 0.95)

bah.attd.hm.omega_table <- data.frame(

Estimate = bah.attd.hm.omega_result$est,

SE = bah.attd.hm.omega_result$se,

CI_Lower = bah.attd.hm.omega_result$ci.lower,

CI_Upper = bah.attd.hm.omega_result$ci.upper

)

# Combine results into a single table

bah.attd.hm.combined_table <- bind_rows(

data.frame(Measure = "Cronbach's Alpha", bah.attd.hm.alpha_table),

data.frame(Measure = "Omega", bah.attd.hm.omega_table)

)

# Create a kable table

bah.attd.hm.kable_table <- bah.attd.hm.combined_table %>%

kable("html", escape = FALSE, align = "c") %>%

kable_styling(full_width = FALSE)

bah.attd.hm.kable_table

| **Measure** | **Estimate** | **SE** | **CI_Lower** | **CI_Upper** |
| --- | --- | --- | --- | --- |
| Cronbach’s Alpha | 0.8793453 | 0.2428334 | 0.8058022 | 0.9250375 |
| Omega | 0.8857350 | 0.0267219 | 0.8333610 | 0.9381090 |

*2.7. Savings Inventory-Revised: Clutter*

2.7.1. Clutter for the whole sample

# Subsetting subscale

sir.clt <- data3 %>% select(SIR.1, SIR.3, SIR.5, SIR.8, SIR.10, SIR.12, SIR.15, SIR.20, SIR.22)

## Cronbach's Alpha

sir.clt.alpha_result <- ci.reliability(data = sir.clt, type = "alpha", interval.type = "bonett")

sir.clt.alpha_table <- data.frame(

Estimate = sir.clt.alpha_result$est,

SE = sir.clt.alpha_result$se,

CI_Lower = sir.clt.alpha_result$ci.lower,

CI_Upper = sir.clt.alpha_result$ci.upper

)

## Omega

sir.clt.omega_result <- ci.reliability(data = sir.clt, type = "omega", conf.level = 0.95)

sir.clt.omega_table <- data.frame(

Estimate = sir.clt.omega_result$est,

SE = sir.clt.omega_result$se,

CI_Lower = sir.clt.omega_result$ci.lower,

CI_Upper = sir.clt.omega_result$ci.upper

)

# Combine results into a single table

sir.clt.combined_table <- bind_rows(

data.frame(Measure = "Cronbach's Alpha", sir.clt.alpha_table),

data.frame(Measure = "Omega", sir.clt.omega_table)

)

# Create a kable table

sir.clt.kable_table <- sir.clt.combined_table %>%

kable("html", escape = FALSE, align = "c") %>%

kable_styling(full_width = FALSE)

sir.clt.kable_table

| **Measure** | **Estimate** | **SE** | **CI_Lower** | **CI_Upper** |
| --- | --- | --- | --- | --- |
| Cronbach’s Alpha | 0.9391753 | 0.1331035 | 0.9210453 | 0.9531421 |
| Omega | 0.9440690 | 0.0073183 | 0.9297253 | 0.9584127 |

2.7.2. Clutter for the hoarding group

# Subset items for hoarding group

sir.clt.hd <- data3 %>%

filter(group == "HD") %>%

select(SIR.1, SIR.3, SIR.5, SIR.8, SIR.10, SIR.12, SIR.15, SIR.20, SIR.22)

## Cronbach's Alpha

sir.clt.hd.alpha_result <- ci.reliability(data = sir.clt.hd, type = "alpha", interval.type = "bonett")

sir.clt.hd.alpha_table <- data.frame(

Estimate = sir.clt.hd.alpha_result$est,

SE = sir.clt.hd.alpha_result$se,

CI_Lower = sir.clt.hd.alpha_result$ci.lower,

CI_Upper = sir.clt.hd.alpha_result$ci.upper

)

## Omega

sir.clt.hd.omega_result <- ci.reliability(data = sir.clt.hd, type = "omega", conf.level = 0.95)

sir.clt.hd.omega_table <- data.frame(

Estimate = sir.clt.hd.omega_result$est,

SE = sir.clt.hd.omega_result$se,

CI_Lower = sir.clt.hd.omega_result$ci.lower,

CI_Upper = sir.clt.hd.omega_result$ci.upper

)

# Combine results into a single table

sir.clt.hd.combined_table <- bind_rows(

data.frame(Measure = "Cronbach's Alpha", sir.clt.hd.alpha_table),

data.frame(Measure = "Omega", sir.clt.hd.omega_table)

)

# Create a kable table

sir.clt.hd.kable_table <- sir.clt.hd.combined_table %>%

kable("html", escape = FALSE, align = "c") %>%

kable_styling(full_width = FALSE)

sir.clt.hd.kable_table

| **Measure** | **Estimate** | **SE** | **CI_Lower** | **CI_Upper** |
| --- | --- | --- | --- | --- |
| Cronbach’s Alpha | 0.8951235 | 0.2342606 | 0.8340102 | 0.9337364 |
| Omega | 0.9044327 | 0.0197005 | 0.8658204 | 0.9430449 |

2.7.3. Clutter for the hoarding and homelessness group

# Subset items for hoarding and homelessness group

sir.clt.h_and_h <- data3 %>%

filter(group == "H&H") %>%

select(SIR.1, SIR.3, SIR.5, SIR.8, SIR.10, SIR.12, SIR.15, SIR.20, SIR.22)

## Cronbach's Alpha

sir.clt.h_and_h.alpha_result <- ci.reliability(data = sir.clt.h_and_h, type = "alpha", interval.type = "bonett")

sir.clt.h_and_h.alpha_table <- data.frame(

Estimate = sir.clt.h_and_h.alpha_result$est,

SE = sir.clt.h_and_h.alpha_result$se,

CI_Lower = sir.clt.h_and_h.alpha_result$ci.lower,

CI_Upper = sir.clt.h_and_h.alpha_result$ci.upper

)

## Omega

sir.clt.h_and_h.omega_result <- ci.reliability(data = sir.clt.h_and_h, type = "omega", conf.level = 0.95)

sir.clt.h_and_h.omega_table <- data.frame(

Estimate = sir.clt.h_and_h.omega_result$est,

SE = sir.clt.h_and_h.omega_result$se,

CI_Lower = sir.clt.h_and_h.omega_result$ci.lower,

CI_Upper = sir.clt.h_and_h.omega_result$ci.upper

)

# Combine results into a single table

sir.clt.h_and_h.combined_table <- bind_rows(

data.frame(Measure = "Cronbach's Alpha", sir.clt.h_and_h.alpha_table),

data.frame(Measure = "Omega", sir.clt.h_and_h.omega_table)

)

# Create a kable table

sir.clt.h_and_h.kable_table <- sir.clt.h_and_h.combined_table %>%

kable("html", escape = FALSE, align = "c") %>%

kable_styling(full_width = FALSE)

sir.clt.h_and_h.kable_table

| **Measure** | **Estimate** | **SE** | **CI_Lower** | **CI_Upper** |
| --- | --- | --- | --- | --- |
| Cronbach’s Alpha | 0.9055481 | 0.2236068 | 0.8535986 | 0.9390637 |
| Omega | 0.9114948 | 0.0205635 | 0.8711912 | 0.9517985 |

2.7.4. Clutter for the homelessness group

# Subset items for the homelessness group

sir.clt.hm <- data3 %>%

filter(group == "HM") %>%

select(SIR.1, SIR.3, SIR.5, SIR.8, SIR.10, SIR.12, SIR.15, SIR.20, SIR.22)

## Cronbach's Alpha

sir.clt.hm.alpha_result <- ci.reliability(data = sir.clt.hm, type = "alpha", interval.type = "bonett")

sir.clt.hm.alpha_table <- data.frame(

Estimate = sir.clt.hm.alpha_result$est,

SE = sir.clt.hm.alpha_result$se,

CI_Lower = sir.clt.hm.alpha_result$ci.lower,

CI_Upper = sir.clt.hm.alpha_result$ci.upper

)

## Omega

sir.clt.hm.omega_result <- ci.reliability(data = sir.clt.hm, type = "omega", conf.level = 0.95)

sir.clt.hm.omega_table <- data.frame(

Estimate = sir.clt.hm.omega_result$est,

SE = sir.clt.hm.omega_result$se,

CI_Lower = sir.clt.hm.omega_result$ci.lower,

CI_Upper = sir.clt.hm.omega_result$ci.upper

)

# Combine results into a single table

sir.clt.hm.combined_table <- bind_rows(

data.frame(Measure = "Cronbach's Alpha", sir.clt.hm.alpha_table),

data.frame(Measure = "Omega", sir.clt.hm.omega_table)

)

# Create a kable table

sir.clt.hm.kable_table <- sir.clt.hm.combined_table %>%

kable("html", escape = FALSE, align = "c") %>%

kable_styling(full_width = FALSE)

sir.clt.hm.kable_table

| **Measure** | **Estimate** | **SE** | **CI_Lower** | **CI_Upper** |
| --- | --- | --- | --- | --- |
| Cronbach’s Alpha | 0.8344963 | 0.2465985 | 0.7316431 | 0.8979289 |
| Omega | 0.8557857 | 0.0421072 | 0.7732571 | 0.9383142 |

*2.8. Savings Inventory-Revised: Difficulty Discarding*

2.8.1. Difficulty Discarding for the whole sample

# Subsetting subscale

sir.dd <- data3 %>% select(SIR.4, SIR.6, SIR.7, SIR.13, SIR.17, SIR.19, SIR.23)

## Cronbach's Alpha

sir.dd.alpha_result <- ci.reliability(data = sir.dd, type = "alpha", interval.type = "bonett")

sir.dd.alpha_table <- data.frame(

Estimate = sir.dd.alpha_result$est,

SE = sir.dd.alpha_result$se,

CI_Lower = sir.dd.alpha_result$ci.lower,

CI_Upper = sir.dd.alpha_result$ci.upper

)

## Omega

sir.dd.omega_result <- ci.reliability(data = sir.dd, type = "omega", conf.level = 0.95)

sir.dd.omega_table <- data.frame(

Estimate = sir.dd.omega_result$est,

SE = sir.dd.omega_result$se,

CI_Lower = sir.dd.omega_result$ci.lower,

CI_Upper = sir.dd.omega_result$ci.upper

)

# Combine results into a single table

sir.dd.combined_table <- bind_rows(

data.frame(Measure = "Cronbach's Alpha", sir.dd.alpha_table),

data.frame(Measure = "Omega", sir.dd.omega_table)

)

# Create a kable table

sir.dd.kable_table <- sir.dd.combined_table %>%

kable("html", escape = FALSE, align = "c") %>%

kable_styling(full_width = FALSE)

sir.dd.kable_table

| **Measure** | **Estimate** | **SE** | **CI_Lower** | **CI_Upper** |
| --- | --- | --- | --- | --- |
| Cronbach’s Alpha | 0.9032376 | 0.1355459 | 0.8737930 | 0.9258127 |
| Omega | 0.9080869 | 0.0145156 | 0.8796369 | 0.9365369 |

2.8.2. Difficulty Discarding for the hoarding group

# Subset items for hoarding group

sir.dd.hd <- data3 %>%

filter(group == "HD") %>%

select(SIR.4, SIR.6, SIR.7, SIR.13, SIR.17, SIR.19, SIR.23)

## Cronbach's Alpha

sir.dd.hd.alpha_result <- ci.reliability(data = sir.dd.hd, type = "alpha", interval.type = "bonett")

sir.dd.hd.alpha_table <- data.frame(

Estimate = sir.dd.hd.alpha_result$est,

SE = sir.dd.hd.alpha_result$se,

CI_Lower = sir.dd.hd.alpha_result$ci.lower,

CI_Upper = sir.dd.hd.alpha_result$ci.upper

)

## Omega

sir.dd.hd.omega_result <- ci.reliability(data = sir.dd.hd, type = "omega", conf.level = 0.95)

sir.dd.hd.omega_table <- data.frame(

Estimate = sir.dd.hd.omega_result$est,

SE = sir.dd.hd.omega_result$se,

CI_Lower = sir.dd.hd.omega_result$ci.lower,

CI_Upper = sir.dd.hd.omega_result$ci.upper

)

# Combine results into a single table

sir.dd.hd.combined_table <- bind_rows(

data.frame(Measure = "Cronbach's Alpha", sir.dd.hd.alpha_table),

data.frame(Measure = "Omega", sir.dd.hd.omega_table)

)

# Create a kable table

sir.dd.hd.kable_table <- sir.dd.hd.combined_table %>%

kable("html", escape = FALSE, align = "c") %>%

kable_styling(full_width = FALSE)

sir.dd.hd.kable_table

| **Measure** | **Estimate** | **SE** | **CI_Lower** | **CI_Upper** |
| --- | --- | --- | --- | --- |
| Cronbach’s Alpha | 0.7900632 | 0.2385594 | 0.6649183 | 0.8684695 |
| Omega | 0.7913873 | 0.0545737 | 0.6844247 | 0.8983499 |

2.8.3. Difficulty Discarding for the hoarding and homelessness group

# Subset items for hoarding and homelessness group

sir.dd.h_and_h <- data3 %>%

filter(group == "H&H") %>%

select(SIR.4, SIR.6, SIR.7, SIR.13, SIR.17, SIR.19, SIR.23)

## Cronbach's Alpha

sir.dd.h_and_h.alpha_result <- ci.reliability(data = sir.dd.h_and_h, type = "alpha", interval.type = "bonett")

sir.dd.h_and_h.alpha_table <- data.frame(

Estimate = sir.dd.h_and_h.alpha_result$est,

SE = sir.dd.h_and_h.alpha_result$se,

CI_Lower = sir.dd.h_and_h.alpha_result$ci.lower,

CI_Upper = sir.dd.h_and_h.alpha_result$ci.upper

)

## Omega

sir.dd.h_and_h.omega_result <- ci.reliability(data = sir.dd.h_and_h, type = "omega", conf.level = 0.95)

sir.dd.h_and_h.omega_table <- data.frame(

Estimate = sir.dd.h_and_h.omega_result$est,

SE = sir.dd.h_and_h.omega_result$se,

CI_Lower = sir.dd.h_and_h.omega_result$ci.lower,

CI_Upper = sir.dd.h_and_h.omega_result$ci.upper

)

# Combine results into a single table

sir.dd.h_and_h.combined_table <- bind_rows(

data.frame(Measure = "Cronbach's Alpha", sir.dd.h_and_h.alpha_table),

data.frame(Measure = "Omega", sir.dd.h_and_h.omega_table)

)

# Create a kable table

sir.dd.h_and_h.kable_table <- sir.dd.h_and_h.combined_table %>%

kable("html", escape = FALSE, align = "c") %>%

kable_styling(full_width = FALSE)

sir.dd.h_and_h.kable_table

| **Measure** | **Estimate** | **SE** | **CI_Lower** | **CI_Upper** |
| --- | --- | --- | --- | --- |
| Cronbach’s Alpha | 0.7903155 | 0.2277100 | 0.6723626 | 0.8658041 |
| Omega | 0.7650985 | 0.0697157 | 0.6284582 | 0.9017388 |

2.8.4. Difficulty Discarding for the homelessness group

# Subset items for the homelessness group

sir.dd.hm <- data3 %>%

filter(group == "HM") %>%

select(SIR.4, SIR.6, SIR.7, SIR.13, SIR.17, SIR.19, SIR.23)

## Cronbach's Alpha

sir.dd.hm.alpha_result <- ci.reliability(data = sir.dd.hm, type = "alpha", interval.type = "bonett")

sir.dd.hm.alpha_table <- data.frame(

Estimate = sir.dd.hm.alpha_result$est,

SE = sir.dd.hm.alpha_result$se,

CI_Lower = sir.dd.hm.alpha_result$ci.lower,

CI_Upper = sir.dd.hm.alpha_result$ci.upper

)

## Omega

sir.dd.hm.omega_result <- ci.reliability(data = sir.dd.hm, type = "omega", conf.level = 0.95)

sir.dd.hm.omega_table <- data.frame(

Estimate = sir.dd.hm.omega_result$est,

SE = sir.dd.hm.omega_result$se,

CI_Lower = sir.dd.hm.omega_result$ci.lower,

CI_Upper = sir.dd.hm.omega_result$ci.upper

)

# Combine results into a single table

sir.dd.hm.combined_table <- bind_rows(

data.frame(Measure = "Cronbach's Alpha", sir.dd.hm.alpha_table),

data.frame(Measure = "Omega", sir.dd.hm.omega_table)

)

# Create a kable table

sir.dd.hm.kable_table <- sir.dd.hm.combined_table %>%

kable("html", escape = FALSE, align = "c") %>%

kable_styling(full_width = FALSE)

sir.dd.hm.kable_table

| **Measure** | **Estimate** | **SE** | **CI_Lower** | **CI_Upper** |
| --- | --- | --- | --- | --- |
| Cronbach’s Alpha | 0.8456186 | 0.2511236 | 0.7474474 | 0.9056291 |
| Omega | 0.8696549 | 0.0267701 | 0.8171865 | 0.9221233 |

*2.9. Savings Inventory-Revised: Excessive Acquisition*

2.9.1. Excessive Acquisition for the whole sample

# Subsetting subscale

sir.exc <- data3 %>% select(SIR.2, SIR.9, SIR.11, SIR.14, SIR.16, SIR.18, SIR.21)

## Cronbach's Alpha

sir.exc.alpha_result <- ci.reliability(data = sir.exc, type = "alpha", interval.type = "bonett")

sir.exc.alpha_table <- data.frame(

Estimate = sir.exc.alpha_result$est,

SE = sir.exc.alpha_result$se,

CI_Lower = sir.exc.alpha_result$ci.lower,

CI_Upper = sir.exc.alpha_result$ci.upper

)

## Omega

sir.exc.omega_result <- ci.reliability(data = sir.exc, type = "omega", conf.level = 0.95)

sir.exc.omega_table <- data.frame(

Estimate = sir.exc.omega_result$est,

SE = sir.exc.omega_result$se,

CI_Lower = sir.exc.omega_result$ci.lower,

CI_Upper = sir.exc.omega_result$ci.upper

)

# Combine results into a single table

sir.exc.combined_table <- bind_rows(

data.frame(Measure = "Cronbach's Alpha", sir.exc.alpha_table),

data.frame(Measure = "Omega", sir.exc.omega_table)

)

# Create a kable table

sir.exc.kable_table <- sir.exc.combined_table %>%

kable("html", escape = FALSE, align = "c") %>%

kable_styling(full_width = FALSE)

sir.exc.kable_table

| **Measure** | **Estimate** | **SE** | **CI_Lower** | **CI_Upper** |
| --- | --- | --- | --- | --- |
| Cronbach’s Alpha | 0.7941007 | 0.1355459 | 0.7314459 | 0.8421379 |
| Omega | 0.8003079 | 0.0252959 | 0.7507288 | 0.8498870 |

2.9.2. Excessive Acquisition for the hoarding group

# Subset items for hoarding group

sir.exc.hd <- data3 %>%

filter(group == "HD") %>%

select(SIR.2, SIR.9, SIR.11, SIR.14, SIR.16, SIR.18, SIR.21)

## Cronbach's Alpha

sir.exc.hd.alpha_result <- ci.reliability(data = sir.exc.hd, type = "alpha", interval.type = "bonett")

sir.exc.hd.alpha_table <- data.frame(

Estimate = sir.exc.hd.alpha_result$est,

SE = sir.exc.hd.alpha_result$se,

CI_Lower = sir.exc.hd.alpha_result$ci.lower,

CI_Upper = sir.exc.hd.alpha_result$ci.upper

)

## Omega

sir.exc.hd.omega_result <- ci.reliability(data = sir.exc.hd, type = "omega", conf.level = 0.95)

sir.exc.hd.omega_table <- data.frame(

Estimate = sir.exc.hd.omega_result$est,

SE = sir.exc.hd.omega_result$se,

CI_Lower = sir.exc.hd.omega_result$ci.lower,

CI_Upper = sir.exc.hd.omega_result$ci.upper

)

# Combine results into a single table

sir.exc.hd.combined_table <- bind_rows(

data.frame(Measure = "Cronbach's Alpha", sir.exc.hd.alpha_table),

data.frame(Measure = "Omega", sir.exc.hd.omega_table)

)

# Create a kable table

sir.exc.hd.kable_table <- sir.exc.hd.combined_table %>%

kable("html", escape = FALSE, align = "c") %>%

kable_styling(full_width = FALSE)

sir.exc.hd.kable_table

| **Measure** | **Estimate** | **SE** | **CI_Lower** | **CI_Upper** |
| --- | --- | --- | --- | --- |
| Cronbach’s Alpha | 0.7990351 | 0.2385594 | 0.6792384 | 0.8740906 |
| Omega | 0.8023615 | 0.0513221 | 0.7017720 | 0.9029509 |

2.9.3. Excessive Acquisition for the hoarding and homelessness group

# Subset items for hoarding and homelessness group

sir.exc.h_and_h <- data3 %>%

filter(group == "H&H") %>%

select(SIR.2, SIR.9, SIR.11, SIR.14, SIR.16, SIR.18, SIR.21)

## Cronbach's Alpha

sir.exc.h_and_h.alpha_result <- ci.reliability(data = sir.exc.h_and_h, type = "alpha", interval.type = "bonett")

sir.exc.h_and_h.alpha_table <- data.frame(

Estimate = sir.exc.h_and_h.alpha_result$est,

SE = sir.exc.h_and_h.alpha_result$se,

CI_Lower = sir.exc.h_and_h.alpha_result$ci.lower,

CI_Upper = sir.exc.h_and_h.alpha_result$ci.upper

)

## Omega

sir.exc.h_and_h.omega_result <- ci.reliability(data = sir.exc.h_and_h, type = "omega", conf.level = 0.95)

sir.exc.h_and_h.omega_table <- data.frame(

Estimate = sir.exc.h_and_h.omega_result$est,

SE = sir.exc.h_and_h.omega_result$se,

CI_Lower = sir.exc.h_and_h.omega_result$ci.lower,

CI_Upper = sir.exc.h_and_h.omega_result$ci.upper

)

# Combine results into a single table

sir.exc.h_and_h.combined_table <- bind_rows(

data.frame(Measure = "Cronbach's Alpha", sir.exc.h_and_h.alpha_table),

data.frame(Measure = "Omega", sir.exc.h_and_h.omega_table)

)

# Create a kable table

sir.exc.h_and_h.kable_table <- sir.exc.h_and_h.combined_table %>%

kable("html", escape = FALSE, align = "c") %>%

kable_styling(full_width = FALSE)

sir.exc.h_and_h.kable_table

| **Measure** | **Estimate** | **SE** | **CI_Lower** | **CI_Upper** |
| --- | --- | --- | --- | --- |
| Cronbach’s Alpha | 0.6979820 | 0.2277100 | 0.5280891 | 0.8067116 |
| Omega | 0.7226079 | 0.0560286 | 0.6127939 | 0.8324219 |

2.9.4. Excessive Acquisition for the homelessness group

# Subset items for the homelessness group

sir.exc.hm <- data3 %>%

filter(group == "HM") %>%

select(SIR.2, SIR.9, SIR.11, SIR.14, SIR.16, SIR.18, SIR.21)

## Cronbach's Alpha

sir.exc.hm.alpha_result <- ci.reliability(data = sir.exc.hm, type = "alpha", interval.type = "bonett")

sir.exc.hm.alpha_table <- data.frame(

Estimate = sir.exc.hm.alpha_result$est,

SE = sir.exc.hm.alpha_result$se,

CI_Lower = sir.exc.hm.alpha_result$ci.lower,

CI_Upper = sir.exc.hm.alpha_result$ci.upper

)

## Omega

sir.exc.hm.omega_result <- ci.reliability(data = sir.exc.hm, type = "omega", conf.level = 0.95)

sir.exc.hm.omega_table <- data.frame(

Estimate = sir.exc.hm.omega_result$est,

SE = sir.exc.hm.omega_result$se,

CI_Lower = sir.exc.hm.omega_result$ci.lower,

CI_Upper = sir.exc.hm.omega_result$ci.upper

)

# Combine results into a single table

sir.exc.hm.combined_table <- bind_rows(

data.frame(Measure = "Cronbach's Alpha", sir.exc.hm.alpha_table),

data.frame(Measure = "Omega", sir.exc.hm.omega_table)

)

# Create a kable table

sir.exc.hm.kable_table <- sir.exc.hm.combined_table %>%

kable("html", escape = FALSE, align = "c") %>%

kable_styling(full_width = FALSE)

sir.exc.hm.kable_table

| **Measure** | **Estimate** | **SE** | **CI_Lower** | **CI_Upper** |
| --- | --- | --- | --- | --- |
| Cronbach’s Alpha | 0.7215333 | 0.2511236 | 0.5444562 | 0.8297777 |
| Omega | 0.7408890 | 0.0469004 | 0.6489659 | 0.8328121 |

*2.10. Interpersonal Needs Questionnaire: Perceived Burdensomeness*

2.10.1. Perceived Burdensomeness for the whole sample

# Subsetting subscale

inq.pb <- data3 %>% select(INQ.1, INQ.2, INQ.3, INQ.4, INQ.5, INQ.6)

## Cronbach's Alpha

inq.pb.alpha_result <- ci.reliability(data = inq.pb, type = "alpha", interval.type = "bonett")

inq.pb.alpha_table <- data.frame(

Estimate = inq.pb.alpha_result$est,

SE = inq.pb.alpha_result$se,

CI_Lower = inq.pb.alpha_result$ci.lower,

CI_Upper = inq.pb.alpha_result$ci.upper

)

## Omega

inq.pb.omega_result <- ci.reliability(data = inq.pb, type = "omega", conf.level = 0.95)

inq.pb.omega_table <- data.frame(

Estimate = inq.pb.omega_result$est,

SE = inq.pb.omega_result$se,

CI_Lower = inq.pb.omega_result$ci.lower,

CI_Upper = inq.pb.omega_result$ci.upper

)

# Combine results into a single table

inq.pb.combined_table <- bind_rows(

data.frame(Measure = "Cronbach's Alpha", inq.pb.alpha_table),

data.frame(Measure = "Omega", inq.pb.omega_table)

)

# Create a kable table

inq.pb.kable_table <- inq.pb.combined_table %>%

kable("html", escape = FALSE, align = "c") %>%

kable_styling(full_width = FALSE)

inq.pb.kable_table

| **Measure** | **Estimate** | **SE** | **CI_Lower** | **CI_Upper** |
| --- | --- | --- | --- | --- |
| Cronbach’s Alpha | 0.9102030 | 0.1374687 | 0.8824358 | 0.9314120 |
| Omega | 0.9121427 | 0.0144537 | 0.8838141 | 0.9404714 |

2.10.2. Perceived Burdensomeness for the hoarding group

# Subset items for hoarding group

inq.pb.hd <- data3 %>%

filter(group == "HD") %>%

select(INQ.1, INQ.2, INQ.3, INQ.4, INQ.5, INQ.6)

## Cronbach's Alpha

inq.pb.hd.alpha_result <- ci.reliability(data = inq.pb.hd, type = "alpha", interval.type = "bonett")

inq.pb.hd.alpha_table <- data.frame(

Estimate = inq.pb.hd.alpha_result$est,

SE = inq.pb.hd.alpha_result$se,

CI_Lower = inq.pb.hd.alpha_result$ci.lower,

CI_Upper = inq.pb.hd.alpha_result$ci.upper

)

## Omega

inq.pb.hd.omega_result <- ci.reliability(data = inq.pb.hd, type = "omega", conf.level = 0.95)

inq.pb.hd.omega_table <- data.frame(

Estimate = inq.pb.hd.omega_result$est,

SE = inq.pb.hd.omega_result$se,

CI_Lower = inq.pb.hd.omega_result$ci.lower,

CI_Upper = inq.pb.hd.omega_result$ci.upper

)

# Combine results into a single table

inq.pb.hd.combined_table <- bind_rows(

data.frame(Measure = "Cronbach's Alpha", inq.pb.hd.alpha_table),

data.frame(Measure = "Omega", inq.pb.hd.omega_table)

)

# Create a kable table

inq.pb.hd.kable_table <- inq.pb.hd.combined_table %>%

kable("html", escape = FALSE, align = "c") %>%

kable_styling(full_width = FALSE)

inq.pb.hd.kable_table

| **Measure** | **Estimate** | **SE** | **CI_Lower** | **CI_Upper** |
| --- | --- | --- | --- | --- |
| Cronbach’s Alpha | 0.9059361 | 0.2419434 | 0.8488648 | 0.9414562 |
| Omega | 0.9073223 | 0.0248985 | 0.8585221 | 0.9561225 |

2.10.3. Perceived Burdensomeness for the hoarding and homelessness group

# Subset items for hoarding and homelessness group

inq.pb.h_and_h <- data3 %>%

filter(group == "H&H") %>%

select(INQ.1, INQ.2, INQ.3, INQ.4, INQ.5, INQ.6)

## Cronbach's Alpha

inq.pb.h_and_h.alpha_result <- ci.reliability(data = inq.pb.h_and_h, type = "alpha", interval.type = "bonett")

inq.pb.h_and_h.alpha_table <- data.frame(

Estimate = inq.pb.h_and_h.alpha_result$est,

SE = inq.pb.h_and_h.alpha_result$se,

CI_Lower = inq.pb.h_and_h.alpha_result$ci.lower,

CI_Upper = inq.pb.h_and_h.alpha_result$ci.upper

)

## Omega

inq.pb.h_and_h.omega_result <- ci.reliability(data = inq.pb.h_and_h, type = "omega", conf.level = 0.95)

inq.pb.h_and_h.omega_table <- data.frame(

Estimate = inq.pb.h_and_h.omega_result$est,

SE = inq.pb.h_and_h.omega_result$se,

CI_Lower = inq.pb.h_and_h.omega_result$ci.lower,

CI_Upper = inq.pb.h_and_h.omega_result$ci.upper

)

# Combine results into a single table

inq.pb.h_and_h.combined_table <- bind_rows(

data.frame(Measure = "Cronbach's Alpha", inq.pb.h_and_h.alpha_table),

data.frame(Measure = "Omega", inq.pb.h_and_h.omega_table)

)

# Create a kable table

inq.pb.h_and_h.kable_table <- inq.pb.h_and_h.combined_table %>%

kable("html", escape = FALSE, align = "c") %>%

kable_styling(full_width = FALSE)

inq.pb.h_and_h.kable_table

| **Measure** | **Estimate** | **SE** | **CI_Lower** | **CI_Upper** |
| --- | --- | --- | --- | --- |
| Cronbach’s Alpha | 0.9107327 | 0.2309401 | 0.8596317 | 0.9432304 |
| Omega | 0.9141346 | 0.0207255 | 0.8735135 | 0.9547558 |

2.10.4. Perceived Burdensomeness for the homelessness group

# Subset items for the homelessness group

inq.pb.hm <- data3 %>%

filter(group == "HM") %>%

select(INQ.1, INQ.2, INQ.3, INQ.4, INQ.5, INQ.6)

## Cronbach's Alpha

inq.pb.hm.alpha_result <- ci.reliability(data = inq.pb.hm, type = "alpha", interval.type = "bonett")

inq.pb.hm.alpha_table <- data.frame(

Estimate = inq.pb.hm.alpha_result$est,

SE = inq.pb.hm.alpha_result$se,

CI_Lower = inq.pb.hm.alpha_result$ci.lower,

CI_Upper = inq.pb.hm.alpha_result$ci.upper

)

## Omega

inq.pb.hm.omega_result <- ci.reliability(data = inq.pb.hm, type = "omega", conf.level = 0.95)

inq.pb.hm.omega_table <- data.frame(

Estimate = inq.pb.hm.omega_result$est,

SE = inq.pb.hm.omega_result$se,

CI_Lower = inq.pb.hm.omega_result$ci.lower,

CI_Upper = inq.pb.hm.omega_result$ci.upper

)

# Combine results into a single table

inq.pb.hm.combined_table <- bind_rows(

data.frame(Measure = "Cronbach's Alpha", inq.pb.hm.alpha_table),

data.frame(Measure = "Omega", inq.pb.hm.omega_table)

)

# Create a kable table

inq.pb.hm.kable_table <- inq.pb.hm.combined_table %>%

kable("html", escape = FALSE, align = "c") %>%

kable_styling(full_width = FALSE)

inq.pb.hm.kable_table

| **Measure** | **Estimate** | **SE** | **CI_Lower** | **CI_Upper** |
| --- | --- | --- | --- | --- |
| Cronbach’s Alpha | 0.9031021 | 0.2546858 | 0.8403741 | 0.9411799 |
| Omega | 0.9063096 | 0.0356747 | 0.8363884 | 0.9762308 |

*2.11. Interpersonal Needs Questionnaire: Thwarted Belongingness*

2.11.1. Thwarted Belongingness for the whole sample

# Subsetting subscale

inq.tbl <- data3 %>% select(INQ.7, INQ.8, INQ.9, INQ.10, INQ.11, INQ.12, INQ.13, INQ.14, INQ.15)

## Cronbach's Alpha

inq.tbl.alpha_result <- ci.reliability(data = inq.tbl, type = "alpha", interval.type = "bonett")

inq.tbl.alpha_table <- data.frame(

Estimate = inq.tbl.alpha_result$est,

SE = inq.tbl.alpha_result$se,

CI_Lower = inq.tbl.alpha_result$ci.lower,

CI_Upper = inq.tbl.alpha_result$ci.upper

)

## Omega

inq.tbl.omega_result <- ci.reliability(data = inq.tbl, type = "omega", conf.level = 0.95)

inq.tbl.omega_table <- data.frame(

Estimate = inq.tbl.omega_result$est,

SE = inq.tbl.omega_result$se,

CI_Lower = inq.tbl.omega_result$ci.lower,

CI_Upper = inq.tbl.omega_result$ci.upper

)

# Combine results into a single table

inq.tbl.combined_table <- bind_rows(

data.frame(Measure = "Cronbach's Alpha", inq.tbl.alpha_table),

data.frame(Measure = "Omega", inq.tbl.omega_table)

)

# Create a kable table

inq.tbl.kable_table <- inq.tbl.combined_table %>%

kable("html", escape = FALSE, align = "c") %>%

kable_styling(full_width = FALSE)

inq.tbl.kable_table

| **Measure** | **Estimate** | **SE** | **CI_Lower** | **CI_Upper** |
| --- | --- | --- | --- | --- |
| Cronbach’s Alpha | 0.8472471 | 0.1331035 | 0.8017162 | 0.8823230 |
| Omega | 0.8507645 | 0.0221001 | 0.8074492 | 0.8940798 |

2.11.2. Thwarted Belongingness for the hoarding group

# Subset items for hoarding group

inq.tbl.hd <- data3 %>%

filter(group == "HD") %>%

select(INQ.7, INQ.8, INQ.9, INQ.10, INQ.11, INQ.12, INQ.13, INQ.14, INQ.15)

## Cronbach's Alpha

inq.tbl.hd.alpha_result <- ci.reliability(data = inq.tbl.hd, type = "alpha", interval.type = "bonett")

inq.tbl.hd.alpha_table <- data.frame(

Estimate = inq.tbl.hd.alpha_result$est,

SE = inq.tbl.hd.alpha_result$se,

CI_Lower = inq.tbl.hd.alpha_result$ci.lower,

CI_Upper = inq.tbl.hd.alpha_result$ci.upper

)

## Omega

inq.tbl.hd.omega_result <- ci.reliability(data = inq.tbl.hd, type = "omega", conf.level = 0.95)

inq.tbl.hd.omega_table <- data.frame(

Estimate = inq.tbl.hd.omega_result$est,

SE = inq.tbl.hd.omega_result$se,

CI_Lower = inq.tbl.hd.omega_result$ci.lower,

CI_Upper = inq.tbl.hd.omega_result$ci.upper

)

# Combine results into a single table

inq.tbl.hd.combined_table <- bind_rows(

data.frame(Measure = "Cronbach's Alpha", inq.tbl.hd.alpha_table),

data.frame(Measure = "Omega", inq.tbl.hd.omega_table)

)

# Create a kable table

inq.tbl.hd.kable_table <- inq.tbl.hd.combined_table %>%

kable("html", escape = FALSE, align = "c") %>%

kable_styling(full_width = FALSE)

inq.tbl.hd.kable_table

| **Measure** | **Estimate** | **SE** | **CI_Lower** | **CI_Upper** |
| --- | --- | --- | --- | --- |
| Cronbach’s Alpha | 0.9148515 | 0.2342606 | 0.8652341 | 0.9462010 |
| Omega | 0.9179588 | 0.0211501 | 0.8765055 | 0.9594122 |

2.11.3. Thwarted Belongingness for the hoarding and homelessness group

# Subset items for hoarding and homelessness group

inq.tbl.h_and_h <- data3 %>%

filter(group == "H&H") %>%

select(INQ.7, INQ.8, INQ.9, INQ.10, INQ.11, INQ.12, INQ.13, INQ.14, INQ.15)

## Cronbach's Alpha

inq.tbl.h_and_h.alpha_result <- ci.reliability(data = inq.tbl.h_and_h, type = "alpha", interval.type = "bonett")

inq.tbl.h_and_h.alpha_table <- data.frame(

Estimate = inq.tbl.h_and_h.alpha_result$est,

SE = inq.tbl.h_and_h.alpha_result$se,

CI_Lower = inq.tbl.h_and_h.alpha_result$ci.lower,

CI_Upper = inq.tbl.h_and_h.alpha_result$ci.upper

)

## Omega

inq.tbl.h_and_h.omega_result <- ci.reliability(data = inq.tbl.h_and_h, type = "omega", conf.level = 0.95)

inq.tbl.h_and_h.omega_table <- data.frame(

Estimate = inq.tbl.h_and_h.omega_result$est,

SE = inq.tbl.h_and_h.omega_result$se,

CI_Lower = inq.tbl.h_and_h.omega_result$ci.lower,

CI_Upper = inq.tbl.h_and_h.omega_result$ci.upper

)

# Combine results into a single table

inq.tbl.h_and_h.combined_table <- bind_rows(

data.frame(Measure = "Cronbach's Alpha", inq.tbl.h_and_h.alpha_table),

data.frame(Measure = "Omega", inq.tbl.h_and_h.omega_table)

)

# Create a kable table

inq.tbl.h_and_h.kable_table <- inq.tbl.h_and_h.combined_table %>%

kable("html", escape = FALSE, align = "c") %>%

kable_styling(full_width = FALSE)

inq.tbl.h_and_h.kable_table

| **Measure** | **Estimate** | **SE** | **CI_Lower** | **CI_Upper** |
| --- | --- | --- | --- | --- |
| Cronbach’s Alpha | 0.8075605 | 0.2236068 | 0.7017169 | 0.8758463 |
| Omega | 0.8152583 | 0.0401032 | 0.7366575 | 0.8938591 |

2.11.4. Thwarted Belongingness for the homelessness group

# Subset items for the homelessness group

inq.tbl.hm <- data3 %>%

filter(group == "HM") %>%

select(INQ.7, INQ.8, INQ.9, INQ.10, INQ.11, INQ.12, INQ.13, INQ.14, INQ.15)

## Cronbach's Alpha

inq.tbl.hm.alpha_result <- ci.reliability(data = inq.tbl.hm, type = "alpha", interval.type = "bonett")

inq.tbl.hm.alpha_table <- data.frame(

Estimate = inq.tbl.hm.alpha_result$est,

SE = inq.tbl.hm.alpha_result$se,

CI_Lower = inq.tbl.hm.alpha_result$ci.lower,

CI_Upper = inq.tbl.hm.alpha_result$ci.upper

)

## Omega

inq.tbl.hm.omega_result <- ci.reliability(data = inq.tbl.hm, type = "omega", conf.level = 0.95)

inq.tbl.hm.omega_table <- data.frame(

Estimate = inq.tbl.hm.omega_result$est,

SE = inq.tbl.hm.omega_result$se,

CI_Lower = inq.tbl.hm.omega_result$ci.lower,

CI_Upper = inq.tbl.hm.omega_result$ci.upper

)

# Combine results into a single table

inq.tbl.hm.combined_table <- bind_rows(

data.frame(Measure = "Cronbach's Alpha", inq.tbl.hm.alpha_table),

data.frame(Measure = "Omega", inq.tbl.hm.omega_table)

)

# Create a kable table

inq.tbl.hm.kable_table <- inq.tbl.hm.combined_table %>%

kable("html", escape = FALSE, align = "c") %>%

kable_styling(full_width = FALSE)

inq.tbl.hm.kable_table

| **Measure** | **Estimate** | **SE** | **CI_Lower** | **CI_Upper** |
| --- | --- | --- | --- | --- |
| Cronbach’s Alpha | 0.8052977 | 0.2465985 | 0.6842989 | 0.8799213 |
| Omega | 0.8242545 | 0.0420423 | 0.7418530 | 0.9066559 |

*2.12. Experiences of Early Material Deprivation Questionnaire*

2.12.1. Experiences of Early Material Deprivation Questionnaire for the whole sample

# Subsetting subscale

eemd <- data3 %>% select(EEMDQ.1, EEMDQ.2, EEMDQ.3, EEMDQ.4, EEMDQ.5, EEMDQ.6, EEMDQ.7, EEMDQ.8, EEMDQ.9, EEMDQ.10, EEMDQ.11)

## Cronbach's Alpha

eemd.alpha_result <- ci.reliability(data = eemd, type = "alpha", interval.type = "bonett")

eemd.alpha_table <- data.frame(

Estimate = eemd.alpha_result$est,

SE = eemd.alpha_result$se,

CI_Lower = eemd.alpha_result$ci.lower,

CI_Upper = eemd.alpha_result$ci.upper

)

## Omega

eemd.omega_result <- ci.reliability(data = eemd, type = "omega", conf.level = 0.95)

eemd.omega_table <- data.frame(

Estimate = eemd.omega_result$est,

SE = eemd.omega_result$se,

CI_Lower = eemd.omega_result$ci.lower,

CI_Upper = eemd.omega_result$ci.upper

)

# Combine results into a single table

eemd.combined_table <- bind_rows(

data.frame(Measure = "Cronbach's Alpha", eemd.alpha_table),

data.frame(Measure = "Omega", eemd.omega_table)

)

# Create a kable table

eemd.kable_table <- eemd.combined_table %>%

kable("html", escape = FALSE, align = "c") %>%

kable_styling(full_width = FALSE)

eemd.kable_table

| **Measure** | **Estimate** | **SE** | **CI_Lower** | **CI_Upper** |
| --- | --- | --- | --- | --- |
| Cronbach’s Alpha | 0.9375754 | 0.1316162 | 0.9192044 | 0.9517693 |
| Omega | 0.9375030 | 0.0099777 | 0.9179471 | 0.9570589 |

2.12.2. Experiences of Early Material Deprivation Questionnaire for the hoarding group

# Subset items for hoarding group

eemd.hd <- data3 %>%

filter(group == "HD") %>%

select(EEMDQ.1, EEMDQ.2, EEMDQ.3, EEMDQ.4, EEMDQ.5, EEMDQ.6, EEMDQ.7, EEMDQ.8, EEMDQ.9, EEMDQ.10, EEMDQ.11)

## Cronbach's Alpha

eemd.hd.alpha_result <- ci.reliability(data = eemd.hd, type = "alpha", interval.type = "bonett")

eemd.hd.alpha_table <- data.frame(

Estimate = eemd.hd.alpha_result$est,

SE = eemd.hd.alpha_result$se,

CI_Lower = eemd.hd.alpha_result$ci.lower,

CI_Upper = eemd.hd.alpha_result$ci.upper

)

## Omega

eemd.hd.omega_result <- ci.reliability(data = eemd.hd, type = "omega", conf.level = 0.95)

eemd.hd.omega_table <- data.frame(

Estimate = eemd.hd.omega_result$est,

SE = eemd.hd.omega_result$se,

CI_Lower = eemd.hd.omega_result$ci.lower,

CI_Upper = eemd.hd.omega_result$ci.upper

)

# Combine results into a single table

eemd.hd.combined_table <- bind_rows(

data.frame(Measure = "Cronbach's Alpha", eemd.hd.alpha_table),

data.frame(Measure = "Omega", eemd.hd.omega_table)

)

# Create a kable table

eemd.hd.kable_table <- eemd.hd.combined_table %>%

kable("html", escape = FALSE, align = "c") %>%

kable_styling(full_width = FALSE)

eemd.hd.kable_table

| **Measure** | **Estimate** | **SE** | **CI_Lower** | **CI_Upper** |
| --- | --- | --- | --- | --- |
| Cronbach’s Alpha | 0.8766695 | 0.2316431 | 0.8058017 | 0.9216759 |
| Omega | 0.8836928 | 0.0318530 | 0.8212621 | 0.9461235 |

2.12.3. Experiences of Early Material Deprivation Questionnaire for the hoarding and homelessness group

# Subset items for hoarding and homelessness group

eemd.h_and_h <- data3 %>%

filter(group == "H&H") %>%

select(EEMDQ.1, EEMDQ.2, EEMDQ.3, EEMDQ.4, EEMDQ.5, EEMDQ.6, EEMDQ.7, EEMDQ.8, EEMDQ.9, EEMDQ.10, EEMDQ.11)

## Cronbach's Alpha

eemd.h_and_h.alpha_result <- ci.reliability(data = eemd.h_and_h, type = "alpha", interval.type = "bonett")

eemd.h_and_h.alpha_table <- data.frame(

Estimate = eemd.h_and_h.alpha_result$est,

SE = eemd.h_and_h.alpha_result$se,

CI_Lower = eemd.h_and_h.alpha_result$ci.lower,

CI_Upper = eemd.h_and_h.alpha_result$ci.upper

)

## Omega

eemd.h_and_h.omega_result <- ci.reliability(data = eemd.h_and_h, type = "omega", conf.level = 0.95)

eemd.h_and_h.omega_table <- data.frame(

Estimate = eemd.h_and_h.omega_result$est,

SE = eemd.h_and_h.omega_result$se,

CI_Lower = eemd.h_and_h.omega_result$ci.lower,

CI_Upper = eemd.h_and_h.omega_result$ci.upper

)

# Combine results into a single table

eemd.h_and_h.combined_table <- bind_rows(

data.frame(Measure = "Cronbach's Alpha", eemd.h_and_h.alpha_table),

data.frame(Measure = "Omega", eemd.h_and_h.omega_table)

)

# Create a kable table

eemd.h_and_h.kable_table <- eemd.h_and_h.combined_table %>%

kable("html", escape = FALSE, align = "c") %>%

kable_styling(full_width = FALSE)

eemd.h_and_h.kable_table

| **Measure** | **Estimate** | **SE** | **CI_Lower** | **CI_Upper** |
| --- | --- | --- | --- | --- |
| Cronbach’s Alpha | 0.9495611 | 0.2211083 | 0.9222012 | 0.9672993 |
| Omega | 0.9495716 | 0.0118435 | 0.9263588 | 0.9727844 |

2.12.4. Experiences of Early Material Deprivation Questionnaire for the homelessness group

# Subset items for the homelessness group

eemd.hm <- data3 %>%

filter(group == "HM") %>%

select(EEMDQ.1, EEMDQ.2, EEMDQ.3, EEMDQ.4, EEMDQ.5, EEMDQ.6, EEMDQ.7, EEMDQ.8, EEMDQ.9, EEMDQ.10, EEMDQ.11)

## Cronbach's Alpha

eemd.hm.alpha_result <- ci.reliability(data = eemd.hm, type = "alpha", interval.type = "bonett")

eemd.hm.alpha_table <- data.frame(

Estimate = eemd.hm.alpha_result$est,

SE = eemd.hm.alpha_result$se,

CI_Lower = eemd.hm.alpha_result$ci.lower,

CI_Upper = eemd.hm.alpha_result$ci.upper

)

## Omega

eemd.hm.omega_result <- ci.reliability(data = eemd.hm, type = "omega", conf.level = 0.95)

eemd.hm.omega_table <- data.frame(

Estimate = eemd.hm.omega_result$est,

SE = eemd.hm.omega_result$se,

CI_Lower = eemd.hm.omega_result$ci.lower,

CI_Upper = eemd.hm.omega_result$ci.upper

)

# Combine results into a single table

eemd.hm.combined_table <- bind_rows(

data.frame(Measure = "Cronbach's Alpha", eemd.hm.alpha_table),

data.frame(Measure = "Omega", eemd.hm.omega_table)

)

# Create a kable table

eemd.hm.kable_table <- eemd.hm.combined_table %>%

kable("html", escape = FALSE, align = "c") %>%

kable_styling(full_width = FALSE)

eemd.hm.kable_table

| **Measure** | **Estimate** | **SE** | **CI_Lower** | **CI_Upper** |
| --- | --- | --- | --- | --- |
| Cronbach’s Alpha | 0.9302225 | 0.2438431 | 0.8874683 | 0.9567331 |
| Omega | 0.9274533 | 0.0230368 | 0.8823019 | 0.9726047 |

*2.13. Material Deprivation and Hoarding Beliefs*

2.13.1. Material Deprivation and Hoarding Beliefs for the whole sample

# Subsetting subscale

mdhb <- data3 %>% select(MDHB.1:MDHB.9)

## Cronbach's Alpha

mdhb.alpha_result <- ci.reliability(data = mdhb, type = "alpha", interval.type = "bonett")

mdhb.alpha_table <- data.frame(

Estimate = mdhb.alpha_result$est,

SE = mdhb.alpha_result$se,

CI_Lower = mdhb.alpha_result$ci.lower,

CI_Upper = mdhb.alpha_result$ci.upper

)

## Omega

mdhb.omega_result <- ci.reliability(data = mdhb, type = "omega", conf.level = 0.95)

mdhb.omega_table <- data.frame(

Estimate = mdhb.omega_result$est,

SE = mdhb.omega_result$se,

CI_Lower = mdhb.omega_result$ci.lower,

CI_Upper = mdhb.omega_result$ci.upper

)

# Combine results into a single table

mdhb.combined_table <- bind_rows(

data.frame(Measure = "Cronbach's Alpha", mdhb.alpha_table),

data.frame(Measure = "Omega", mdhb.omega_table)

)

# Create a kable table

mdhb.kable_table <- mdhb.combined_table %>%

kable("html", escape = FALSE, align = "c") %>%

kable_styling(full_width = FALSE)

mdhb.kable_table

| **Measure** | **Estimate** | **SE** | **CI_Lower** | **CI_Upper** |
| --- | --- | --- | --- | --- |
| Cronbach’s Alpha | 0.9232221 | 0.1331035 | 0.9003369 | 0.9408522 |
| Omega | 0.9246847 | 0.0105777 | 0.9039529 | 0.9454165 |

2.13.2. Material Deprivation and Hoarding Beliefs for the hoarding group

# Subset items for hoarding group

mdhb.hd <- data3 %>%

filter(group == "HD") %>%

select(MDHB.1:MDHB.9)

## Cronbach's Alpha

mdhb.hd.alpha_result <- ci.reliability(data = mdhb.hd, type = "alpha", interval.type = "bonett")

mdhb.hd.alpha_table <- data.frame(

Estimate = mdhb.hd.alpha_result$est,

SE = mdhb.hd.alpha_result$se,

CI_Lower = mdhb.hd.alpha_result$ci.lower,

CI_Upper = mdhb.hd.alpha_result$ci.upper

)

## Omega

mdhb.hd.omega_result <- ci.reliability(data = mdhb.hd, type = "omega", conf.level = 0.95)

mdhb.hd.omega_table <- data.frame(

Estimate = mdhb.hd.omega_result$est,

SE = mdhb.hd.omega_result$se,

CI_Lower = mdhb.hd.omega_result$ci.lower,

CI_Upper = mdhb.hd.omega_result$ci.upper

)

# Combine results into a single table

mdhb.hd.combined_table <- bind_rows(

data.frame(Measure = "Cronbach's Alpha", mdhb.hd.alpha_table),

data.frame(Measure = "Omega", mdhb.hd.omega_table)

)

# Create a kable table

mdhb.hd.kable_table <- mdhb.hd.combined_table %>%

kable("html", escape = FALSE, align = "c") %>%

kable_styling(full_width = FALSE)

mdhb.hd.kable_table

| **Measure** | **Estimate** | **SE** | **CI_Lower** | **CI_Upper** |
| --- | --- | --- | --- | --- |
| Cronbach’s Alpha | 0.9214829 | 0.2342606 | 0.8757298 | 0.9503909 |
| Omega | 0.9235333 | 0.0183058 | 0.8876547 | 0.9594120 |

2.13.3. Material Deprivation and Hoarding Beliefs for the hoarding and homelessness group

# Subset items for hoarding and homelessness group

mdhb.h_and_h <- data3 %>%

filter(group == "H&H") %>%

select(MDHB.1:MDHB.9)

## Cronbach's Alpha

mdhb.h_and_h.alpha_result <- ci.reliability(data = mdhb.h_and_h, type = "alpha", interval.type = "bonett")

mdhb.h_and_h.alpha_table <- data.frame(

Estimate = mdhb.h_and_h.alpha_result$est,

SE = mdhb.h_and_h.alpha_result$se,

CI_Lower = mdhb.h_and_h.alpha_result$ci.lower,

CI_Upper = mdhb.h_and_h.alpha_result$ci.upper

)

## Omega

mdhb.h_and_h.omega_result <- ci.reliability(data = mdhb.h_and_h, type = "omega", conf.level = 0.95)

mdhb.h_and_h.omega_table <- data.frame(

Estimate = mdhb.h_and_h.omega_result$est,

SE = mdhb.h_and_h.omega_result$se,

CI_Lower = mdhb.h_and_h.omega_result$ci.lower,

CI_Upper = mdhb.h_and_h.omega_result$ci.upper

)

# Combine results into a single table

mdhb.h_and_h.combined_table <- bind_rows(

data.frame(Measure = "Cronbach's Alpha", mdhb.h_and_h.alpha_table),

data.frame(Measure = "Omega", mdhb.h_and_h.omega_table)

)

# Create a kable table

mdhb.h_and_h.kable_table <- mdhb.h_and_h.combined_table %>%

kable("html", escape = FALSE, align = "c") %>%

kable_styling(full_width = FALSE)

mdhb.h_and_h.kable_table

| **Measure** | **Estimate** | **SE** | **CI_Lower** | **CI_Upper** |
| --- | --- | --- | --- | --- |
| Cronbach’s Alpha | 0.8990373 | 0.2236068 | 0.8435068 | 0.9348632 |
| Omega | 0.8998315 | 0.0242443 | 0.8523136 | 0.9473494 |

2.13.4. Material Deprivation and Hoarding Beliefs for the homelessness group

# Subset items for the homelessness group

mdhb.hm <- data3 %>%

filter(group == "HM") %>%

select(MDHB.1:MDHB.9)

## Cronbach's Alpha

mdhb.hm.alpha_result <- ci.reliability(data = mdhb.hm, type = "alpha", interval.type = "bonett")

mdhb.hm.alpha_table <- data.frame(

Estimate = mdhb.hm.alpha_result$est,

SE = mdhb.hm.alpha_result$se,

CI_Lower = mdhb.hm.alpha_result$ci.lower,

CI_Upper = mdhb.hm.alpha_result$ci.upper)

## Omega

mdhb.hm.omega_result <- ci.reliability(data = mdhb.hm, type = "omega", conf.level = 0.95)

mdhb.hm.omega_table <- data.frame(

Estimate = mdhb.hm.omega_result$est,

SE = mdhb.hm.omega_result$se,

CI_Lower = mdhb.hm.omega_result$ci.lower,

CI_Upper = mdhb.hm.omega_result$ci.upper)

# Combine results into a single table

mdhb.hm.combined_table <- bind_rows(

data.frame(Measure = "Cronbach's Alpha", mdhb.hm.alpha_table),

data.frame(Measure = "Omega", mdhb.hm.omega_table))

# Create a kable table

mdhb.hm.kable_table <- mdhb.hm.combined_table %>%

kable("html", escape = FALSE, align = "c") %>%

kable_styling(full_width = FALSE)

mdhb.hm.kable_table

| **Measure** | **Estimate** | **SE** | **CI_Lower** | **CI_Upper** |
| --- | --- | --- | --- | --- |
| Cronbach’s Alpha | 0.9126350 | 0.2465985 | 0.8583415 | 0.9461194 |
| Omega | 0.9154168 | 0.0185093 | 0.8791393 | 0.9516942 |

### R Script 3: Main statistical analysis script

**Contents**

1. Upload of prepared data file

2. Demographic information

3. Demographic profile of study groups

3.1. Group x sex assigned at birth

3.2. Group x sexual orientation

3.3. Group x ethnicity

3.4. Group x co-habitation

3.5. Group x education

3.6. Group x work status

3.7. Group x age

4. Descriptive of study variables for the whole sample

5. Depressive symptoms (PHQ) x Group

6. Anxiety symptoms (GAD) x Group

7. Work and Social Adjustment Scale x Group

8. Overall Hoarding (Savings Inventory-Revised) x Group

9. Levels of Clutter (Savings Inventory-Revised) x Group

10. Levels of Difficulty in Discarding Items (Savings Inventory-Revised) x Group

11. Levels of Excess of Acquisition (Savings Inventory-Revised) x Group

12. Exposure to Early Material Deprivation (Yes/No) x Group

13. Experiences of Early Material Deprivation (Continuous) x Group

14. Hypothesis 1

15. Hypothesis 2

**1. Upload of prepared data file**

# Dependencies

library(tidyverse)

library(knitr)

library(TMB)

library(readxl)

library(readr)

library(markdown)

library(kableExtra)

library(htmltools)

library(rmarkdown)

library(summarytools)

library(writexl)

library(scales)

library(gridExtra)

library(grid)

library(purrr)

library(magrittr)

library(jmv)

library(janitor)

library(effectsize)

library(stats)

library(car)

library(afex)

library(ggdist)

library(tidyquant)

# Jamovi R Package for statistical analysis:

#| https://jamovi.readthedocs.io/pt/latest/jmv/jmv_overview.html

#| https://www.jamovi.org/jmv/

# Parallel processing options are set appropriately for RStudio

# running on macOS with R version 4.0.0 or higher

**if** (Sys.getenv("RSTUDIO") == "1" && !nzchar(Sys.getenv("RSTUDIO_TERM")) &&

Sys.info()["sysname"] == "Darwin" && getRversion() >= "4.0.0") {

parallel:::setDefaultClusterOptions(setup_strategy = "sequential")}

# Setting workd directory

setwd("~/Library/CloudStorage/Dropbox/DClinPsy/TDRP/Data Analysis")

# Upload dataset

data3 <- read_excel("data3.xlsx") #

**2. Demographic information**

# Create white x other ethnicities variable

data3 <- data3 %>%

mutate(ethnicity.white = case_when(

grepl("White", ethnicity) ~ "White",

TRUE ~ "Other ethnicities"

)) %>%

mutate(ethnicity.white = as.factor(ethnicity.white))

# Create a birth sex data variable

data3 <- data3 %>%

mutate(birth.sex = case_when(

gender.same.birth == "Yes" ~ gender,

gender.same.birth == "No" & gender == "Female" ~ "Male",

gender.same.birth == "No" & gender == "Male" ~ "Female",

gender.same.birth == "Prefer not to say" ~ NA_character_,

TRUE ~ NA_character_ # Set any other value to missing (NA)

)) %>%

mutate(birth.sex = factor(birth.sex, levels = c("Male", "Female")))

# Create a binary education variable (uni x non-uni)

data3 <- data3 %>%

mutate(education.university = case_when(

education %in% c("Doctorate (e.g. PhD, EdD, DPhill)",

"Higher Degree (e.g. MA, MSc, PGCE)",

"Other postgraduate qualification",

"Undergraduate degree (e.g. BA, BSc)") ~ "Higher education",

education %in% c("Highers / A-levels / BTEC",

"National Vocational Qualification",

"SATs (Standardised Assessment Tests – Primary School)",

"Standard grades / GCSE / O-levels") ~ "Non-higher",

TRUE ~ NA_character_ # For "Prefer not to say" or "Unknown"

)) %>%

mutate(education.university = as.factor(education.university))

# Create a binary sexual orientation variable

data3 <- data3 %>%

mutate(sexual.orient.binary = case_when(

sexual.orient == "Heterosexual" ~ "Heterosexual",

TRUE ~ "Other"

)) %>%

mutate(sexual.orient.binary = factor(sexual.orient.binary, levels = c("Heterosexual", "Other")))

# Create a binary variable for co-habitation

data3 <- data3 %>%

mutate(live.with.binary = case_when(

live.with == "Live alone" ~ "Lives alone",

TRUE ~ "Shares residence"

)) %>%

mutate(live.with.binary = as.factor(live.with.binary))

# Create a binary variable for employment/work status

data3 <- data3 %>%

mutate(work.binary = case_when(

employment %in% c("Employed full-time", "Employed part-time", "Student") ~ "Working",

TRUE ~ "Not working"

)) %>%

mutate(work.binary = as.factor(work.binary))

# Select demographic variables for descriptions

demog <- data3 %>%

select(age, gender, gender.same.birth, birth.sex, sexual.orient, sexual.orient.binary,

ethnicity, ethnicity.white, live.with, live.with.binary, marital.status, education,

education.university, employment, work.binary, early.depriv.cat, homelessness, group) %>%

mutate_if(~ !is.numeric(.) || names(.) != "age", as.factor) # Convert all but 'age' to factors

# Create table of demographics

dfSummary(demog,

max.distinct.values = 30,

plain.ascii = FALSE,

graph.magnif = 0.75,

valid.col = FALSE,

style = "grid",

tmp.img.dir = "/tmp")

| **No** | **Variable** | **Stats / Values** | **Freqs (% of Valid)** | **Graph** | **Missing** |
| --- | --- | --- | --- | --- | --- |
| 1 | age [numeric] | Mean (sd) : 45.4 (13.5) min < med < max: 18 < 46 < 74 IQR (CV) : 21 (0.3) | 51 distinct values | 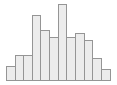 | 1 (0.8%) |
| 2 | gender [factor] | 1. Female 2. Male 3. Non-binary | 65 (50.4%) 60 (46.5%) 4 ( 3.1%) | 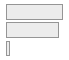 | 0 (0.0%) |
| 3 | gender.same.birth [factor] | 1. No 2. Prefer not to say 3. Yes | 4 ( 3.1%) 4 ( 3.1%) 121 (93.8%) | 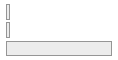 | 0 (0.0%) |
| 4 | birth.sex [factor] | 1. Male 2. Female | 60 (48.8%) 63 (51.2%) | 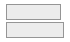 | 6 (4.7%) |
| 5 | sexual.orient [factor] | 1. Asexual 2. Bisexual 3. Heterosexual 4. Homosexual 5. Pansexual 6. Prefer not to say | 3 ( 2.4%) 10 ( 7.9%) 102 (80.3%) 7 ( 5.5%) 1 ( 0.8%) 4 ( 3.1%) | 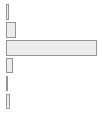 | 2 (1.6%) |
| 6 | sexual.orient.binary [factor] | 1. Heterosexual 2. Other | 102 (79.1%) 27 (20.9%) | 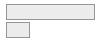 | 0 (0.0%) |
| 7 | ethnicity [factor] | 1. Arab 2. Bangladeshi or British Ba 3. Black (Black British / Bl 4. Mixed (White and Black Ca 5. Other 6. Other Asian background 7. Other Mixed / Multiple et 8. Pakistani or British Paki 9. Prefer not to say 10. Unsure 11. White (English / Welsh / 12. White Irish | 2 ( 1.6%) 1 ( 0.8%) 5 ( 3.9%) 8 ( 6.2%) 9 ( 7.0%) 1 ( 0.8%) 7 ( 5.4%) 3 ( 2.3%) 1 ( 0.8%) 1 ( 0.8%) 87 (67.4%) 4 ( 3.1%) | 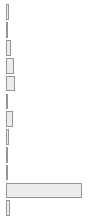 | 0 (0.0%) |
| 8 | ethnicity.white [factor] | 1. Other ethnicities 2. White | 30 (23.3%) 99 (76.7%) | 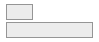 | 0 (0.0%) |
| 9 | live.with [factor] | 1. Halfway / group home 2. Homeless / shelter / couc 3. Live alone 4. Other 5. With flatmates 6. With own children 7. With parents 8. With partner 9. With siblings 10. With spouse / common law | 6 ( 4.7%) 39 (30.2%) 42 (32.6%) 8 ( 6.2%) 3 ( 2.3%) 9 ( 7.0%) 3 ( 2.3%) 5 ( 3.9%) 1 ( 0.8%) 13 (10.1%) | 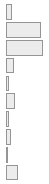 | 0 (0.0%) |
| 10 | live.with.binary [factor] | 1. Lives alone 2. Shares residence | 42 (32.6%) 87 (67.4%) | 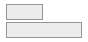 | 0 (0.0%) |
| 11 | marital.status [factor] | 1. Common-law marriage 2. Divorced 3. In a relationship (not ma 4. Married 5. Separated 6. Single (no partner) 7. Widowed | 3 ( 2.3%) 8 ( 6.2%) 17 (13.2%) 18 (14.0%) 7 ( 5.4%) 75 (58.1%) 1 ( 0.8%) | 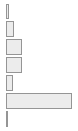 | 0 (0.0%) |
| 12 | education [factor] | 1. Doctorate (e.g. PhD, EdD, 2. Higher Degree (e.g. MA, M 3. Highers / A-levels / BTEC 4. National Vocational Quali 5. Other postgraduate qualif 6. Prefer not to say 7. SATs (Standardised Assess 8. Standard grades / GCSE / 9. Undergraduate degree (e.g 10. Unknown | 1 ( 0.8%) 20 (15.5%) 22 (17.1%) 11 ( 8.5%) 6 ( 4.7%) 3 ( 2.3%) 4 ( 3.1%) 31 (24.0%) 23 (17.8%) 8 ( 6.2%) | 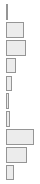 | 0 (0.0%) |
| 13 | education.university [factor] | 1. Higher education 2. Non-higher | 50 (42.4%) 68 (57.6%) | 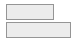 | 11 (8.5%) |
| 14 | employment [factor] | 1. Employed full-time 2. Employed part-time 3. Retired 4. Stay at home parent 5. Student 6. Unemployed and seeking wo 7. Unemployed due to disabil 8. Unknown | 25 (19.4%) 13 (10.1%) 15 (11.6%) 5 ( 3.9%) 2 ( 1.6%) 32 (24.8%) 35 (27.1%) 2 ( 1.6%) | 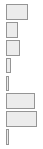 | 0 (0.0%) |
| 15 | work.binary [factor] | 1. Not working 2. Working | 89 (69.0%) 40 (31.0%) | 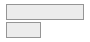 | 0 (0.0%) |
| 16 | early.depriv.cat [factor] | 1. No 2. Yes | 60 (46.9%) 68 (53.1%) | 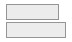 | 1 (0.8%) |
| 17 | homelessness [factor] | 1. No 2. Yes | 42 (32.8%) 86 (67.2%) | 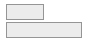 | 1 (0.8%) |
| 18 | group [factor] | 1. H&H 2. HD 3. HM | 47 (36.4%) 43 (33.3%) 39 (30.2%) | 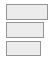 | 0 (0.0%) |

**3. Demographic profile of study groups**

*3.1. Group x sex assigned at birth*

# Chi-square Group x Sex

contTables(

formula = ~ group:birth.sex,

data = demog,

phiCra = TRUE,

pcRow = TRUE,

pcCol = TRUE,

barplot = TRUE,

yaxis = "ypc",

yaxisPc = "row_pc",

bartype = "stack")

##

## CONTINGENCY TABLES

##

## Contingency Tables

## ───────────────────────────────────────────────────────────────────

## group Male Female Total

## ───────────────────────────────────────────────────────────────────

## H&H Observed 26 18 44

## % within row 59.09091 40.90909 100.00000

## % within column 43.33333 28.57143 35.77236

##

## HD Observed 3 37 40

## % within row 7.50000 92.50000 100.00000

## % within column 5.00000 58.73016 32.52033

##

## HM Observed 31 8 39

## % within row 79.48718 20.51282 100.00000

## % within column 51.66667 12.69841 31.70732

##

## Total Observed 60 63 123

## % within row 48.78049 51.21951 100.00000

## % within column 100.00000 100.00000 100.00000

## ───────────────────────────────────────────────────────────────────

##

##

## χ² Tests

## ──────────────────────────────────────

## Value df p

## ──────────────────────────────────────

## χ² 43.87158 2 < .0000001

## N 123

## ──────────────────────────────────────

##

##

## Nominal

## ────────────────────────────────

## Value

## ────────────────────────────────

## Phi-coefficient NaN

## Cramer's V 0.5972265

## ────────────────────────────────


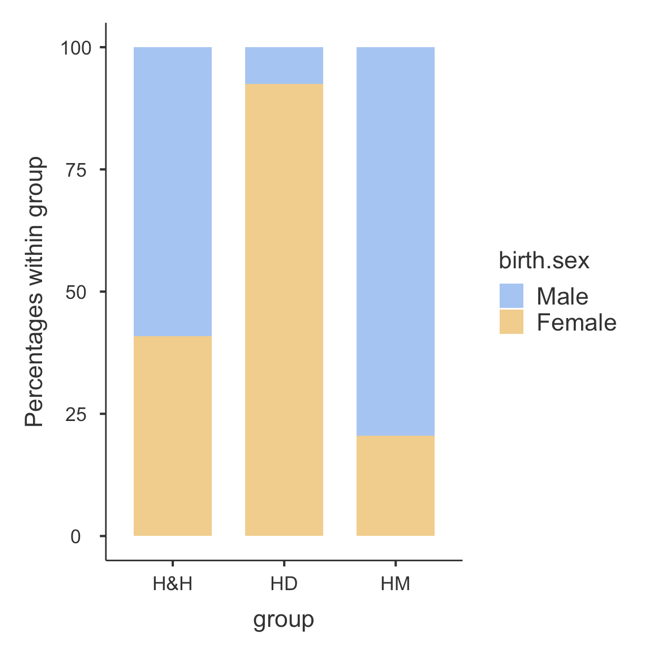


# Pairwise post-hoc analysis

# Create a list of group comparisons

comparisons <- list(

c("H&H", "HD"),

c("H&H", "HM"),

c("HD", "HM"))

# Function to perform chi-square test and format the results

pairwise_chisq <- **function**(data, group_var, sex_var, group1, group2) {

subset_data <- data %>% filter(!!sym(group_var) %in% c(group1, group2))

table_data <- table(subset_data[[group_var]], subset_data[[sex_var]])

test_result <- chisq.test(table_data)

data.frame(

Group_Comparison = paste(group1, "vs", group2),

Chi_Square = round(test_result$statistic, 3),

p_value = round(test_result$p.value, 3)

)

}

# Apply the function to all comparisons and compile results

results <- do.call(rbind, lapply(comparisons, **function**(comp) {

pairwise_chisq(data3, "group", "birth.sex", comp[1], comp[2])

}))

# Print the results using kableExtra

results %>%

kable("html", col.names = c("Group Comparison", "Chi-Square", "p-value")) %>%

kable_styling(full_width = F, position = "center")

|  | **Group Comparison** | **Chi-Square** | **p-value** |
| --- | --- | --- | --- |
| X-squared | H&H vs HD | 22.441 | 0.000 |
| X-squared1 | H&H vs HM | 3.106 | 0.078 |
| X-squared2 | HD vs HM | 38.857 | 0.000 |

*3.2. Group x sexual orientation*

# Chi-square Group x Sexual orientation

contTables(

formula = ~ group:sexual.orient.binary,

data = demog,

phiCra = TRUE,

pcRow = TRUE,

pcCol = TRUE,

barplot = TRUE,

yaxis = "ypc",

yaxisPc = "row_pc",

bartype = "stack")

## CONTINGENCY TABLES

##

## Contingency Tables

## ──────────────────────────────────────────────────────────────────────

## group Heterosexual Other Total

## ──────────────────────────────────────────────────────────────────────

## H&H Observed 34 13 47

## % within row 72.34043 27.65957 100.00000

## % within column 33.33333 48.14815 36.43411

##

## HD Observed 35 8 43

## % within row 81.39535 18.60465 100.00000

## % within column 34.31373 29.62963 33.33333

##

## HM Observed 33 6 39

## % within row 84.61538 15.38462 100.00000

## % within column 32.35294 22.22222 30.23256

##

## Total Observed 102 27 129

## % within row 79.06977 20.93023 100.00000

## % within column 100.00000 100.00000 100.00000

## ──────────────────────────────────────────────────────────────────────

##

## χ² Tests

## ─────────────────────────────────────

## Value df p

## ─────────────────────────────────────

## χ² 2.151311 2 0.3410741

## N 129

## ─────────────────────────────────────

##

## Nominal

## ────────────────────────────────

## Value

## ────────────────────────────────

## Phi-coefficient NaN

## Cramer's V 0.1291388

## ────────────────────────────────


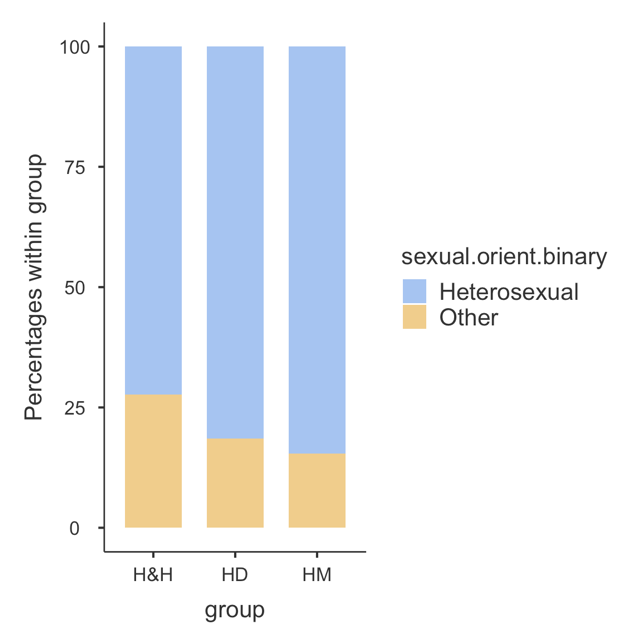


*3.3. Group x ethnicity*

# Chi-square Group x Ethnicity

contTables(

formula = ~ group:ethnicity.white,

data = demog,

phiCra = TRUE,

pcRow = TRUE,

pcCol = TRUE,

barplot = TRUE,

yaxis = "ypc",

yaxisPc = "row_pc",

bartype = "stack")

## CONTINGENCY TABLES

##

## Contingency Tables

## ───────────────────────────────────────────────────────────────────────────

## group Other ethnicities White Total

## ───────────────────────────────────────────────────────────────────────────

## H&H Observed 14 33 47

## % within row 29.78723 70.21277 100.00000

## % within column 46.66667 33.33333 36.43411

##

## HD Observed 9 34 43

## % within row 20.93023 79.06977 100.00000

## % within column 30.00000 34.34343 33.33333

##

## HM Observed 7 32 39

## % within row 17.94872 82.05128 100.00000

## % within column 23.33333 32.32323 30.23256

##

## Total Observed 30 99 129

## % within row 23.25581 76.74419 100.00000

## % within column 100.00000 100.00000 100.00000

## ───────────────────────────────────────────────────────────────────────────

##

## χ² Tests

## ─────────────────────────────────────

## Value df p

## ─────────────────────────────────────

## χ² 1.869170 2 0.3927489

## N 129

## ─────────────────────────────────────

##

## Nominal

## ────────────────────────────────

## Value

## ────────────────────────────────

## Phi-coefficient NaN

## Cramer's V 0.1203731

## ────────────────────────────────


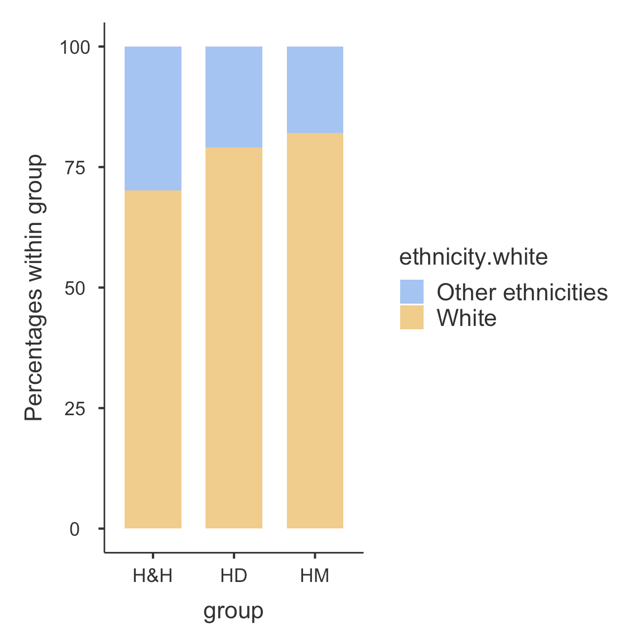


*3.4. Group x co-habitation*

# Chi-square Group x Co-habitation

contTables(

formula = ~ group:live.with.binary,

data = demog,

phiCra = TRUE,

pcRow = TRUE,

pcCol = TRUE,

barplot = TRUE,

yaxis = "ypc",

yaxisPc = "row_pc",

bartype = "stack")

## Contingency Tables

## ────────────────────────────────────────────────────────────────────────────

## group Lives alone Shares residence Total

## ────────────────────────────────────────────────────────────────────────────

## H&H Observed 15 32 47

## % within row 31.91489 68.08511 100.00000

## % within column 35.71429 36.78161 36.43411

##

## HD Observed 15 28 43

## % within row 34.88372 65.11628 100.00000

## % within column 35.71429 32.18391 33.33333

##

## HM Observed 12 27 39

## % within row 30.76923 69.23077 100.00000

## % within column 28.57143 31.03448 30.23256

##

## Total Observed 42 87 129

## % within row 32.55814 67.44186 100.00000

## % within column 100.00000 100.00000 100.00000

## ────────────────────────────────────────────────────────────────────────────

##

## χ² Tests

## ──────────────────────────────────────

## Value df p

## ──────────────────────────────────────

## χ² 0.1716076 2 0.9177743

## N 129

## ──────────────────────────────────────

##

## Nominal

## ─────────────────────────────────

## Value

## ─────────────────────────────────

## Phi-coefficient NaN

## Cramer's V 0.03647316

## ─────────────────────────────────


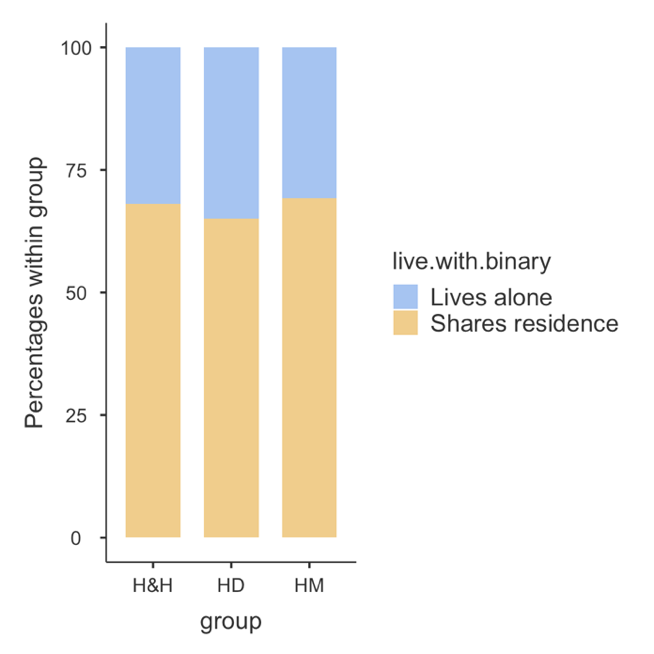


*3.5. Group x education*

# Chi-square Group x Education

contTables(

formula = ~ group:education.university,

data = demog,

phiCra = TRUE,

pcRow = TRUE,

pcCol = TRUE,

barplot = TRUE,

yaxis = "ypc",

yaxisPc = "row_pc",

bartype = "stack")

## CONTINGENCY TABLES

##

## Contingency Tables

## ───────────────────────────────────────────────────────────────────────────

## group Higher education Non-higher Total

## ───────────────────────────────────────────────────────────────────────────

## H&H Observed 13 29 42

## % within row 30.95238 69.04762 100.00000

## % within column 26.00000 42.64706 35.59322

##

## HD Observed 26 14 40

## % within row 65.00000 35.00000 100.00000

## % within column 52.00000 20.58824 33.89831

##

## HM Observed 11 25 36

## % within row 30.55556 69.44444 100.00000

## % within column 22.00000 36.76471 30.50847

##

## Total Observed 50 68 118

## % within row 42.37288 57.62712 100.00000

## % within column 100.00000 100.00000 100.00000

## ───────────────────────────────────────────────────────────────────────────

##

## χ² Tests

## ─────────────────────────────────────

## Value df p

## ─────────────────────────────────────

## χ² 12.68919 2 0.0017562

## N 118

## ─────────────────────────────────────

##

## Nominal

## ────────────────────────────────

## Value

## ────────────────────────────────

## Phi-coefficient NaN

## Cramer's V 0.3279260

## ────────────────────────────────


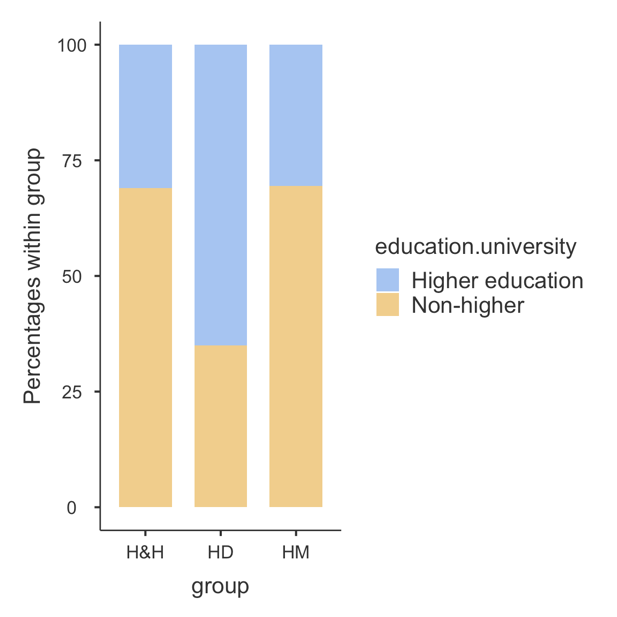


# Pairwise post-hoc analysis

# Create a list of group comparisons

comparisons <- list(

c("H&H", "HD"),

c("H&H", "HM"),

c("HD", "HM"))

# Function to perform chi-square test and format the results

pairwise_edu_chisq <- **function**(data, group_var, edu_var, group1, group2) {

subset_data <- data %>% filter(!!sym(group_var) %in% c(group1, group2))

table_data <- table(subset_data[[group_var]], subset_data[[edu_var]])

test_result <- chisq.test(table_data)

data.frame(

Group_Comparison = paste(group1, "vs", group2),

Chi_Square = round(test_result$statistic, 3),

p_value = round(test_result$p.value, 3)

)

}

# Apply the function to all comparisons and compile results

results <- do.call(rbind, lapply(comparisons, **function**(comp) {

pairwise_edu_chisq(data3, "group", "education.university", comp[1], comp[2])

}))

# Print the results using kableExtra

results %>%

kable("html", col.names = c("Group Comparison", "Chi-Square", "p-value")) %>%

kable_styling(full_width = F, position = "center")

|  | **Group Comparison** | **Chi-Square** | **p-value** |
| --- | --- | --- | --- |
| X-squared | H&H vs HD | 8.207 | 0.004 |
| X-squared1 | H&H vs HM | 0.000 | 1.000 |
| X-squared2 | HD vs HM | 7.672 | 0.006 |

*3.6. Group x work status*

# Chi-square Group x Work status

contTables(

formula = ~ group:work.binary,

data = demog,

phiCra = TRUE,

pcRow = TRUE,

pcCol = TRUE,

barplot = TRUE,

yaxis = "ypc",

yaxisPc = "row_pc",

bartype = "stack")

## CONTINGENCY TABLES

##

## Contingency Tables

## ─────────────────────────────────────────────────────────────────────

## group Not working Working Total

## ─────────────────────────────────────────────────────────────────────

## H&H Observed 36 11 47

## % within row 76.59574 23.40426 100.00000

## % within column 40.44944 27.50000 36.43411

##

## HD Observed 23 20 43

## % within row 53.48837 46.51163 100.00000

## % within column 25.84270 50.00000 33.33333

##

## HM Observed 30 9 39

## % within row 76.92308 23.07692 100.00000

## % within column 33.70787 22.50000 30.23256

##

## Total Observed 89 40 129

## % within row 68.99225 31.00775 100.00000

## % within column 100.00000 100.00000 100.00000

## ─────────────────────────────────────────────────────────────────────

##

## χ² Tests

## ─────────────────────────────────────

## Value df p

## ─────────────────────────────────────

## χ² 7.248259 2 0.0266723

## N 129

## ─────────────────────────────────────

##

## Nominal

## ────────────────────────────────

## Value

## ────────────────────────────────

## Phi-coefficient NaN

## Cramer's V 0.2370402

## ────────────────────────────────


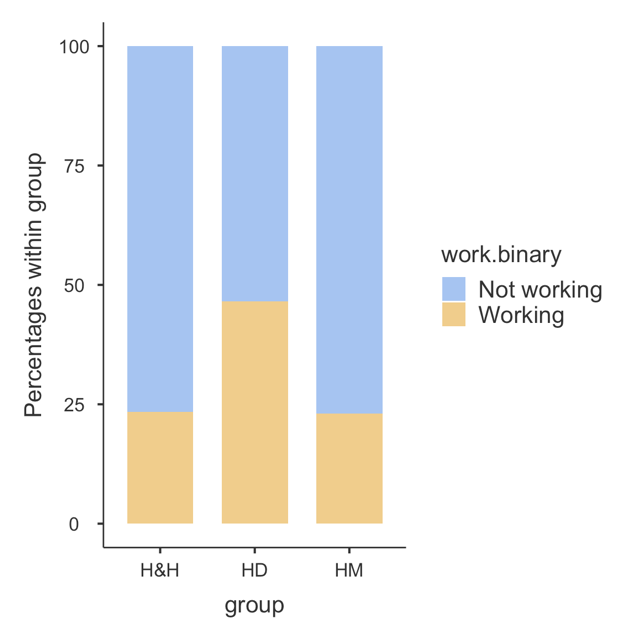


# Pairwise post-hoc analysis

# Create a list of group comparisons

comparisons <- list(

c("H&H", "HD"),

c("H&H", "HM"),

c("HD", "HM"))

# Function to perform chi-square test and format the results

pairwise_work_chisq <- **function**(data, group_var, work_var, group1, group2) {

subset_data <- data %>% filter(!!sym(group_var) %in% c(group1, group2))

table_data <- table(subset_data[[group_var]], subset_data[[work_var]])

test_result <- chisq.test(table_data)

data.frame(

Group_Comparison = paste(group1, "vs", group2),

Chi_Square = round(test_result$statistic, 3),

p_value = round(test_result$p.value, 3)

)

}

# Apply the function to all comparisons and compile results

results <- do.call(rbind, lapply(comparisons, **function**(comp) {

pairwise_work_chisq(data3, "group", "work.binary", comp[1], comp[2])

}))

# Print the results using kableExtra

results %>%

kable("html", col.names = c("Group Comparison", "Chi-Square", "p-value")) %>%

kable_styling(full_width = F, position = "center")

|  | **Group Comparison** | **Chi-Square** | **p-value** |
| --- | --- | --- | --- |
| X-squared | H&H vs HD | 4.336 | 0.037 |
| X-squared1 | H&H vs HM | 0.000 | 1.000 |
| X-squared2 | HD vs HM | 3.942 | 0.047 |

*3.7. Group x age*

# One-Way ANOVA Group x Age

anovaOneW(

formula = age ~ group,

data = demog,

desc = TRUE,

descPlot = TRUE,

norm = TRUE,

qq = FALSE,

eqv = TRUE,

phMethod = "tukey",

phTest = TRUE)

## ONE-WAY ANOVA

##

## One-Way ANOVA (Welch's)

## ───────────────────────────────────────────────────

## F df1 df2 p

## ───────────────────────────────────────────────────

## age 13.14986 2 82.93099 0.0000110

## ───────────────────────────────────────────────────

##

## Group Descriptives

## ──────────────────────────────────────────────────────────

## group N Mean SD SE

## ──────────────────────────────────────────────────────────

## age H&H 46 42.56522 12.63795 1.863365

## HD 43 53.18605 12.76949 1.947329

## HM 39 40.02564 11.63137 1.862510

## ──────────────────────────────────────────────────────────

##

## ASSUMPTION CHECKS

##

## Normality Test (Shapiro-Wilk)

## ─────────────────────────────────

## W p

## ─────────────────────────────────

## age 0.9890334 0.4036654

## ─────────────────────────────────

## Note. A low p-value

## suggests a violation of the

## assumption of normality

##

## Homogeneity of Variances Test (Levene's)

## ───────────────────────────────────────────────

## F df1 df2 p

## ───────────────────────────────────────────────

## age 0.1999248 2 125 0.8190536

## ───────────────────────────────────────────────

##

## POST HOC TESTS

##

## Tukey Post-Hoc Test – age

## ─────────────────────────────────────────────────────────────────

## H&H HD HM

## ─────────────────────────────────────────────────────────────────

## H&H Mean difference — -10.62083 2.539576

## t-value — -4.042440 0.9419548

## df — 125.0000 125.0000

## p-value — 0.0002694 0.6148537

##

## HD Mean difference — 13.160405

## t-value — 4.8050280

## df — 125.0000

## p-value — 0.0000129

##

## HM Mean difference —

## t-value —

## df —

## p-value —

## ─────────────────────────────────────────────────────────────────


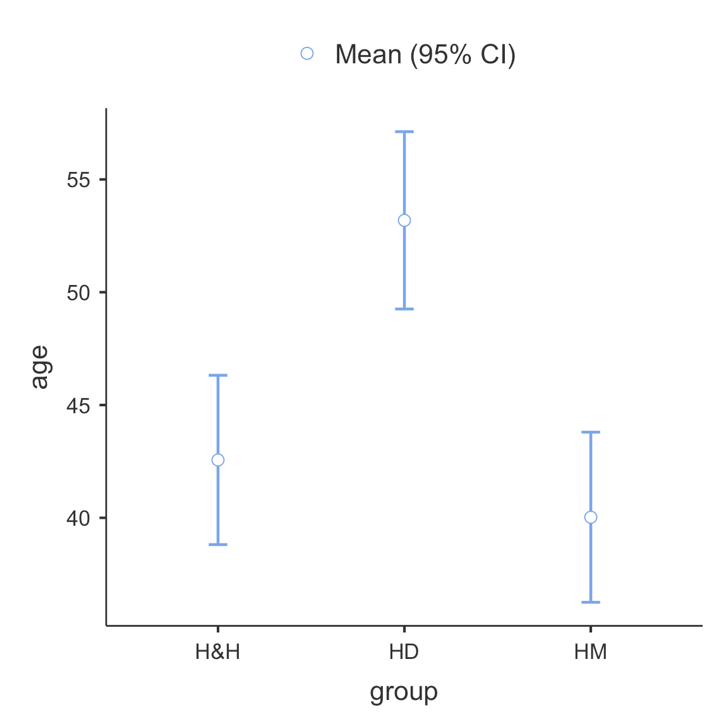


**4. Descriptive statistics of study variables for the whole sample**

# Selecting variables for descriptive statistics

descript.data3 <- data3 %>%

select(phq.total, gad.total, wsas.total, bah.harm.avoi, bah.fear.mat.depr, bah.att.dist, sir.total, sir.clutter, sir.diffic.discard, sir.exces.acquis, eemdq.total, housing.total)

# Descriptive statistics

descr(descript.data3,

stats = c("mean", "sd", "Med", "min", "max"),

transpose = TRUE)

N: 129

|  | **Mean** | **Std.Dev** | **Median** | **Min** | **Max** |
| --- | --- | --- | --- | --- | --- |
| bah.att.dist | 46.77 | 24.93 | 46.70 | 0.00 | 95.80 |
| bah.fear.mat.depr | 47.93 | 23.45 | 46.70 | 0.00 | 94.40 |
| bah.harm.avoi | 32.88 | 22.85 | 28.30 | 0.00 | 88.30 |
| eemdq.total | 13.23 | 11.49 | 12.00 | 0.00 | 42.00 |
| gad.total | 9.54 | 5.33 | 9.00 | 0.00 | 21.00 |
| housing.total | 2.20 | 2.10 | 2.00 | 0.00 | 9.00 |
| phq.total | 11.19 | 5.83 | 11.00 | 0.00 | 24.00 |
| sir.clutter | 16.91 | 9.94 | 18.00 | 0.00 | 35.00 |
| sir.diffic.discard | 14.64 | 7.33 | 16.00 | 0.00 | 28.00 |
| sir.exces.acquis | 13.09 | 6.06 | 14.00 | 1.00 | 25.00 |
| sir.total | 44.65 | 21.05 | 47.00 | 1.00 | 85.00 |
| wsas.total | 17.95 | 9.17 | 18.00 | 0.00 | 36.00 |

5. Depressive symptoms (PHQ) x Group

# One-Way ANOVA PHQ x Group

anovaOneW(

formula = phq.total ~ group,

data = data3,

desc = TRUE,

descPlot = TRUE,

norm = TRUE,

qq = FALSE,

eqv = TRUE,

phMethod = "tukey",

phTest = TRUE)

## ONE-WAY ANOVA

##

## One-Way ANOVA (Welch's)

## ─────────────────────────────────────────────────────────

## F df1 df2 p

## ─────────────────────────────────────────────────────────

## phq.total 8.276072 2 82.40591 0.0005305

## ─────────────────────────────────────────────────────────

##

## Group Descriptives

## ──────────────────────────────────────────────────────────────────

## group N Mean SD SE

## ──────────────────────────────────────────────────────────────────

## phq.total H&H 47 13.659574 5.470381 0.7979371

## HD 43 10.674419 5.326130 0.8122272

## HM 39 8.794872 5.768241 0.9236578

## ──────────────────────────────────────────────────────────────────

##

## ASSUMPTION CHECKS

##

## Normality Test (Shapiro-Wilk)

## ───────────────────────────────────────

## W p

## ───────────────────────────────────────

## phq.total 0.9907393 0.5483236

## ───────────────────────────────────────

## Note. A low p-value suggests a

## violation of the assumption of

## normality

##

## Homogeneity of Variances Test (Levene's)

## ─────────────────────────────────────────────────────

## F df1 df2 p

## ─────────────────────────────────────────────────────

## phq.total 0.2219042 2 126 0.8013045

## ─────────────────────────────────────────────────────

##

## POST HOC TESTS

##

## Tukey Post-Hoc Test – phq.total

## ─────────────────────────────────────────────────────────────────

## H&H HD HM

## ─────────────────────────────────────────────────────────────────

## H&H Mean difference — 2.985156 4.864703

## t-value — 2.564965 4.072309

## df — 126.0000 126.0000

## p-value — 0.0306672 0.0002398

##

## HD Mean difference — 1.879547

## t-value — 1.541222

## df — 126.0000

## p-value — 0.2752803

## ─────────────────────────────────────────────────────────────────


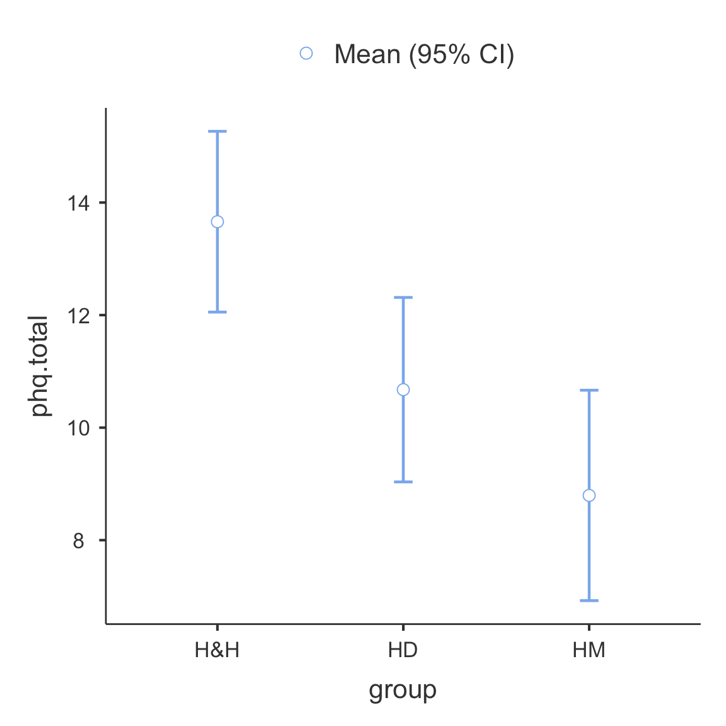


**6. Anxiety symptoms (GAD) x Group**

# One-Way ANOVA GAD x Group

anovaOneW(

formula = gad.total ~ group,

data = data3,

desc = TRUE,

descPlot = TRUE,

norm = TRUE,

qq = FALSE,

eqv = TRUE,

phMethod = "tukey",

phTest = TRUE)

## ONE-WAY ANOVA

##

## One-Way ANOVA (Welch's)

## ─────────────────────────────────────────────────────────

## F df1 df2 p

## ─────────────────────────────────────────────────────────

## gad.total 13.37022 2 82.06219 0.0000094

## ─────────────────────────────────────────────────────────

##

##

## Group Descriptives

## ──────────────────────────────────────────────────────────────────

## group N Mean SD SE

## ──────────────────────────────────────────────────────────────────

## gad.total H&H 47 12.446809 4.866963 0.7099195

## HD 43 7.674419 4.534108 0.6914451

## HM 39 8.102564 5.255548 0.8415612

## ──────────────────────────────────────────────────────────────────

##

##

## ASSUMPTION CHECKS

##

## Normality Test (Shapiro-Wilk)

## ───────────────────────────────────────

## W p

## ───────────────────────────────────────

## gad.total 0.9828021 0.1019058

## ───────────────────────────────────────

## Note. A low p-value suggests a

## violation of the assumption of

## normality

##

##

## Homogeneity of Variances Test (Levene's)

## ─────────────────────────────────────────────────────

## F df1 df2 p

## ─────────────────────────────────────────────────────

## gad.total 0.9750314 2 126 0.3800079

## ─────────────────────────────────────────────────────

##

##

## POST HOC TESTS

##

## Tukey Post-Hoc Test – gad.total

## ──────────────────────────────────────────────────────────────────

## H&H HD HM

## ──────────────────────────────────────────────────────────────────

## H&H Mean difference — 4.772390 4.3442444

## t-value — 4.632664 4.1084620

## df — 126.0000 126.0000

## p-value — 0.0000263 0.0002092

##

## HD Mean difference — -0.4281455

## t-value — -0.3966286

## df — 126.0000

## p-value — 0.9170043

##

## HM Mean difference —

## t-value —

## df —

## p-value —

## ──────────────────────────────────────────────────────────────────


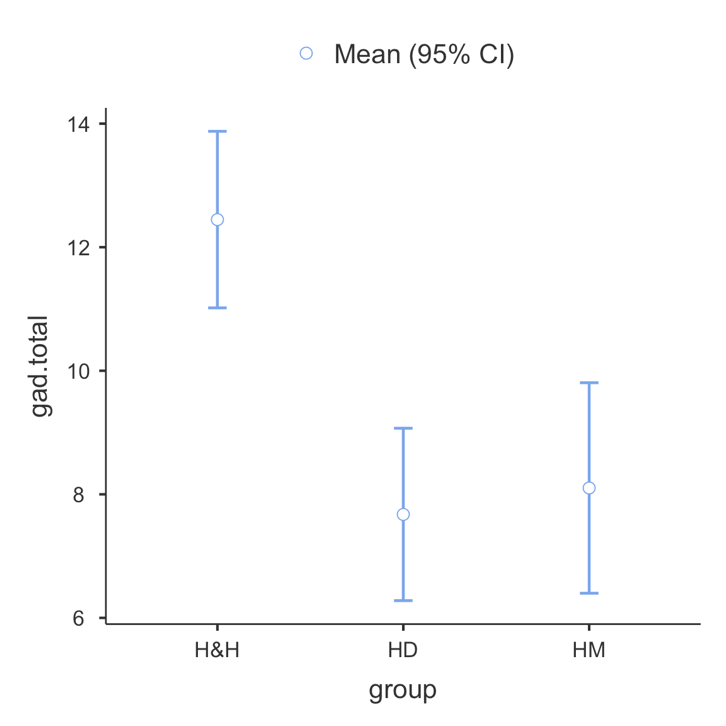


**7. Work and Social Adjustment Scale x Group**

# # One-Way ANOVA

anovaOneW(

formula = wsas.total ~ group,

data = data3,

desc = TRUE,

descPlot = TRUE,

norm = TRUE,

qq = FALSE,

eqv = TRUE,

phMethod = "tukey",

phTest = TRUE)

## One-Way ANOVA (Welch's)

## ──────────────────────────────────────────────────────────

## F df1 df2 p

## ──────────────────────────────────────────────────────────

## wsas.total 6.097986 2 82.56369 0.0033879

## ──────────────────────────────────────────────────────────

##

## Group Descriptives

## ─────────────────────────────────────────────────────────────────

## group N Mean SD SE

## ─────────────────────────────────────────────────────────────────

## wsas.total H&H 47 20.93617 8.911166 1.299827

## HD 43 18.18605 8.341679 1.272094

## HM 39 14.07692 9.149014 1.465015

## ─────────────────────────────────────────────────────────────────

##

## ASSUMPTION CHECKS

##

## Normality Test (Shapiro-Wilk)

## ────────────────────────────────────────

## W p

## ────────────────────────────────────────

## wsas.total 0.9913975 0.6130393

## ────────────────────────────────────────

## Note. A low p-value suggests a

## violation of the assumption of

## normality

##

## Homogeneity of Variances Test (Levene's)

## ──────────────────────────────────────────────────────

## F df1 df2 p

## ──────────────────────────────────────────────────────

## wsas.total 0.5911090 2 126 0.5552409

## ──────────────────────────────────────────────────────

##

## POST HOC TESTS

##

## Tukey Post-Hoc Test – wsas.total

## ─────────────────────────────────────────────────────────────────

## H&H HD HM

## ─────────────────────────────────────────────────────────────────

## H&H Mean difference — 2.750124 6.859247

## t-value — 1.481022 3.598785

## df — 126.0000 126.0000

## p-value — 0.3034587 0.0013228

##

## HD Mean difference — 4.109123

## t-value — 2.111817

## df — 126.0000

## p-value — 0.0914975

##

## HM Mean difference —

## t-value —

## df —

## p-value —

## ─────────────────────────────────────────────────────────────────


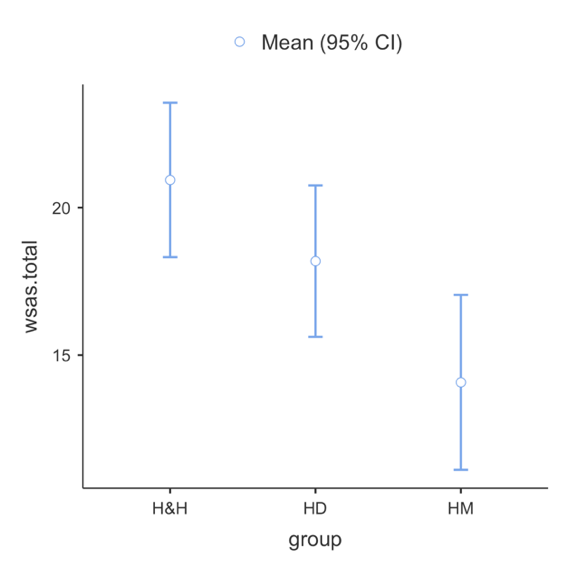


**8. Overall Hoarding (Savings Inventory-Revised) x Group**

# Non-Parametric ANOVA

anovaNP(

formula = sir.total ~ group,

data = data3,

es = TRUE,

pairs = TRUE)

## ONE-WAY ANOVA (NON-PARAMETRIC)

##

## Kruskal-Wallis

## ──────────────────────────────────────────────────────────

## χ² df p ε²

## ──────────────────────────────────────────────────────────

## sir.total 63.29984 2 < .0000001 0.4945300

## ──────────────────────────────────────────────────────────

##

## DWASS-STEEL-CRITCHLOW-FLIGNER PAIRWISE COMPARISONS

##

## Pairwise comparisons - sir.total

## ─────────────────────────────────────────

## W p

## ─────────────────────────────────────────

## H&H HD 4.229123 0.0078553

## H&H HM -8.289287 < .0000001

## HD HM -10.382822 < .0000001

## ─────────────────────────────────────────

# One-Way ANOVA

anovaOneW(

formula = sir.total ~ group,

data = data3,

desc = TRUE,

descPlot = TRUE,

norm = TRUE,

qq = FALSE,

eqv = TRUE,

phTest = FALSE)

## ONE-WAY ANOVA

##

## One-Way ANOVA (Welch's)

## ──────────────────────────────────────────────────────────

## F df1 df2 p

## ──────────────────────────────────────────────────────────

## sir.total 78.73511 2 81.74793 < .0000001

## ──────────────────────────────────────────────────────────

##

## Group Descriptives

## ────────────────────────────────────────────────────────────────

## group N Mean SD SE

## ────────────────────────────────────────────────────────────────

## sir.total H&H 47 49.19149 17.05699 2.488017

## HD 43 59.62791 11.71695 1.786818

## HM 39 22.66667 14.61854 2.340840

## ────────────────────────────────────────────────────────────────

##

## ASSUMPTION CHECKS

##

## Normality Test (Shapiro-Wilk)

## ───────────────────────────────────────

## W p

## ───────────────────────────────────────

## sir.total 0.9822338 0.0894929

## ───────────────────────────────────────

## Note. A low p-value suggests a

## violation of the assumption of normality.

##

## Homogeneity of Variances Test (Levene's)

## ────────────────────────────────────────────────────

## F df1 df2 p

## ────────────────────────────────────────────────────

## sir.total 4.285460 2 126 0.0158277

## ────────────────────────────────────────────────────


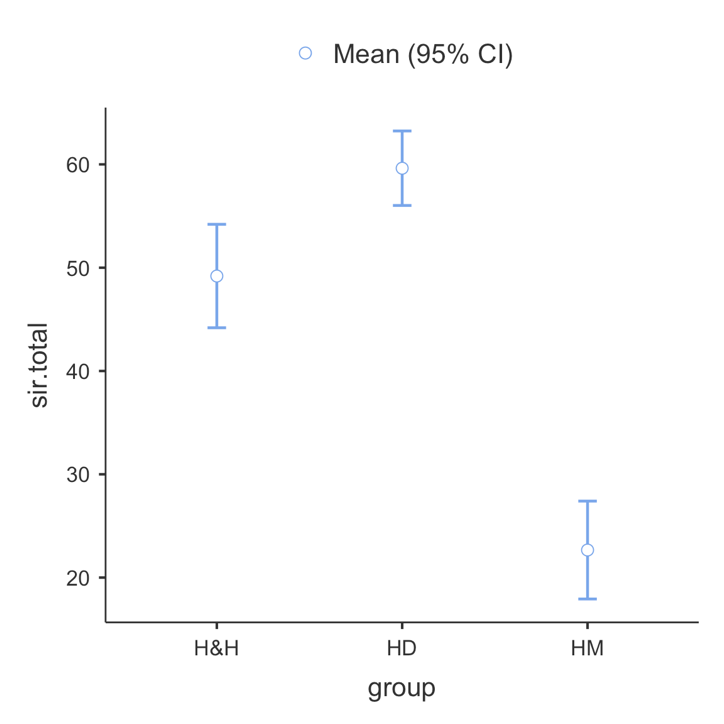


**9. Levels of Clutter (Savings Inventory-Revised) x Group**

# Non-Parametric ANOVA

anovaNP(

formula = sir.clutter ~ group,

data = data3,

es = TRUE,

pairs = TRUE)

## ONE-WAY ANOVA (NON-PARAMETRIC)

##

## Kruskal-Wallis

## ────────────────────────────────────────────────────────────

## χ² df p ε²

## ────────────────────────────────────────────────────────────

## sir.clutter 63.95600 2 < .0000001 0.4996563

## ────────────────────────────────────────────────────────────

##

##

## DWASS-STEEL-CRITCHLOW-FLIGNER PAIRWISE COMPARISONS

##

## Pairwise comparisons - sir.clutter

## ─────────────────────────────────────────

## W p

## ─────────────────────────────────────────

## H&H HD 5.402909 0.0003921

## H&H HM -7.993999 < .0000001

## HD HM -10.211957 < .0000001

## ─────────────────────────────────────────

# One-Way ANOVA

anovaOneW(

formula = sir.clutter ~ group,

data = data3,

desc = TRUE,

descPlot = TRUE,

norm = TRUE,

qq = FALSE,

eqv = TRUE,

phTest = FALSE)

## ONE-WAY ANOVA

##

## One-Way ANOVA (Welch's)

## ────────────────────────────────────────────────────────────

## F df1 df2 p

## ────────────────────────────────────────────────────────────

## sir.clutter 81.35951 2 83.87788 < .0000001

## ────────────────────────────────────────────────────────────

##

##

## Group Descriptives

## ────────────────────────────────────────────────────────────────────

## group N Mean SD SE

## ────────────────────────────────────────────────────────────────────

## sir.clutter H&H 47 17.978723 8.409336 1.2266277

## HD 43 24.581395 6.492606 0.9901131

## HM 39 7.179487 5.928840 0.9493742

## ────────────────────────────────────────────────────────────────────

##

##

## ASSUMPTION CHECKS

##

## Normality Test (Shapiro-Wilk)

## ─────────────────────────────────────────

## W p

## ─────────────────────────────────────────

## sir.clutter 0.9881259 0.3308270

## ─────────────────────────────────────────

## Note. A low p-value suggests a

## violation of the assumption of

## normality

##

##

## Homogeneity of Variances Test (Levene's)

## ──────────────────────────────────────────────────────

## F df1 df2 p

## ──────────────────────────────────────────────────────

## sir.clutter 4.209493 2 126 0.0169952

## ──────────────────────────────────────────────────────


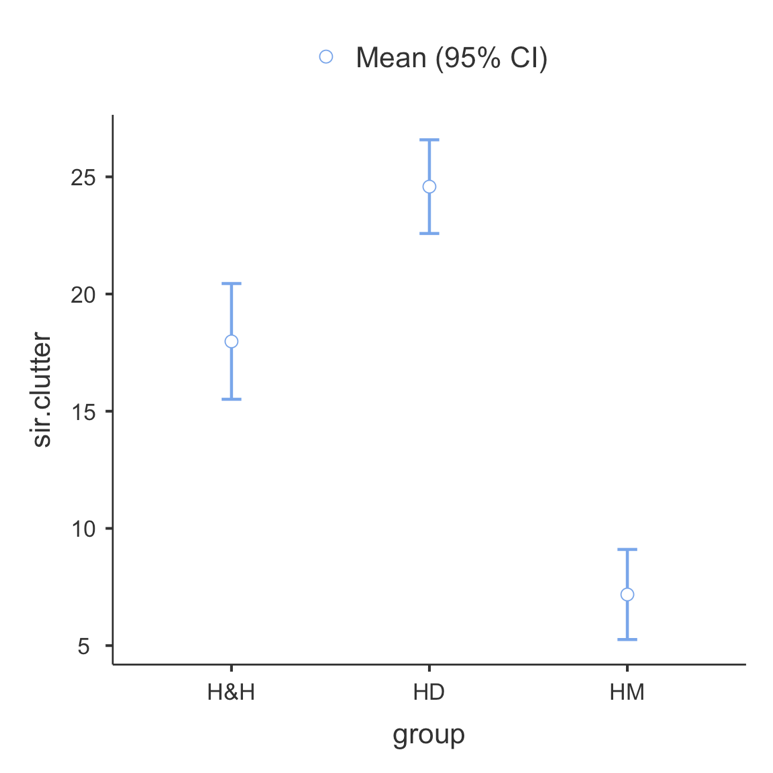


10. Levels of Difficulty in Discarding Items (Savings Inventory-Revised) x Group

# Non-Parametric ANOVA

anovaNP(

formula = sir.diffic.discard ~ group,

data = data3,

es = TRUE,

pairs = TRUE)

## ONE-WAY ANOVA (NON-PARAMETRIC)

##

## Kruskal-Wallis

## ───────────────────────────────────────────────────────────────────

## χ² df p ε²

## ───────────────────────────────────────────────────────────────────

## sir.diffic.discard 63.62365 2 < .0000001 0.4970598

## ───────────────────────────────────────────────────────────────────

##

##

## DWASS-STEEL-CRITCHLOW-FLIGNER PAIRWISE COMPARISONS

##

## Pairwise comparisons - sir.diffic.discard

## ─────────────────────────────────────────

## W p

## ─────────────────────────────────────────

## H&H HD 5.034831 0.0010804

## H&H HM -8.193648 < .0000001

## HD HM -10.189326 < .0000001

## ─────────────────────────────────────────

# One-Way ANOVA

anovaOneW(

formula = sir.diffic.discard ~ group,

data = data3,

desc = TRUE,

descPlot = TRUE,

norm = TRUE,

qq = FALSE,

eqv = TRUE,

phTest = FALSE)

## ONE-WAY ANOVA

##

## One-Way ANOVA (Welch's)

## ───────────────────────────────────────────────────────────────────

## F df1 df2 p

## ───────────────────────────────────────────────────────────────────

## sir.diffic.discard 64.85412 2 80.73630 < .0000001

## ───────────────────────────────────────────────────────────────────

##

##

## Group Descriptives

## ───────────────────────────────────────────────────────────────────────────

## group N Mean SD SE

## ───────────────────────────────────────────────────────────────────────────

## sir.diffic.discard H&H 47 16.063830 5.439007 0.7933607

## HD 43 19.930233 4.165592 0.6352469

## HM 39 7.102564 5.775606 0.9248371

## ───────────────────────────────────────────────────────────────────────────

##

##

## ASSUMPTION CHECKS

##

## Normality Test (Shapiro-Wilk)

## ────────────────────────────────────────────────

## W p

## ────────────────────────────────────────────────

## sir.diffic.discard 0.9849489 0.1658035

## ────────────────────────────────────────────────

## Note. A low p-value suggests a violation

## of the assumption of normality

##

##

## Homogeneity of Variances Test (Levene's)

## ─────────────────────────────────────────────────────────────

## F df1 df2 p

## ─────────────────────────────────────────────────────────────

## sir.diffic.discard 4.072274 2 126 0.0193305

## ─────────────────────────────────────────────────────────────


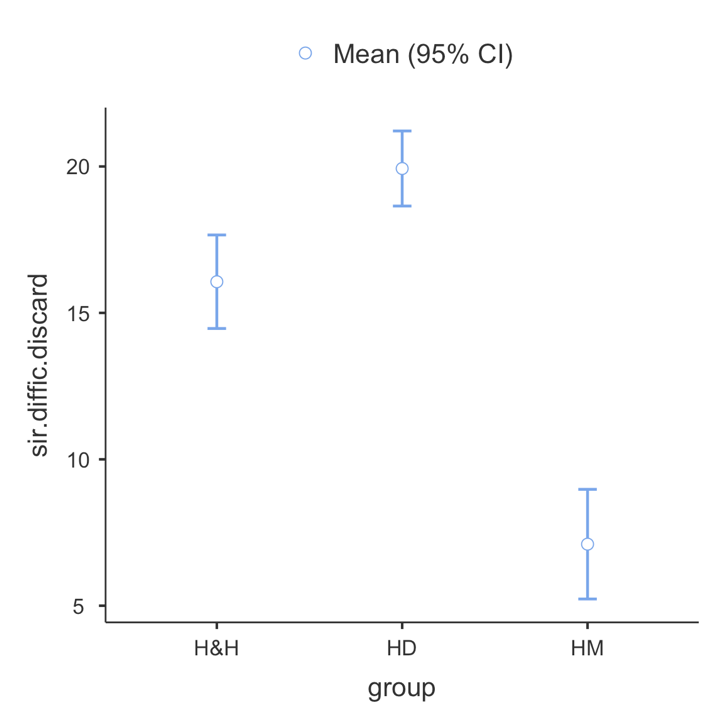


**11. Levels of Excess of Acquisition (Savings Inventory-Revised) x Group**

# Non-Parametric ANOVA

anovaNP(

formula = sir.exces.acquis ~ group,

data = data3,

es = TRUE,

pairs = TRUE)

##

## ONE-WAY ANOVA (NON-PARAMETRIC)

##

## Kruskal-Wallis

## ─────────────────────────────────────────────────────────────────

## χ² df p ε²

## ─────────────────────────────────────────────────────────────────

## sir.exces.acquis 32.87517 2 < .0000001 0.2568372

## ─────────────────────────────────────────────────────────────────

##

##

## DWASS-STEEL-CRITCHLOW-FLIGNER PAIRWISE COMPARISONS

##

## Pairwise comparisons - sir.exces.acquis

## ────────────────────────────────────────

## W p

## ────────────────────────────────────────

## H&H HD -0.1602369 0.9930118

## H&H HM -7.0395204 0.0000019

## HD HM -7.1501719 0.0000013

## ────────────────────────────────────────

# One-Way ANOVA

anovaOneW(

formula = sir.exces.acquis ~ group,

data = data3,

desc = TRUE,

descPlot = TRUE,

norm = TRUE,

qq = FALSE,

eqv = TRUE,

phTest = FALSE)

## ONE-WAY ANOVA

##

## One-Way ANOVA (Welch's)

## ─────────────────────────────────────────────────────────────────

## F df1 df2 p

## ─────────────────────────────────────────────────────────────────

## sir.exces.acquis 23.50959 2 83.41970 < .0000001

## ─────────────────────────────────────────────────────────────────

##

##

## Group Descriptives

## ─────────────────────────────────────────────────────────────────────────

## group N Mean SD SE

## ─────────────────────────────────────────────────────────────────────────

## sir.exces.acquis H&H 47 15.148936 5.433306 0.7925291

## HD 43 15.116279 5.233633 0.7981216

## HM 39 8.384615 5.013746 0.8028419

## ─────────────────────────────────────────────────────────────────────────

##

##

## ASSUMPTION CHECKS

##

## Normality Test (Shapiro-Wilk)

## ──────────────────────────────────────────────

## W p

## ──────────────────────────────────────────────

## sir.exces.acquis 0.9783830 0.0371215

## ──────────────────────────────────────────────

## Note. A low p-value suggests a violation

## of the assumption of normality

##

##

## Homogeneity of Variances Test (Levene's)

## ────────────────────────────────────────────────────────────

## F df1 df2 p

## ────────────────────────────────────────────────────────────

## sir.exces.acquis 0.2646050 2 126 0.7679345

## ────────────────────────────────────────────────────────────


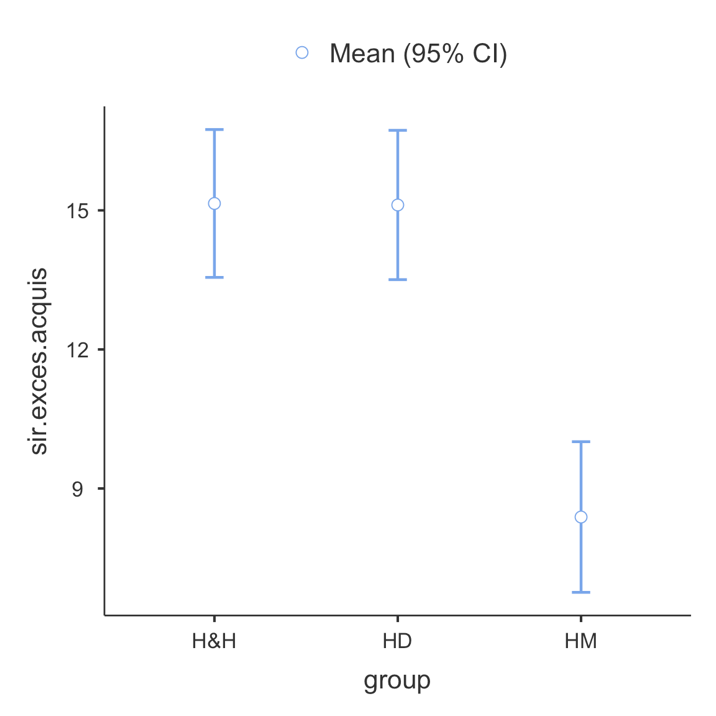


**12. Exposure to Early Material Deprivation (Yes/No) x Group**

# Chi-square Group x Work status

contTables(

formula = ~ group:early.depriv.cat,

data = demog,

phiCra = TRUE,

pcRow = TRUE,

pcCol = TRUE,

barplot = TRUE,

yaxis = "ypc",

yaxisPc = "row_pc",

bartype = "stack")

## CONTINGENCY TABLES

##

## Contingency Tables

## ───────────────────────────────────────────────────────────────────

## group No Yes Total

## ───────────────────────────────────────────────────────────────────

## H&H Observed 15 32 47

## % within row 31.91489 68.08511 100.00000

## % within column 25.00000 47.05882 36.71875

##

## HD Observed 24 18 42

## % within row 57.14286 42.85714 100.00000

## % within column 40.00000 26.47059 32.81250

##

## HM Observed 21 18 39

## % within row 53.84615 46.15385 100.00000

## % within column 35.00000 26.47059 30.46875

##

## Total Observed 60 68 128

## % within row 46.87500 53.12500 100.00000

## % within column 100.00000 100.00000 100.00000

## ───────────────────────────────────────────────────────────────────

##

##

## χ² Tests

## ─────────────────────────────────────

## Value df p

## ─────────────────────────────────────

## χ² 6.763267 2 0.0339919

## N 128

## ─────────────────────────────────────

##

##

## Nominal

## ────────────────────────────────

## Value

## ────────────────────────────────

## Phi-coefficient NaN

## Cramer's V 0.2298652

## ────────────────────────────────


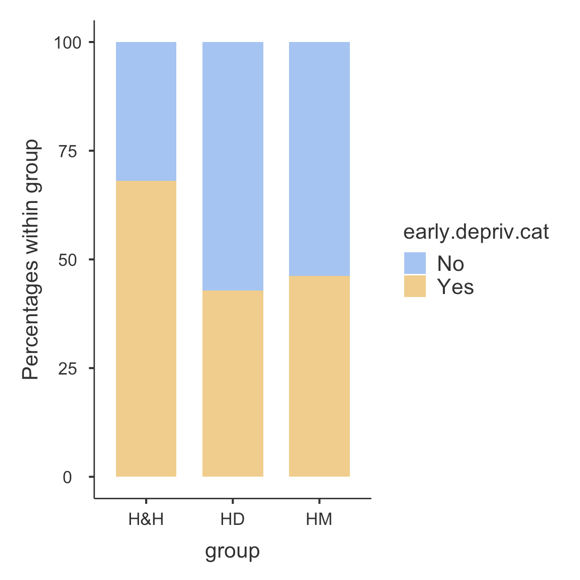


**13. Experiences of Early Material Deprivation (Continuous) x Group**

# Non-Parametric ANOVA

anovaNP(

formula = eemdq.total ~ group,

data = data3,

es = TRUE,

pairs = TRUE)

##

## ONE-WAY ANOVA (NON-PARAMETRIC)

##

## Kruskal-Wallis

## ───────────────────────────────────────────────────────────

## χ² df p ε²

## ───────────────────────────────────────────────────────────

## eemdq.total 14.07163 2 0.0008798 0.1108002

## ───────────────────────────────────────────────────────────

##

##

## DWASS-STEEL-CRITCHLOW-FLIGNER PAIRWISE COMPARISONS

##

## Pairwise comparisons - eemdq.total

## ────────────────────────────────────────

## W p

## ────────────────────────────────────────

## H&H HD -4.9358454 0.0014029

## H&H HM -3.9668846 0.0139500

## HD HM 0.8375295 0.8243088

## ────────────────────────────────────────

# One-Way ANOVA

anovaOneW(

formula = eemdq.total ~ group,

data = data3,

desc = TRUE,

descPlot = TRUE,

norm = TRUE,

qq = FALSE,

eqv = TRUE,

phTest = FALSE)

## ONE-WAY ANOVA

##

## One-Way ANOVA (Welch's)

## ───────────────────────────────────────────────────────────

## F df1 df2 p

## ───────────────────────────────────────────────────────────

## eemdq.total 8.684791 2 80.31422 0.0003850

## ───────────────────────────────────────────────────────────

##

##

## Group Descriptives

## ────────────────────────────────────────────────────────────────────

## group N Mean SD SE

## ────────────────────────────────────────────────────────────────────

## eemdq.total H&H 47 18.574468 12.892102 1.880506

## HD 42 9.238095 7.941277 1.225366

## HM 39 11.102564 10.708567 1.714743

## ────────────────────────────────────────────────────────────────────

##

##

## ASSUMPTION CHECKS

##

## Normality Test (Shapiro-Wilk)

## ─────────────────────────────────────────

## W p

## ─────────────────────────────────────────

## eemdq.total 0.9624533 0.0013031

## ─────────────────────────────────────────

## Note. A low p-value suggests a

## violation of the assumption of

## normality

##

##

## Homogeneity of Variances Test (Levene's)

## ──────────────────────────────────────────────────────

## F df1 df2 p

## ──────────────────────────────────────────────────────

## eemdq.total 8.712832 2 125 0.0002868

## ──────────────────────────────────────────────────────


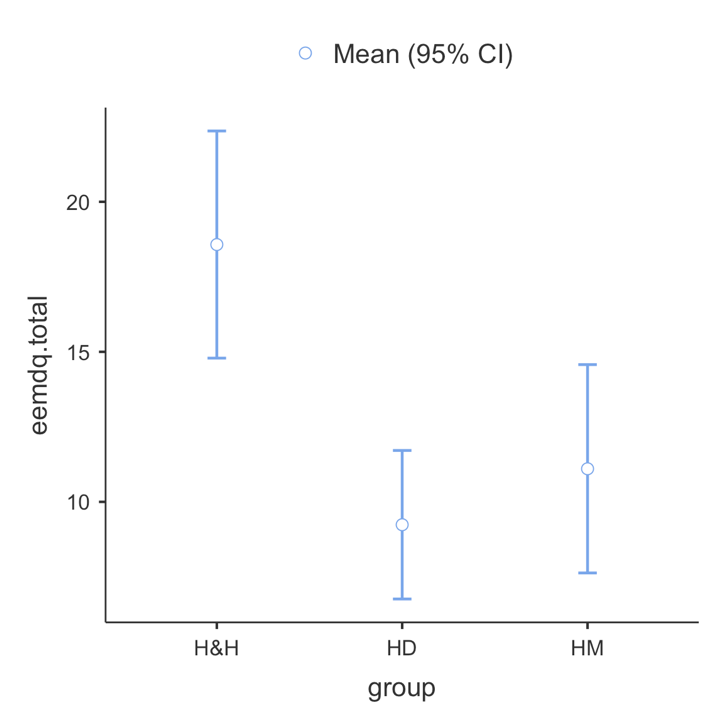


**14. Hypothesis 1**

Individuals experiencing hoarding and homelessness will report significantly higher scores of fear of material deprivation compared to those experiencing hoarding only or homelessness only.

# Factorial ANOVA

anovaRM(

data = data3,

rm = list(

list(

label="BAH",

levels=c("Fear MD", "Harm Av", "Att Dist"))),

rmCells = list(

list(

measure="bah.fear.mat.depr",

cell="Fear MD"),

list(

measure="bah.harm.avoi",

cell="Harm Av"),

list(

measure="bah.att.dist",

cell="Att Dist")),

bs = group,

effectSize = "ges",

depLabel = "BAH",

rmTerms = ~ `BAH`,

bsTerms = ~ group,

spherTests = TRUE,

spherCorr = c("none", "GG"),

leveneTest = TRUE,

qq = FALSE,

postHoc = list(

c("BAH", "group"),

"group",

"BAH"),

emMeans = ~ `BAH`:group + group:`BAH`,

emmTables = TRUE,

emmPlotData = TRUE,

groupSumm = TRUE)

##

## FACTORIAL ANOVA

##

## Within Subjects Effects

## ─────────────────────────────────────────────────────────────────────────────────────────────────────────────────────────────

## Sphericity Correction Sum of Squares df Mean Square F p η²-G

## ─────────────────────────────────────────────────────────────────────────────────────────────────────────────────────────────

## BAH None 17657.555 2 8828.7774 62.322861 < .0000001 0.0934143

## Greenhouse-Geisser 17657.555 1.973910 8945.4731 62.322861 < .0000001 0.0934143

##

## BAH:group None 4386.776 4 1096.6941 7.741628 0.0000067 0.0249598

## Greenhouse-Geisser 4386.776 3.947819 1111.1898 7.741628 0.0000076 0.0249598

##

## Residual None 35698.809 252 141.6619

## Greenhouse-Geisser 35698.809 248.712604 143.5344

## ─────────────────────────────────────────────────────────────────────────────────────────────────────────────────────────────

## Note. Type 3 Sums of Squares

##

##

## Between Subjects Effects

## ───────────────────────────────────────────────────────────────────────────────────────────

## Sum of Squares df Mean Square F p η²-G

## ───────────────────────────────────────────────────────────────────────────────────────────

## group 40967.30 2 20483.648 19.02397 < .0000001 0.1929381

## Residual 135667.80 126 1076.729

## ───────────────────────────────────────────────────────────────────────────────────────────

## Note. Type 3 Sums of Squares

##

##

## ASSUMPTIONS

##

## Tests of Sphericity

## ────────────────────────────────────────────────────────────────────────────

## Mauchly's W p Greenhouse-Geisser ε Huynh-Feldt ε

## ────────────────────────────────────────────────────────────────────────────

## BAH 0.9867823 0.4353472 0.9869548 1.000000

## ────────────────────────────────────────────────────────────────────────────

##

##

## Homogeneity of Variances Test (Levene's)

## ─────────────────────────────────────────────────────────────

## F df1 df2 p

## ─────────────────────────────────────────────────────────────

## bah.fear.mat.depr 0.1223260 2 126 0.8849648

## bah.harm.avoi 4.4453247 2 126 0.0136298

## bah.att.dist 1.7593133 2 126 0.1763656

## ─────────────────────────────────────────────────────────────

##

##

##

##

## POST HOC TESTS

##

## Post Hoc Comparisons - BAH:group

## ──────────────────────────────────────────────────────────────────────────────────────────────────────────────────────

## BAH group BAH group Mean Difference SE df t p-tukey

## ──────────────────────────────────────────────────────────────────────────────────────────────────────────────────────

## Fear MD H&H - Fear MD HD 5.5118753 4.445509 126.0000 1.2398750 0.9459243

## - Fear MD HM 25.1518276 4.563010 126.0000 5.5121136 0.0000067

## - Harm Av H&H 11.3106383 2.590361 126.0000 4.3664333 0.0008531

## - Harm Av HD 30.6676893 4.401037 126.0000 6.9682871 < .0000001

## - Harm Av HM 33.5672122 4.515228 126.0000 7.4342234 < .0000001

## - Att Dist H&H -0.9148936 2.362115 126.0000 -0.3873196 0.9999849

## - Att Dist HD 7.7630381 4.563307 126.0000 1.7011869 0.7447291

## - Att Dist HM 27.6056738 4.689469 126.0000 5.8867374 0.0000012

## HD - Fear MD HM 19.6399523 4.658261 126.0000 4.2161556 0.0015120

## - Harm Av H&H 5.7987630 4.404839 126.0000 1.3164528 0.9246822

## - Harm Av HD 25.1558140 2.708164 126.0000 9.2888808 < .0000001

## - Harm Av HM 28.0553369 4.611466 126.0000 6.0838218 0.0000005

## - Att Dist H&H -6.4267689 4.553400 126.0000 -1.4114219 0.8915127

## - Att Dist HD 2.2511628 2.469538 126.0000 0.9115723 0.9919595

## - Att Dist HM 22.0937984 4.782202 126.0000 4.6200052 0.0003133

## HM - Harm Av H&H -13.8411893 4.523397 126.0000 -3.0599107 0.0651207

## - Harm Av HD 5.5158617 4.615839 126.0000 1.1949857 0.9562224

## - Harm Av HM 8.4153846 2.843655 126.0000 2.9593550 0.0848001

## - Att Dist H&H -26.0667212 4.668186 126.0000 -5.5839079 0.0000049

## - Att Dist HD -17.3887895 4.770809 126.0000 -3.6448307 0.0113182

## - Att Dist HM 2.4538462 2.593091 126.0000 0.9463017 0.9897059

## Harm Av H&H - Harm Av HD 19.3570510 4.359952 126.0000 4.4397390 0.0006415

## - Harm Av HM 22.2565739 4.475192 126.0000 4.9733230 0.0000723

## - Att Dist H&H -12.2255319 2.407269 126.0000 -5.0785897 0.0000461

## - Att Dist HD -3.5476002 4.523697 126.0000 -0.7842259 0.9971224

## - Att Dist HM 16.2950355 4.650933 126.0000 3.5036055 0.0178174

## HD - Harm Av HM 2.8995230 4.568610 126.0000 0.6346620 0.9993685

## - Att Dist H&H -31.5825829 4.509992 126.0000 -7.0028019 < .0000001

## - Att Dist HD -22.9046512 2.516746 126.0000 -9.1009000 < .0000001

## - Att Dist HM -3.0620155 4.740889 126.0000 -0.6458736 0.9992822

## HM - Att Dist H&H -34.4821058 4.621492 126.0000 -7.4612500 < .0000001

## - Att Dist HD -25.8041741 4.725128 126.0000 -5.4610524 0.0000085

## - Att Dist HM -5.9615385 2.642660 126.0000 -2.2558857 0.3769215

## Att Dist H&H - Att Dist HD 8.6779317 4.668477 126.0000 1.8588358 0.6429587

## - Att Dist HM 28.5205674 4.791871 126.0000 5.9518648 0.0000009

## HD - Att Dist HM 19.8426357 4.891899 126.0000 4.0562233 0.0027286

## ──────────────────────────────────────────────────────────────────────────────────────────────────────────────────────

##

##

## Post Hoc Comparisons - group

## ────────────────────────────────────────────────────────────────────────────────────────────

## group group Mean Difference SE df t p-tukey

## ────────────────────────────────────────────────────────────────────────────────────────────

## H&H - HD 11.18229 3.997888 126.0000 2.797049 0.0163310

## - HM 25.30966 4.103557 126.0000 6.167736 < .0000001

## HD - HM 14.12737 4.189217 126.0000 3.372317 0.0028292

## ────────────────────────────────────────────────────────────────────────────────────────────

##

##

##

## Post Hoc Comparisons - BAH

## ───────────────────────────────────────────────────────────────────────────────────────────────────

## BAH BAH Mean Difference SE df t p-tukey

## ───────────────────────────────────────────────────────────────────────────────────────────────────

## Fear MD - Harm Av 14.960612 1.568102 126.0000 9.5405858 < .0000001

## - Att Dist 1.263372 1.429931 126.0000 0.8835193 0.6516791

## Harm Av - Att Dist -13.697241 1.457265 126.0000 -9.3992760 < .0000001

## ───────────────────────────────────────────────────────────────────────────────────────────────────

##

##

## ESTIMATED MARGINAL MEANS

##

## BAH:GROUP

##

## Estimated Marginal Means - BAH:group

## ─────────────────────────────────────────────────────────────────────

## group BAH Mean SE Lower Upper

## ─────────────────────────────────────────────────────────────────────

## H&H Fear MD 57.37234 3.072801 51.29136 63.45332

## Harm Av 46.06170 3.013663 40.09775 52.02565

## Att Dist 58.28723 3.226920 51.90125 64.67321

## HD Fear MD 51.86047 3.212545 45.50293 58.21800

## Harm Av 26.70465 3.150717 20.46947 32.93983

## Att Dist 49.60930 3.373672 42.93290 56.28570

## HM Fear MD 32.22051 3.373270 25.54491 38.89611

## Harm Av 23.80513 3.308349 17.25800 30.35225

## Att Dist 29.76667 3.542459 22.75624 36.77709

## ─────────────────────────────────────────────────────────────────────

##

##

## GROUP:BAH

##

## Estimated Marginal Means - group:BAH

## ─────────────────────────────────────────────────────────────────────

## BAH group Mean SE Lower Upper

## ─────────────────────────────────────────────────────────────────────

## Fear MD H&H 57.37234 3.072801 51.29136 63.45332

## HD 51.86047 3.212545 45.50293 58.21800

## HM 32.22051 3.373270 25.54491 38.89611

## Harm Av H&H 46.06170 3.013663 40.09775 52.02565

## HD 26.70465 3.150717 20.46947 32.93983

## HM 23.80513 3.308349 17.25800 30.35225

## Att Dist H&H 58.28723 3.226920 51.90125 64.67321

## HD 49.60930 3.373672 42.93290 56.28570

## HM 29.76667 3.542459 22.75624 36.77709

## ─────────────────────────────────────────────────────────────────────

##

##

| 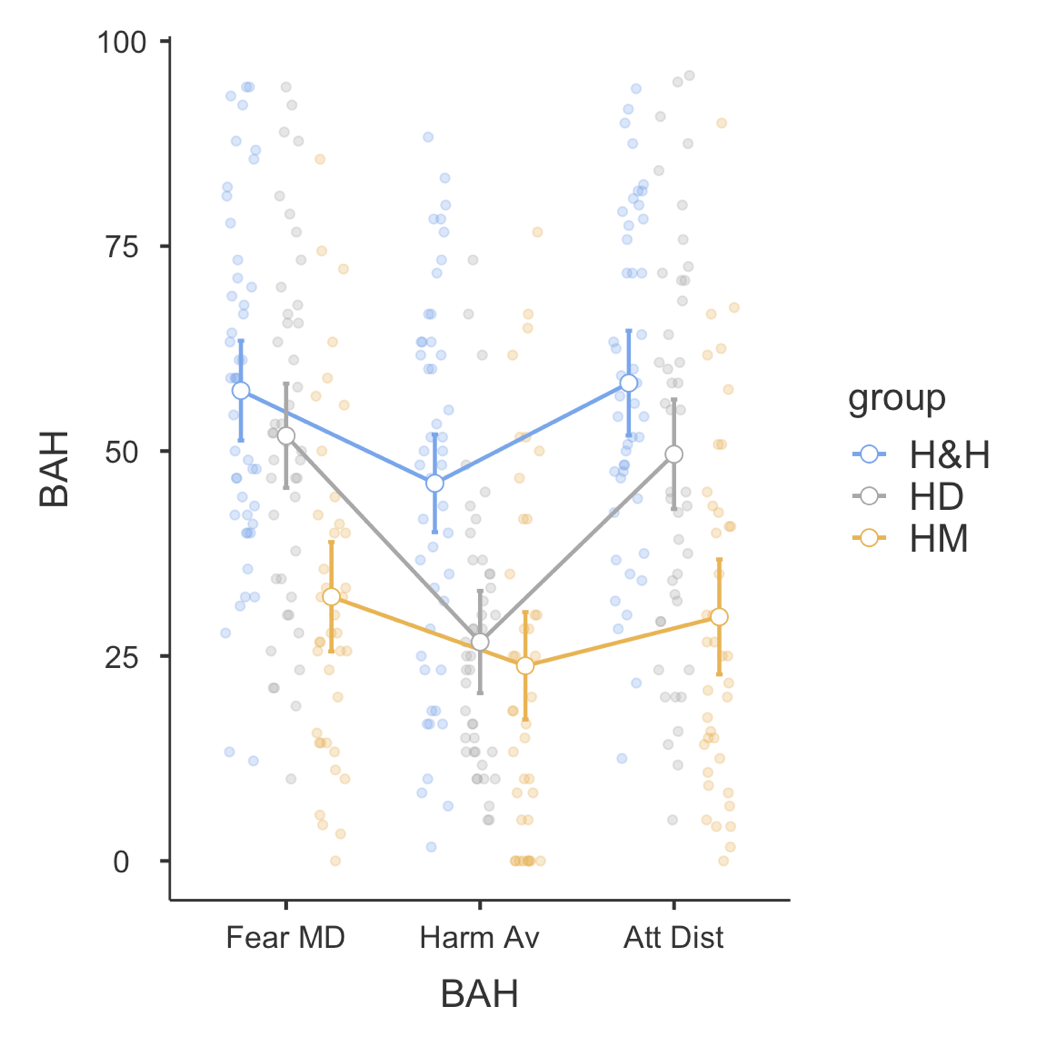 | 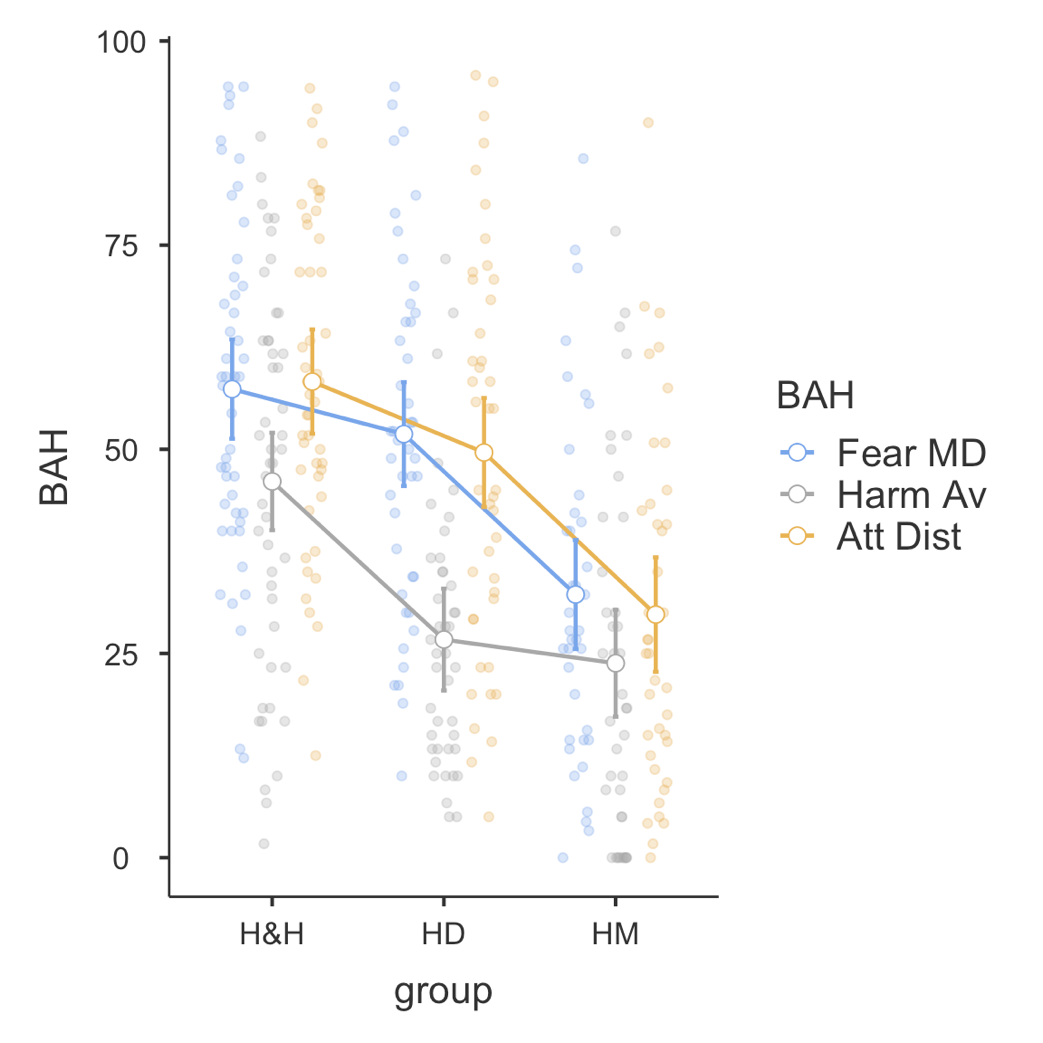 |
| --- | --- |

**14.1. Simple main effect 1: Fear of material deprivation**

# One-Way ANOVA

anovaOneW(

formula = bah.fear.mat.depr ~ group,

data = data3,

desc = TRUE,

descPlot = TRUE,

norm = TRUE,

eqv = TRUE,

phMethod = "tukey",

phTest = TRUE,

phFlag = TRUE)

## One-Way ANOVA (Welch's)

## ─────────────────────────────────────────────────────────────────

## F df1 df2 p

## ─────────────────────────────────────────────────────────────────

## bah.fear.mat.depr 16.62596 2 83.00152 0.0000008

## ─────────────────────────────────────────────────────────────────

##

##

## Group Descriptives

## ────────────────────────────────────────────────────────────────────────

## group N Mean SD SE

## ────────────────────────────────────────────────────────────────────────

## bah.fear.mat.depr H&H 47 57.37234 21.01205 3.064923

## HD 43 51.86047 21.50827 3.279981

## HM 39 32.22051 20.63302 3.303928

## ────────────────────────────────────────────────────────────────────────

##

##

## ASSUMPTION CHECKS

##

## Normality Test (Shapiro-Wilk)

## ───────────────────────────────────────────────

## W p

## ───────────────────────────────────────────────

## bah.fear.mat.depr 0.9843798 0.1458612

## ───────────────────────────────────────────────

## Note. A low p-value suggests a violation

## of the assumption of normality

##

##

## Homogeneity of Variances Test (Levene's)

## ─────────────────────────────────────────────────────────────

## F df1 df2 p

## ─────────────────────────────────────────────────────────────

## bah.fear.mat.depr 0.1223260 2 126 0.8849648

## ─────────────────────────────────────────────────────────────

##

##

## POST HOC TESTS

##

## Tukey Post-Hoc Test – bah.fear.mat.depr

## ─────────────────────────────────────────────────────────────────

## H&H HD HM

## ─────────────────────────────────────────────────────────────────

## H&H Mean difference — 5.511875 25.15183

## t-value — 1.239875 5.512114

## df — 126.0000 126.0000

## p-value — 0.4320424 0.0000006

##

## HD Mean difference — 19.63995

## t-value — 4.216156

## df — 126.0000

## p-value — 0.0001386

##

## HM Mean difference —

## t-value —

## df —

## p-value —

## ─────────────────────────────────────────────────────────────────

## Note. * p < .05, ** p < .01, *** p < .001


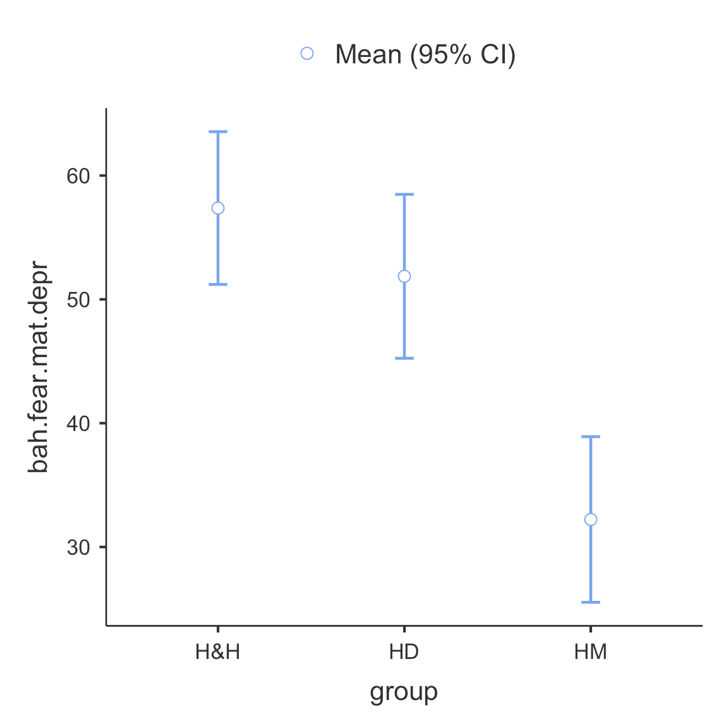


**14.2. Simple main effect 2: Harm avoidance**

# One-Way ANOVA - Games Howell Post-hoc test, as it violates homogeneity of variance.

anovaOneW(

formula = bah.harm.avoi ~ group,

data = data3,

desc = TRUE,

descPlot = TRUE,

norm = TRUE,

eqv = TRUE,

phMethod = "gamesHowell",

phTest = TRUE,

phFlag = TRUE)

## One-Way ANOVA (Welch's)

## ─────────────────────────────────────────────────────────────

## F df1 df2 p

## ─────────────────────────────────────────────────────────────

## bah.harm.avoi 13.51979 2 80.78345 0.0000086

## ─────────────────────────────────────────────────────────────

##

##

## Group Descriptives

## ────────────────────────────────────────────────────────────────────

## group N Mean SD SE

## ────────────────────────────────────────────────────────────────────

## bah.harm.avoi H&H 47 46.06170 23.06275 3.364047

## HD 43 26.70465 16.11719 2.457848

## HM 39 23.80513 22.00930 3.524309

## ────────────────────────────────────────────────────────────────────

##

##

## ASSUMPTION CHECKS

##

## Normality Test (Shapiro-Wilk)

## ───────────────────────────────────────────

## W p

## ───────────────────────────────────────────

## bah.harm.avoi 0.9813852 0.0736941

## ───────────────────────────────────────────

## Note. A low p-value suggests a

## violation of the assumption of

## normality

##

##

## Homogeneity of Variances Test (Levene's)

## ────────────────────────────────────────────────────────

## F df1 df2 p

## ────────────────────────────────────────────────────────

## bah.harm.avoi 4.445325 2 126 0.0136298

## ────────────────────────────────────────────────────────

##

##

## POST HOC TESTS

##

## Games-Howell Post-Hoc Test – bah.harm.avoi

## ─────────────────────────────────────────────────────────────────

## H&H HD HM

## ─────────────────────────────────────────────────────────────────

## H&H Mean difference — 19.35705 22.256574

## t-value — 4.646131 4.5681483

## df — 82.47773 82.33072

## p-value — 0.0000374 0.0000505

##

## HD Mean difference — 2.899523

## t-value — 0.6748230

## df — 69.15249

## p-value — 0.7788656

##

## HM Mean difference —

## t-value —

## df —

## p-value —

## ─────────────────────────────────────────────────────────────────

## Note. * p < .05, ** p < .01, *** p < .001


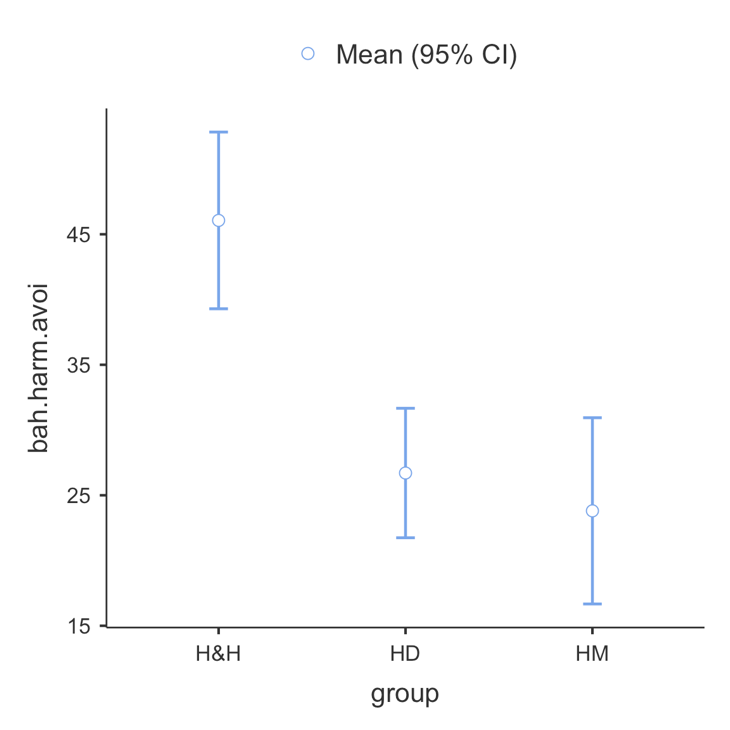


**14.3. Simple main effect 4: Attachment disturbance**

# One-Way ANOVA

anovaOneW(

formula = bah.att.dist ~ group,

data = data3,

desc = TRUE,

descPlot = TRUE,

norm = TRUE,

eqv = TRUE,

phMethod = "tukey",

phTest = TRUE,

phFlag = TRUE)

## ONE-WAY ANOVA

##

## One-Way ANOVA (Welch's)

## ─────────────────────────────────────────────────────────────

## F df1 df2 p

## ─────────────────────────────────────────────────────────────

## bah.att.dist 19.77708 2 81.50499 < .0000001

## ─────────────────────────────────────────────────────────────

##

##

## Group Descriptives

## ───────────────────────────────────────────────────────────────────

## group N Mean SD SE

## ───────────────────────────────────────────────────────────────────

## bah.att.dist H&H 47 58.28723 19.88601 2.900672

## HD 43 49.60930 24.57427 3.747541

## HM 39 29.76667 21.83155 3.495845

## ───────────────────────────────────────────────────────────────────

##

##

## ASSUMPTION CHECKS

##

## Normality Test (Shapiro-Wilk)

## ──────────────────────────────────────────

## W p

## ──────────────────────────────────────────

## bah.att.dist 0.9842044 0.1401891

## ──────────────────────────────────────────

## Note. A low p-value suggests a

## violation of the assumption of

## normality

##

##

## Homogeneity of Variances Test (Levene's)

## ───────────────────────────────────────────────────────

## F df1 df2 p

## ───────────────────────────────────────────────────────

## bah.att.dist 1.759313 2 126 0.1763656

## ───────────────────────────────────────────────────────

##

##

## POST HOC TESTS

##

## Tukey Post-Hoc Test – bah.att.dist

## ──────────────────────────────────────────────────────────────────

## H&H HD HM

## ──────────────────────────────────────────────────────────────────

## H&H Mean difference — 8.677932 28.52057

## t-value — 1.858836 5.951865

## df — 126.0000 126.0000

## p-value — 0.1550476 < .0000001

##

## HD Mean difference — 19.84264

## t-value — 4.056223

## df — 126.0000

## p-value — 0.0002548

##

## HM Mean difference —

## t-value —

## df —

## p-value —

## ──────────────────────────────────────────────────────────────────

## Note. * p < .05, ** p < .01, *** p < .001


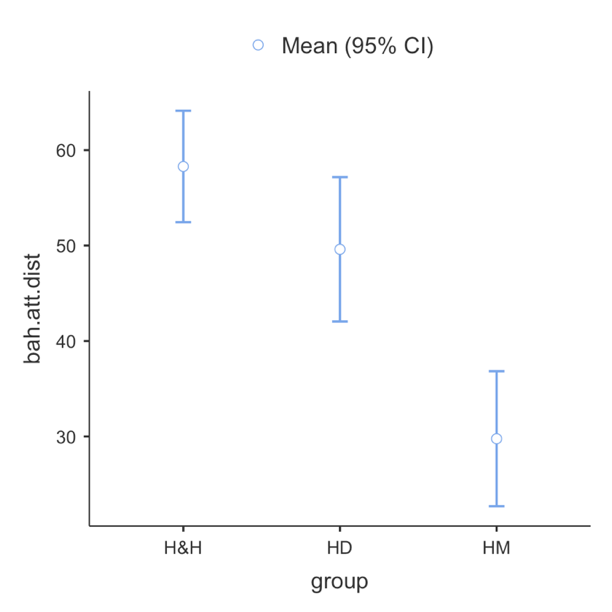


**15. Hypothesis 2**

Those with experiences of hoarding and homelessness will report a higher number of life events related to housing issues, compared with the hoarding-only and homelessness-only groups.

# Non-Parametric ANOVA

anovaNP(

formula = housing.total ~ group,

data = data3,

es = TRUE,

pairs = TRUE)

## ONE-WAY ANOVA (NON-PARAMETRIC)

##

## Kruskal-Wallis

## ──────────────────────────────────────────────────────────────

## χ² df p ε²

## ──────────────────────────────────────────────────────────────

## housing.total 42.18770 2 < .0000001 0.3321867

## ──────────────────────────────────────────────────────────────

##

##

## DWASS-STEEL-CRITCHLOW-FLIGNER PAIRWISE COMPARISONS

##

## Pairwise comparisons - housing.total

## ──────────────────────────────────────────

## W p

## ──────────────────────────────────────────

## H&H HD -8.60672364 < .0000001

## H&H HM -0.03107558 0.9997437

## HD HM 7.39144260 0.0000005

## ──────────────────────────────────────────

# Descriptives

descriptives(

formula = housing.total ~ group,

data = data3,

freq = TRUE,

desc = "rows",

missing = FALSE,

sum = TRUE)

## Descriptives

## ────────────────────────────────────────────────────────────────────────────────────────────

## group N Mean Median Sum SD Minimum Maximum

## ────────────────────────────────────────────────────────────────────────────────────────────

## housing.total H&H 47 2.89 2 136 1.89 0 8

## HD 42 0.69 0 29 1.07 0 4

## HM 39 3.00 3 117 2.32 0 9

## ────────────────────────────────────────────────────────────────────────────────────────────

# Ensure all values are non-negative

data3$housing.total <- pmax(data3$housing.total, 0)

# Create the plot

ggplot(data3, aes(x = group, y = housing.total)) +

geom_jitter(width = 0.2, height = 0, alpha = 0.6, colour = "grey") + # Adjust geom_jitter to prevent negative jittering

stat_summary(fun.data = **function**(y) {

med <- median(y)

conf.int <- quantile(y, probs = c(0.25, 0.75))

data.frame(y = med, ymin = pmax(conf.int[1], 0), ymax = conf.int[2]) # Ensure ymin is not less than 0

},

geom = "pointrange", shape = 21, colour = "black", fill = "white", size = 1) + # Medians with CIs

stat_summary(fun = median, geom = "line", aes(group = 1), colour = "black", size = 1) + # Connect medians with a line

stat_summary(fun.data = **function**(y) {

med <- median(y)

conf.int <- quantile(y, probs = c(0.25, 0.75))

data.frame(y = med, ymin = pmax(conf.int[1], 0), ymax = conf.int[2]) # Ensure ymin is not less than 0

}, geom = "errorbar", width = 0.2, colour = "black", size = 1) + # Horizontal lines at CI extremes

theme_light() + # Light theme

theme(

axis.title = element_text(size = 14), # Increase axis title font size

axis.text = element_text(size = 12), # Increase axis text font size

axis.title.x = element_text(margin = margin(t = 15)), # Increase distance between x-axis label and axis

axis.title.y = element_text(margin = margin(r = 15)), # Increase distance between y-axis label and axis

panel.border = element_blank(), # Remove plot border

panel.grid.major = element_line(colour = "grey90"), # Adjust grid lines if necessary

panel.grid.minor = element_line(colour = "grey95")

) +

labs(x = "Groups", y = "Negative experiences of housing (median - 95%CI)") # Labels


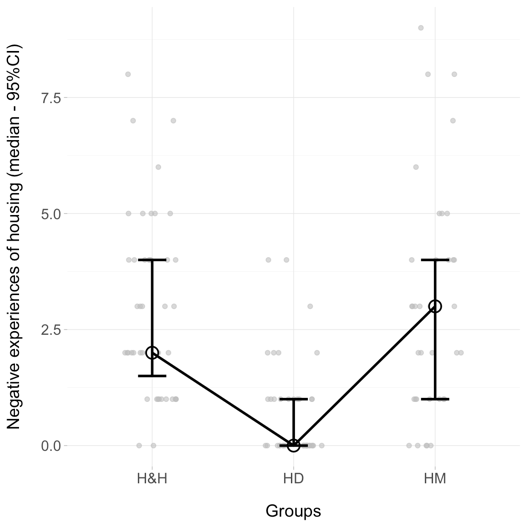


## Appendix II: Pre-registration of study hypotheses

## Appendix III: Blind eligibility rating of cases’ response patterns

Dear research team,

Unfortunately, some participants responded to the research questionnaire carelessly. To increase the reliability of our data, I used an algorithm in R (see details [here](https://cran.r-project.org/web/packages/responsePatterns/responsePatterns.pdf)) that employs autocorrelation to identify cases of indiscriminate responding. After identifying those cases, the algorithm produces plots that represent the patterns of responses of each case classified as *potentially unreliable*. With those plots, researchers can make a decision on whether to include or exclude those cases from the dataset based on visual inspection.

The best way to do this is with all members of our research team participating blindly to decrease bias in decision-making and ensure reliability through higher levels of agreement.

Each plot below represents a case identified by the algorithm as potentially presenting unreliable responses. On the y-axis is a Likert-type response scale, standardised from 0 to 100 (instead of 0 to 3 for the PHQ and GAD, for example). The x-axis indicates where each scale begins and ends.

- PHQ: Patient Health Questionnaire
- GAD: Generalised Anxiety Disorder
- WSAJ: Work and social adjustment scale
- BAH: The Beliefs about Hoarding Questionnaire
- SIR: The Savings Inventory-Revised
- INQ: The Interpersonal Needs Questionnaire
- EEMDQ: Experience of Early Material Deprivation Questionnaire
- MDHB: Material deprivation and hoarding beliefs
- HOM_SEV: Experiences of Homelessness - Severity of Exposure

**The ideal plot** will present variation of responses across items and scales.

**The unreliable plot** will present an excess of repetition of responses across items and scales.

For each plot, please vote on whether the case should be included or excluded from the dataset, basing your decision on visual inspection and the explanations provided above.

IMPORTANT: Given the broad definition of homelessness and the inclusion of items mostly related to the effects of rough sleeping in our HOM_SEV scale, it is anticipated that some participants may exhibit repetition of responses. As some people did not experience early material deprivation, the same applies to EEMDQ and MDHB. Therefore, consider the entire plot and pattern of responses when making your judgment and decision.

We provide some examples below.


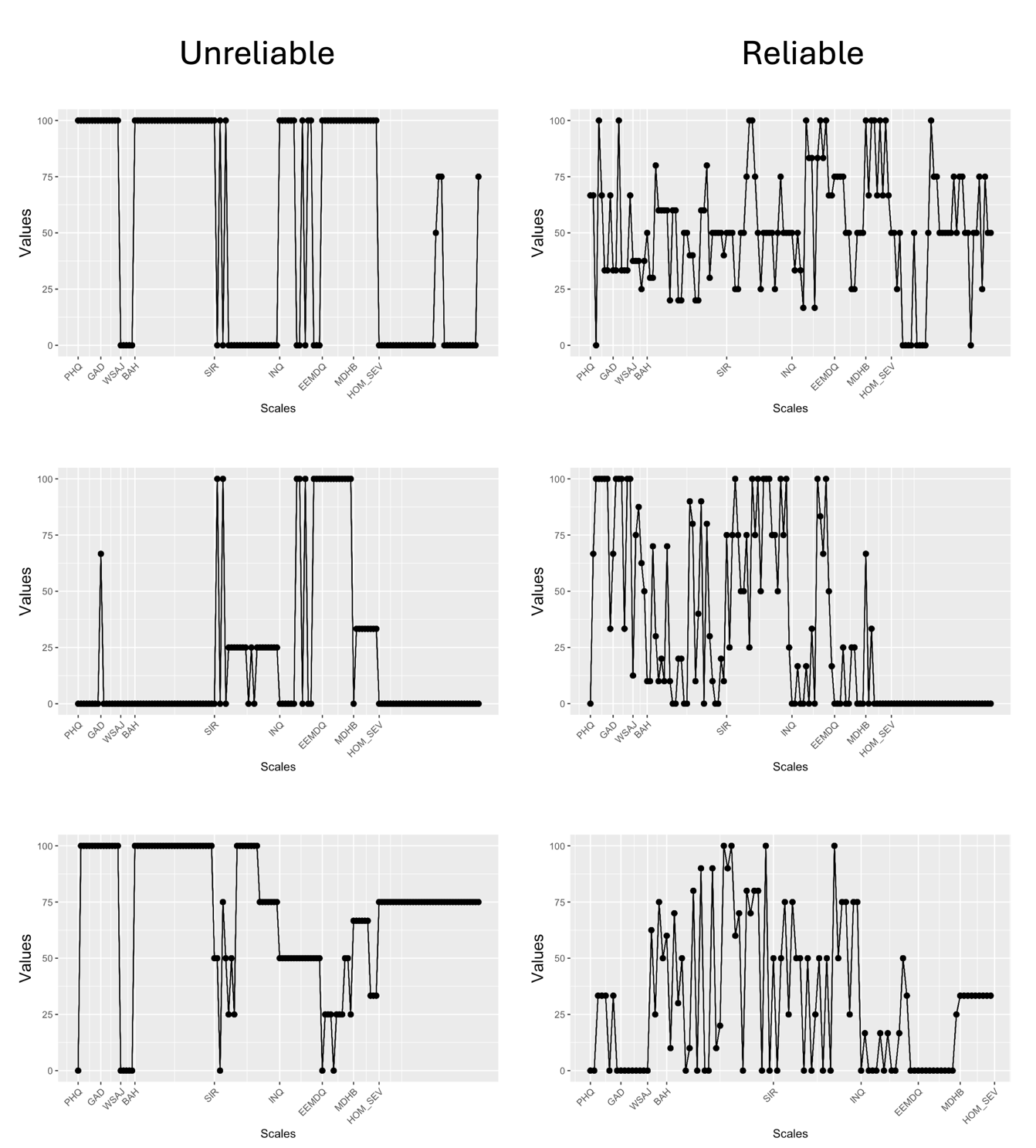


Your name: ______________________________

| 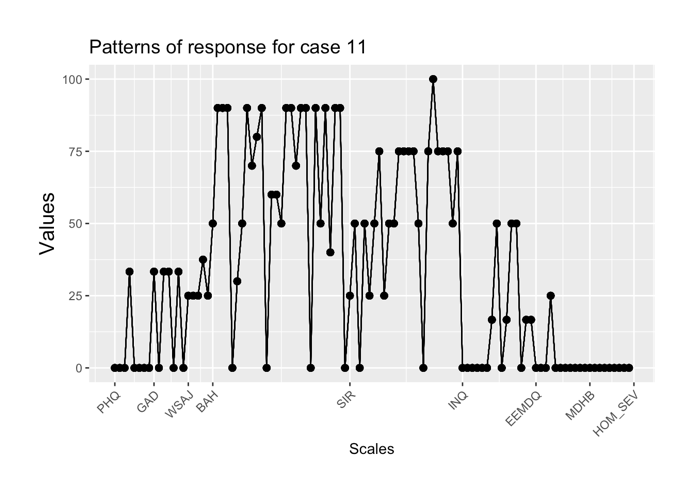 | ( ) Include in the dataset  ( ) Exclude from the dataset  How confident do you feel about your decision regarding this case?_____  0 = Not confident at all  10 = Extremely confident |
| --- | --- |

| 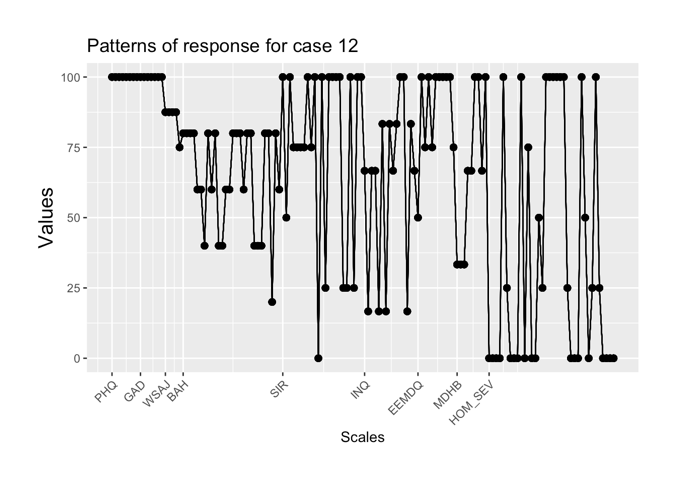 | ( ) Include in the dataset  ( ) Exclude from the dataset  How confident do you feel about your decision regarding this case?_____  0 = Not confident at all  10 = Extremely confident |
| --- | --- |

| 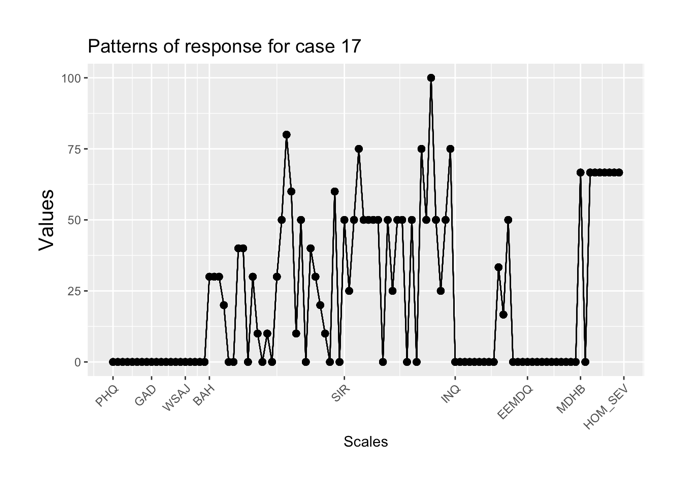 | ( ) Include in the dataset  ( ) Exclude from the dataset  How confident do you feel about your decision regarding this case?_____  0 = Not confident at all  10 = Extremely confident |
| --- | --- |

| 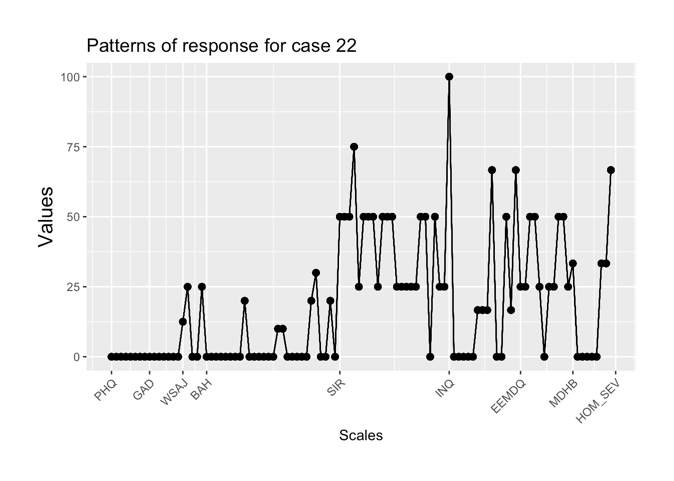 | ( ) Include in the dataset  ( ) Exclude from the dataset  How confident do you feel about your decision regarding this case?_____  0 = Not confident at all  10 = Extremely confident |
| --- | --- |

| 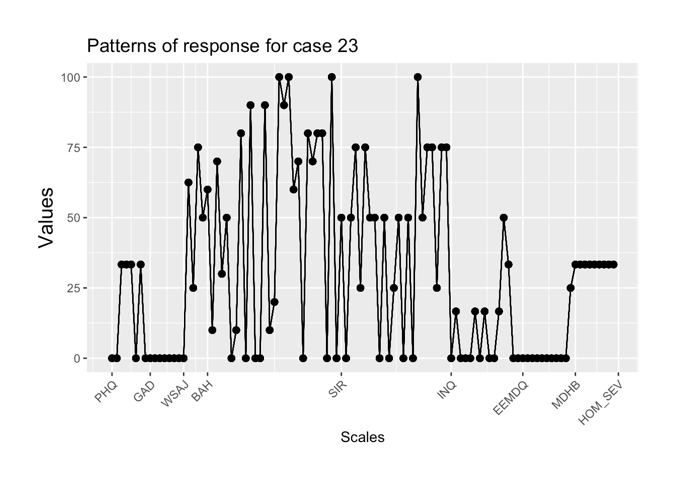 | ( ) Include in the dataset  ( ) Exclude from the dataset  How confident do you feel about your decision regarding this case?_____  0 = Not confident at all  10 = Extremely confident |
| --- | --- |

| 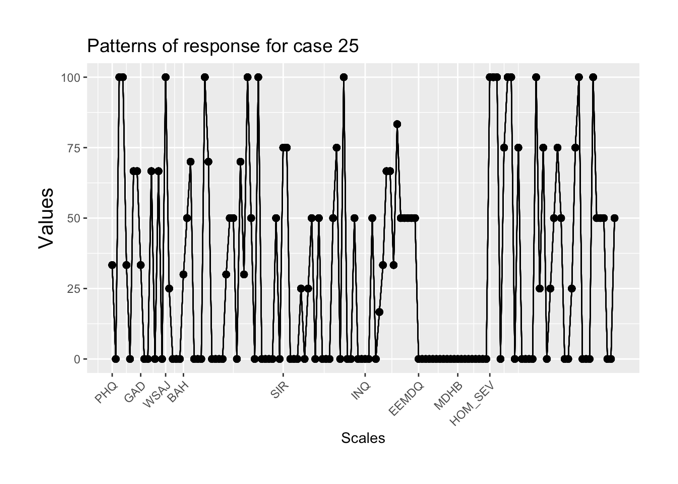 | ( ) Include in the dataset  ( ) Exclude from the dataset  How confident do you feel about your decision regarding this case?_____  0 = Not confident at all  10 = Extremely confident |
| --- | --- |

| 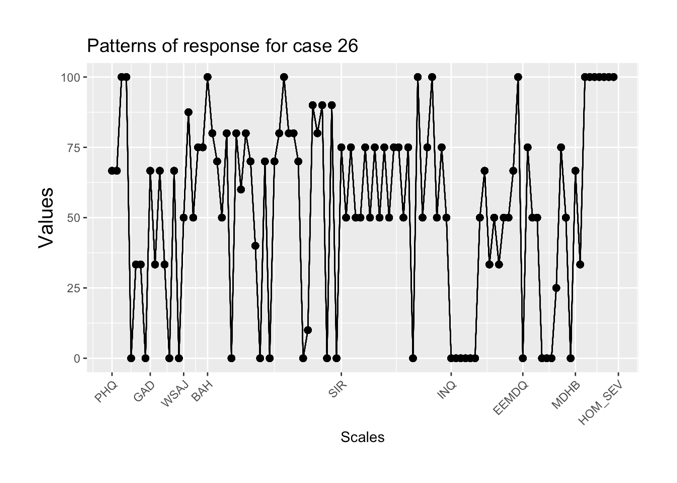 | ( ) Include in the dataset  ( ) Exclude from the dataset  How confident do you feel about your decision regarding this case?_____  0 = Not confident at all  10 = Extremely confident |
| --- | --- |

| 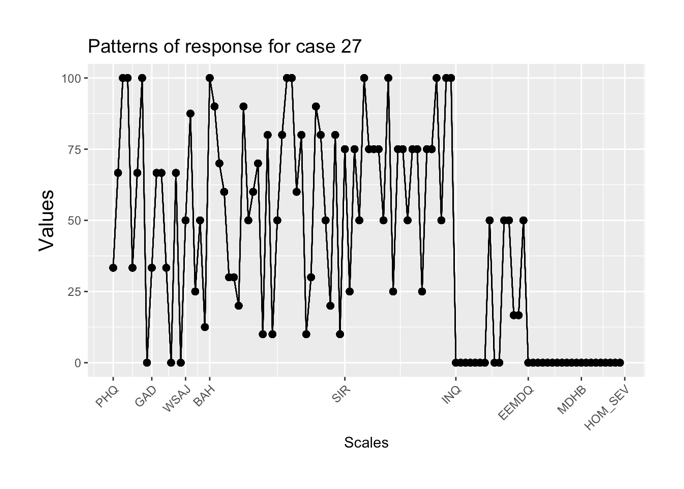 | ( ) Include in the dataset  ( ) Exclude from the dataset  How confident do you feel about your decision regarding this case?_____  0 = Not confident at all  10 = Extremely confident |
| --- | --- |

| 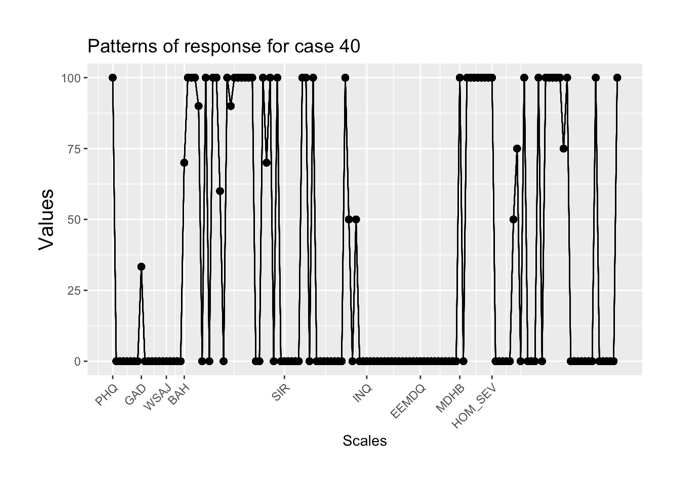 | ( ) Include in the dataset  ( ) Exclude from the dataset  How confident do you feel about your decision regarding this case?_____  0 = Not confident at all  10 = Extremely confident |
| --- | --- |

| 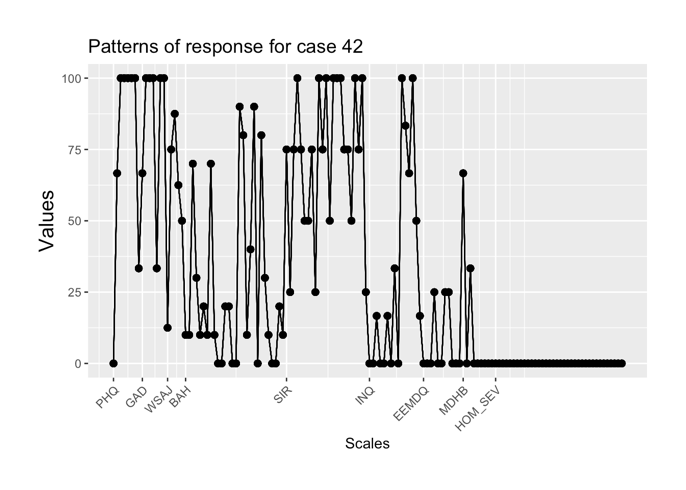 | ( ) Include in the dataset  ( ) Exclude from the dataset  How confident do you feel about your decision regarding this case?_____  0 = Not confident at all  10 = Extremely confident |
| --- | --- |

| 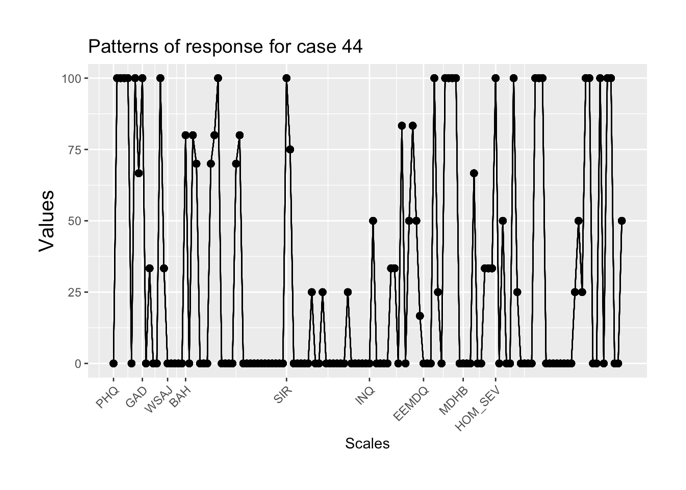 | ( ) Include in the dataset  ( ) Exclude from the dataset  How confident do you feel about your decision regarding this case?_____  0 = Not confident at all  10 = Extremely confident |
| --- | --- |

| 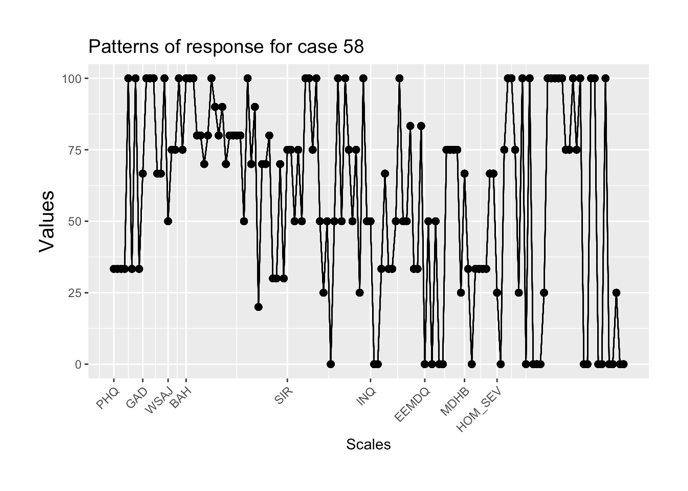 | ( ) Include in the dataset  ( ) Exclude from the dataset  How confident do you feel about your decision regarding this case?_____  0 = Not confident at all  10 = Extremely confident |
| --- | --- |

| 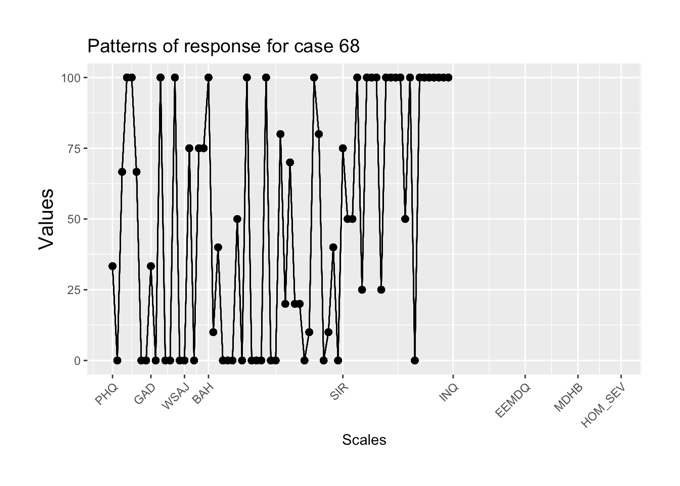 | ( ) Include in the dataset  ( ) Exclude from the dataset  How confident do you feel about your decision regarding this case?_____  0 = Not confident at all  10 = Extremely confident |
| --- | --- |

| 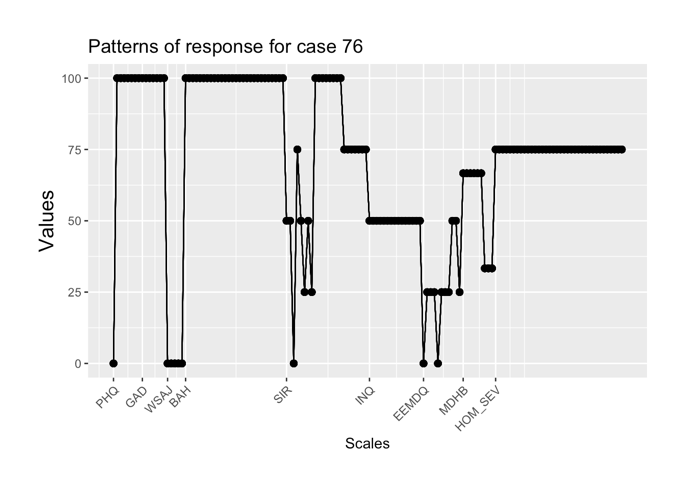 | ( ) Include in the dataset  ( ) Exclude from the dataset  How confident do you feel about your decision regarding this case?_____  0 = Not confident at all  10 = Extremely confident |
| --- | --- |

| 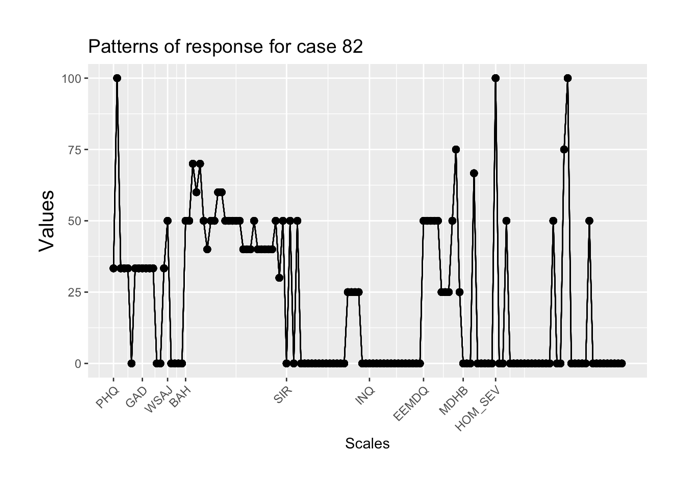 | ( ) Include in the dataset  ( ) Exclude from the dataset  How confident do you feel about your decision regarding this case?_____  0 = Not confident at all  10 = Extremely confident |
| --- | --- |

| 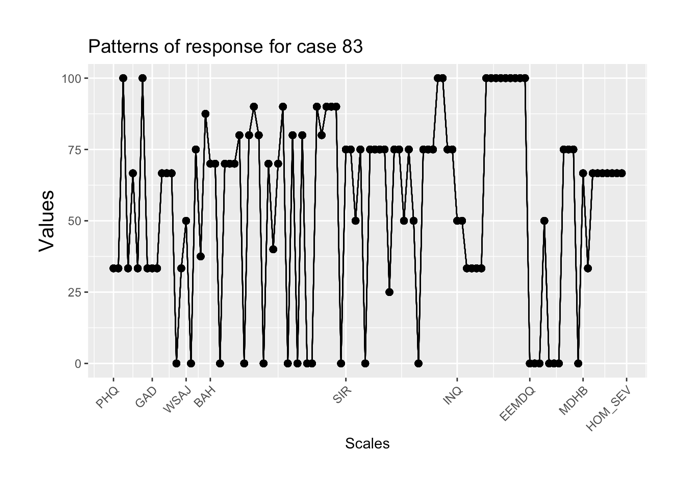 | ( ) Include in the dataset  ( ) Exclude from the dataset  How confident do you feel about your decision regarding this case?_____  0 = Not confident at all  10 = Extremely confident |
| --- | --- |

| 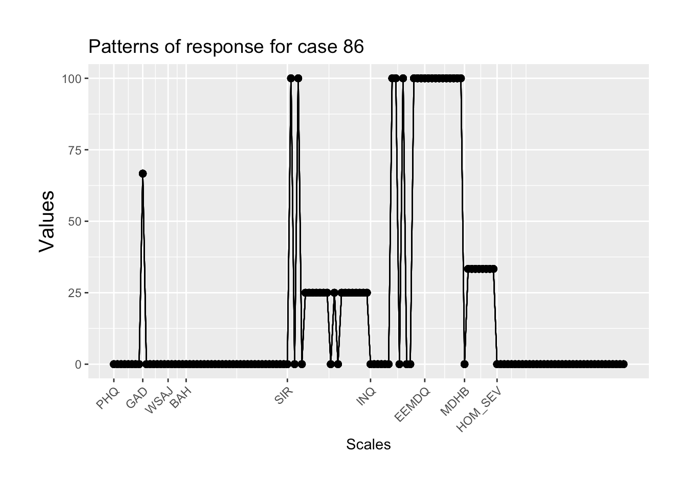 | ( ) Include in the dataset  ( ) Exclude from the dataset  How confident do you feel about your decision regarding this case?_____  0 = Not confident at all  10 = Extremely confident |
| --- | --- |

| 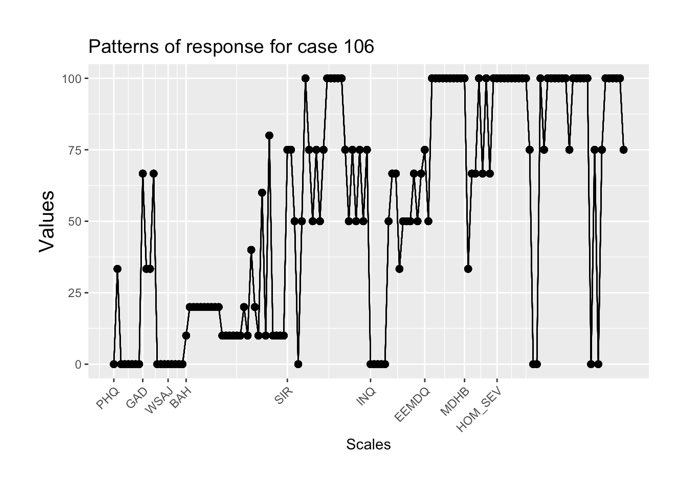 | ( ) Include in the dataset  ( ) Exclude from the dataset  How confident do you feel about your decision regarding this case?_____  0 = Not confident at all  10 = Extremely confident |
| --- | --- |

| 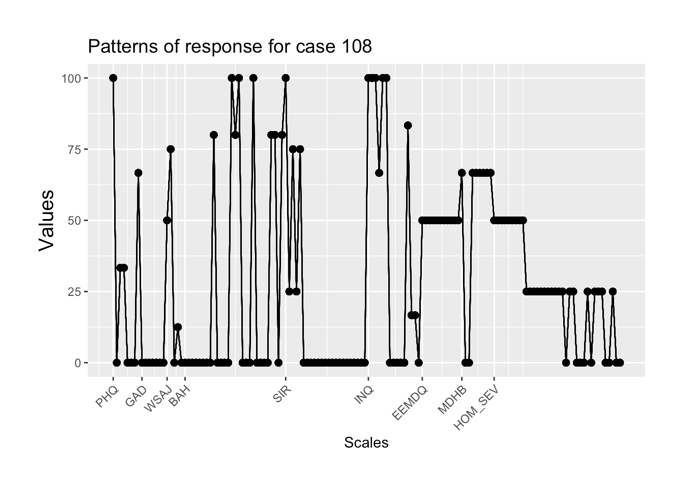 | ( ) Include in the dataset  ( ) Exclude from the dataset  How confident do you feel about your decision regarding this case?_____  0 = Not confident at all  10 = Extremely confident |
| --- | --- |

| 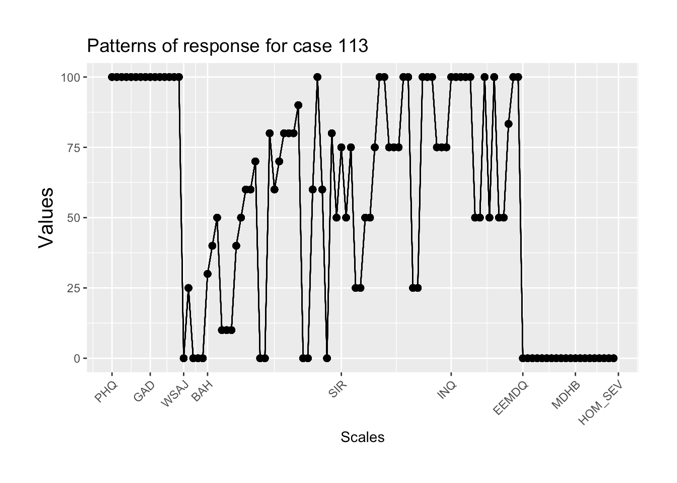 | ( ) Include in the dataset  ( ) Exclude from the dataset  How confident do you feel about your decision regarding this case?_____  0 = Not confident at all  10 = Extremely confident |
| --- | --- |

| 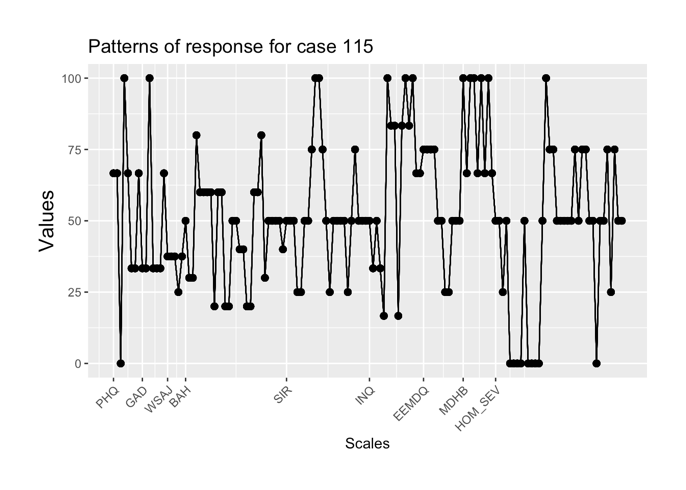 | ( ) Include in the dataset  ( ) Exclude from the dataset  How confident do you feel about your decision regarding this case?_____  0 = Not confident at all  10 = Extremely confident |
| --- | --- |

| 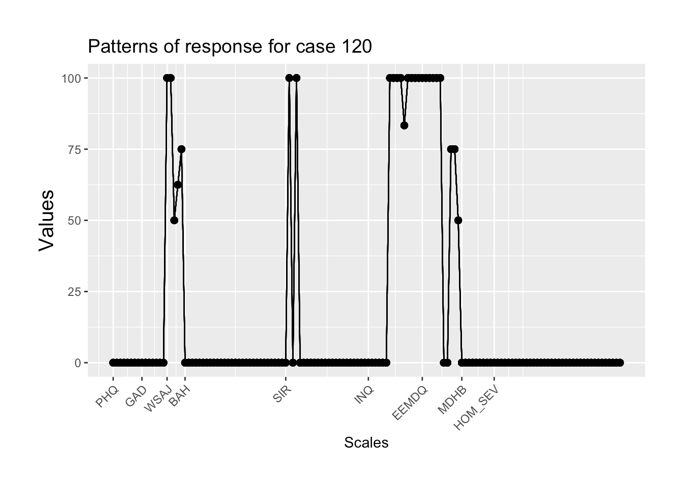 | ( ) Include in the dataset  ( ) Exclude from the dataset  How confident do you feel about your decision regarding this case?_____  0 = Not confident at all  10 = Extremely confident |
| --- | --- |

| 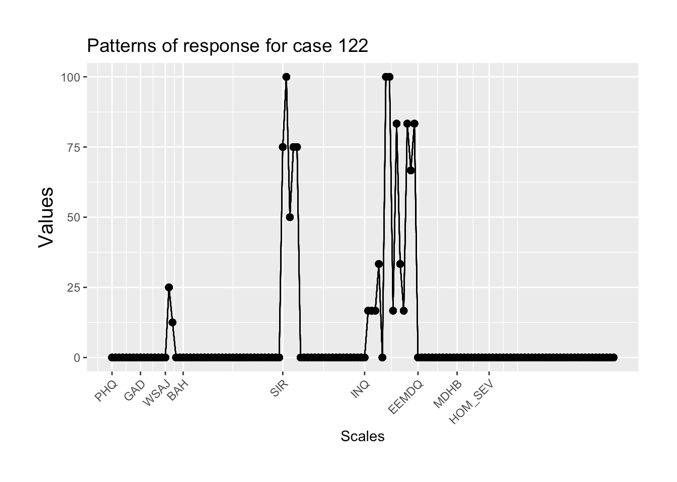 | ( ) Include in the dataset  ( ) Exclude from the dataset  How confident do you feel about your decision regarding this case?_____  0 = Not confident at all  10 = Extremely confident |
| --- | --- |

| 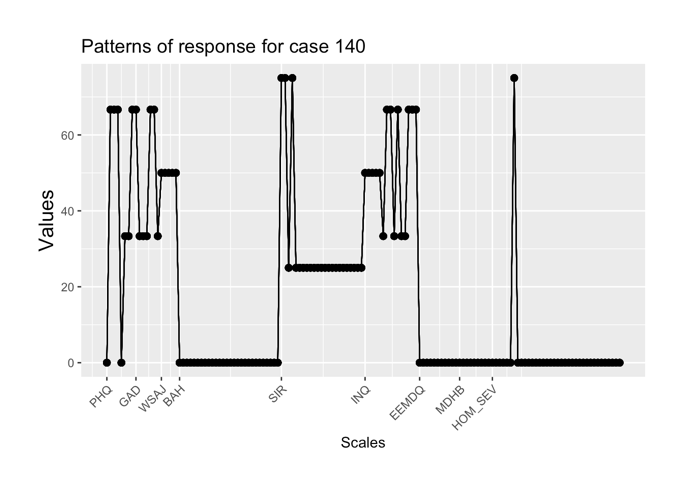 | ( ) Include in the dataset  ( ) Exclude from the dataset  How confident do you feel about your decision regarding this case?_____  0 = Not confident at all  10 = Extremely confident |
| --- | --- |

| 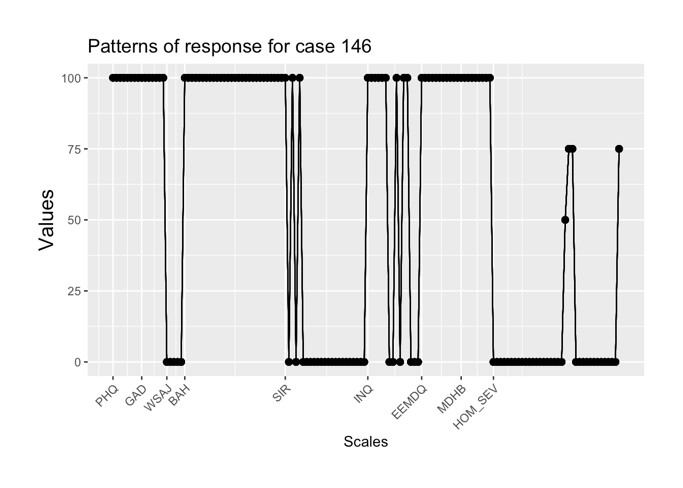 | ( ) Include in the dataset  ( ) Exclude from the dataset  How confident do you feel about your decision regarding this case?_____  0 = Not confident at all  10 = Extremely confident |
| --- | --- |

**Final results from blind rating (retrieved from Appendix I, R Script 3)**

The plots below represent the decisions made by each researcher (AC, FS, PS, SA, and TZ) on whether including or excluding cases from the final dataset. The colours indicate those decisions, and the scale at the bottom represents the researchers’ self-reported level of confidence in their decision (ranging from 0 = Not confident at all to 10 = Extremely confident).


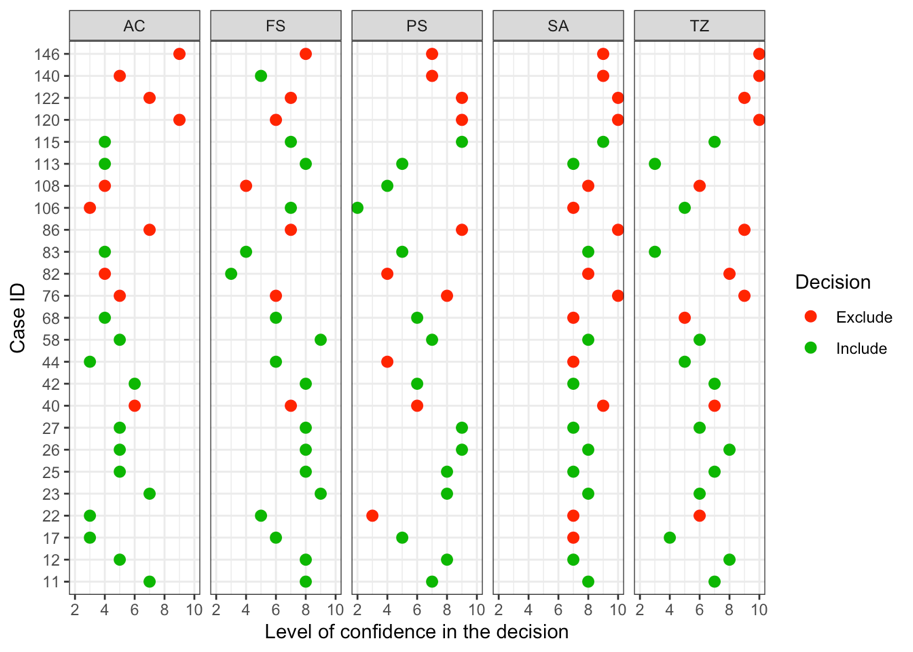


| **Case** | **Final Decision** | **Agreement Rate** | **Confidence Level** |
| --- | --- | --- | --- |
| **11** | Include | 100 | 74 |
| **12** | Include | 100 | 72 |
| **17** | Include | 80 | 50 |
| **22** | Exclude | 60 | 48 |
| **23** | Include | 100 | 76 |
| **25** | Include | 100 | 70 |
| **26** | Include | 100 | 76 |
| **27** | Include | 100 | 70 |
| **40** | Exclude | 100 | 70 |
| **42** | Include | 100 | 68 |
| **44** | Include | 60 | 50 |
| **58** | Include | 100 | 70 |
| **68** | Include | 60 | 56 |
| **76** | Exclude | 100 | 76 |
| **82** | Exclude | 80 | 54 |
| **83** | Include | 100 | 48 |
| **86** | Exclude | 100 | 84 |
| **106** | Include | 60 | 48 |
| **108** | Exclude | 80 | 52 |
| **113** | Include | 100 | 54 |
| **115** | Include | 100 | 72 |
| **120** | Exclude | 100 | 88 |
| **122** | Exclude | 100 | 84 |
| **140** | Exclude | 80 | 72 |
| **146** | Exclude | 100 | 86 |

| Total Number of Cases | 25 |
| --- | --- |
| Number of Cases Included | 15 |
| Number of Cases Excluded | 10 |
| Overall Confidence for Included Cases | 63.6 |
| Overall Confidence for Excluded Cases | 71.4 |
| Overall Agreement for Included Cases | 90.6 |
| Overall Agreement for Excluded Cases | 90 |

## Appendix IV: Full descriptive statistics of the sample

| *Table L1*. Descriptive statistics of gender by group | | | | | | | | | | | |
| --- | --- | --- | --- | --- | --- | --- | --- | --- | --- | --- | --- |
|  | | | | **Groups** | | | | | |  | |
| **Gender** | |  | | **H&H** | | **HD** | | **HM** | | **Total** | |
| Female |  | Observed |  | 19 |  | 37 |  | 9 |  | 65 |  |
|  | | % within row |  | 29.2 % |  | 56.9 % |  | 13.8 % |  | 100.0 % |  |
|  | | % within column |  | 40.4 % |  | 86.0 % |  | 23.1 % |  | 50.4 % |  |
| Male |  | Observed |  | 26 |  | 4 |  | 30 |  | 60 |  |
|  | | % within row |  | 43.3 % |  | 6.7 % |  | 50.0 % |  | 100.0 % |  |
|  | | % within column |  | 55.3 % |  | 9.3 % |  | 76.9 % |  | 46.5 % |  |
| Non-binary |  | Observed |  | 2 |  | 2 |  | 0 |  | 4 |  |
|  | | % within row |  | 50.0 % |  | 50.0 % |  | 0.0 % |  | 100.0 % |  |
|  | | % within column |  | 4.3 % |  | 4.7 % |  | 0.0 % |  | 3.1 % |  |
| Total |  | Observed |  | 47 |  | 43 |  | 39 |  | 129 |  |
|  | | % within row |  | 36.4 % |  | 33.3 % |  | 30.2 % |  | 100.0 % |  |
|  | | % within column |  | 100.0 % |  | 100.0 % |  | 100.0 % |  | 100.0 % |  |

| *Table L2.* Descriptive statistics of gender by group | | | | | | | | | | | |
| --- | --- | --- | --- | --- | --- | --- | --- | --- | --- | --- | --- |
|  | | | | **Groups** | | | | | |  | |
| **Sexual orientation** | |  | | **H&H** | | **HD** | | **HM** | | **Total** | |
| Asexual |  | Observed |  | 0 |  | 2 |  | 1 |  | 3 |  |
|  | | % within row |  | 0.0 % |  | 66.7 % |  | 33.3 % |  | 100.0 % |  |
|  | | % within column |  | 0.0 % |  | 4.8 % |  | 2.6 % |  | 2.4 % |  |
| Bisexual |  | Observed |  | 5 |  | 2 |  | 3 |  | 10 |  |
|  | | % within row |  | 50.0 % |  | 20.0 % |  | 30.0 % |  | 100.0 % |  |
|  | | % within column |  | 10.9 % |  | 4.8 % |  | 7.7 % |  | 7.9 % |  |
| Heterosexual |  | Observed |  | 34 |  | 35 |  | 33 |  | 102 |  |
|  | | % within row |  | 33.3 % |  | 34.3 % |  | 32.4 % |  | 100.0 % |  |
|  | | % within column |  | 73.9 % |  | 83.3 % |  | 84.6 % |  | 80.3 % |  |
| Homosexual |  | Observed |  | 4 |  | 1 |  | 2 |  | 7 |  |
|  | | % within row |  | 57.1 % |  | 14.3 % |  | 28.6 % |  | 100.0 % |  |
|  | | % within column |  | 8.7 % |  | 2.4 % |  | 5.1 % |  | 5.5 % |  |
| Pansexual |  | Observed |  | 1 |  | 0 |  | 0 |  | 1 |  |
|  | | % within row |  | 100.0 % |  | 0.0 % |  | 0.0 % |  | 100.0 % |  |
|  | | % within column |  | 2.2 % |  | 0.0 % |  | 0.0 % |  | 0.8 % |  |
| Prefer not to say |  | Observed |  | 2 |  | 2 |  | 0 |  | 4 |  |
|  | | % within row |  | 50.0 % |  | 50.0 % |  | 0.0 % |  | 100.0 % |  |
|  | | % within column |  | 4.3 % |  | 4.8 % |  | 0.0 % |  | 3.1 % |  |
| Total |  | Observed |  | 46 |  | 42 |  | 39 |  | 127 |  |
|  | | % within row |  | 36.2 % |  | 33.1 % |  | 30.7 % |  | 100.0 % |  |
|  | | % within column |  | 100.0 % |  | 100.0 % |  | 100.0 % |  | 100.0 % |  |

| *Table L3*. Descriptive statistics of ethnicity by group | | | | | | | | | | | |
| --- | --- | --- | --- | --- | --- | --- | --- | --- | --- | --- | --- |
|  | | | | **Groups** | | | | | |  | |
| **Ethnicity** |  | | | **H&H** | | **HD** | | **HM** | | **Total** | |
| Arab |  | Observed |  | 1 |  | 0 |  | 1 |  | 2 |  |
|  | % within row | |  | 50.0 % |  | 0.0 % |  | 50.0 % |  | 100.0 % |  |
|  | % within column | |  | 2.1 % |  | 0.0 % |  | 2.6 % |  | 1.6 % |  |
| Bangladeshi or British Bangladeshi |  | Observed |  | 1 |  | 0 |  | 0 |  | 1 |  |
|  | % within row | |  | 100.0 % |  | 0.0 % |  | 0.0 % |  | 100.0 % |  |
|  | % within column | |  | 2.1 % |  | 0.0 % |  | 0.0 % |  | 0.8 % |  |
| Black (Black British / Black Caribbean / Black African / Black other) |  | Observed |  | 2 |  | 1 |  | 2 |  | 5 |  |
|  | % within row | |  | 40.0 % |  | 20.0 % |  | 40.0 % |  | 100.0 % |  |
|  | % within column | |  | 4.3 % |  | 2.3 % |  | 5.1 % |  | 3.9 % |  |
| Mixed (White and Black Caribbean / White and Black African / White and Asian) |  | Observed |  | 6 |  | 1 |  | 1 |  | 8 |  |
|  | % within row | |  | 75.0 % |  | 12.5 % |  | 12.5 % |  | 100.0 % |  |
|  | % within column | |  | 12.8 % |  | 2.3 % |  | 2.6 % |  | 6.2 % |  |
| Other |  | Observed |  | 3 |  | 4 |  | 2 |  | 9 |  |
|  | % within row | |  | 33.3 % |  | 44.4 % |  | 22.2 % |  | 100.0 % |  |
|  | % within column | |  | 6.4 % |  | 9.3 % |  | 5.1 % |  | 7.0 % |  |
| Other Asian background |  | Observed |  | 1 |  | 0 |  | 0 |  | 1 |  |
|  | % within row | |  | 100.0 % |  | 0.0 % |  | 0.0 % |  | 100.0 % |  |
|  | % within column | |  | 2.1 % |  | 0.0 % |  | 0.0 % |  | 0.8 % |  |
| Other Mixed / Multiple ethnic background |  | Observed |  | 4 |  | 2 |  | 1 |  | 7 |  |
|  | % within row | |  | 57.1 % |  | 28.6 % |  | 14.3 % |  | 100.0 % |  |
|  | % within column | |  | 8.5 % |  | 4.7 % |  | 2.6 % |  | 5.4 % |  |
| Pakistani or British Pakistani |  | Observed |  | 0 |  | 2 |  | 1 |  | 3 |  |
|  | % within row | |  | 0.0 % |  | 66.7 % |  | 33.3 % |  | 100.0 % |  |
|  | % within column | |  | 0.0 % |  | 4.7 % |  | 2.6 % |  | 2.3 % |  |
| Prefer not to say |  | Observed |  | 1 |  | 0 |  | 0 |  | 1 |  |
|  | % within row | |  | 100.0 % |  | 0.0 % |  | 0.0 % |  | 100.0 % |  |
|  | % within column | |  | 2.1 % |  | 0.0 % |  | 0.0 % |  | 0.8 % |  |
| Unsure |  | Observed |  | 1 |  | 0 |  | 0 |  | 1 |  |
|  | % within row | |  | 100.0 % |  | 0.0 % |  | 0.0 % |  | 100.0 % |  |
|  | % within column | |  | 2.1 % |  | 0.0 % |  | 0.0 % |  | 0.8 % |  |
| White (English / Welsh / Scottish / Northern Irish / British) |  | Observed |  | 24 |  | 32 |  | 31 |  | 87 |  |
|  | % within row | |  | 27.6 % |  | 36.8 % |  | 35.6 % |  | 100.0 % |  |
|  | % within column | |  | 51.1 % |  | 74.4 % |  | 79.5 % |  | 67.4 % |  |
| White Irish |  | Observed |  | 3 |  | 1 |  | 0 |  | 4 |  |
|  | % within row | |  | 75.0 % |  | 25.0 % |  | 0.0 % |  | 100.0 % |  |
|  | % within column | |  | 6.4 % |  | 2.3 % |  | 0.0 % |  | 3.1 % |  |
| Total |  | Observed |  | 47 |  | 43 |  | 39 |  | 129 |  |
|  | % within row | |  | 36.4 % |  | 33.3 % |  | 30.2 % |  | 100.0 % |  |
|  | % within column | |  | 100.0 % |  | 100.0 % |  | 100.0 % |  | 100.0 % |  |

| *Table L4*. Descriptive statistics of cohabitation by group | | | | | | | | | | | |
| --- | --- | --- | --- | --- | --- | --- | --- | --- | --- | --- | --- |
|  | | | | **Groups** | | | | | |  | |
| **Cohabitation** | |  | | **H&H** | | **HD** | | **HM** | | **Total** | |
| Halfway / group home |  | Observed |  | 4 |  | 0 |  | 2 |  | 6 |  |
|  | | % within row |  | 66.7 % |  | 0.0 % |  | 33.3 % |  | 100.0 % |  |
|  | | % within column |  | 8.5 % |  | 0.0 % |  | 5.1 % |  | 4.7 % |  |
| Homeless / shelter / couch surfing |  | Observed |  | 18 |  | 0 |  | 21 |  | 39 |  |
|  | | % within row |  | 46.2 % |  | 0.0 % |  | 53.8 % |  | 100.0 % |  |
|  | | % within column |  | 38.3 % |  | 0.0 % |  | 53.8 % |  | 30.2 % |  |
| Live alone |  | Observed |  | 15 |  | 15 |  | 12 |  | 42 |  |
|  | | % within row |  | 35.7 % |  | 35.7 % |  | 28.6 % |  | 100.0 % |  |
|  | | % within column |  | 31.9 % |  | 34.9 % |  | 30.8 % |  | 32.6 % |  |
| Other |  | Observed |  | 2 |  | 6 |  | 0 |  | 8 |  |
|  | | % within row |  | 25.0 % |  | 75.0 % |  | 0.0 % |  | 100.0 % |  |
|  | | % within column |  | 4.3 % |  | 14.0 % |  | 0.0 % |  | 6.2 % |  |
| With flatmates |  | Observed |  | 1 |  | 2 |  | 0 |  | 3 |  |
|  | | % within row |  | 33.3 % |  | 66.7 % |  | 0.0 % |  | 100.0 % |  |
|  | | % within column |  | 2.1 % |  | 4.7 % |  | 0.0 % |  | 2.3 % |  |
| With own children |  | Observed |  | 2 |  | 5 |  | 2 |  | 9 |  |
|  | | % within row |  | 22.2 % |  | 55.6 % |  | 22.2 % |  | 100.0 % |  |
|  | | % within column |  | 4.3 % |  | 11.6 % |  | 5.1 % |  | 7.0 % |  |
| With parents |  | Observed |  | 0 |  | 3 |  | 0 |  | 3 |  |
|  | | % within row |  | 0.0 % |  | 100.0 % |  | 0.0 % |  | 100.0 % |  |
|  | | % within column |  | 0.0 % |  | 7.0 % |  | 0.0 % |  | 2.3 % |  |
| With partner |  | Observed |  | 1 |  | 2 |  | 2 |  | 5 |  |
|  | | % within row |  | 20.0 % |  | 40.0 % |  | 40.0 % |  | 100.0 % |  |
|  | | % within column |  | 2.1 % |  | 4.7 % |  | 5.1 % |  | 3.9 % |  |
| With siblings |  | Observed |  | 1 |  | 0 |  | 0 |  | 1 |  |
|  | | % within row |  | 100.0 % |  | 0.0 % |  | 0.0 % |  | 100.0 % |  |
|  | | % within column |  | 2.1 % |  | 0.0 % |  | 0.0 % |  | 0.8 % |  |
| With spouse / common law partner |  | Observed |  | 3 |  | 10 |  | 0 |  | 13 |  |
|  | | % within row |  | 23.1 % |  | 76.9 % |  | 0.0 % |  | 100.0 % |  |
|  | | % within column |  | 6.4 % |  | 23.3 % |  | 0.0 % |  | 10.1 % |  |
| Total |  | Observed |  | 47 |  | 43 |  | 39 |  | 129 |  |
|  | | % within row |  | 36.4 % |  | 33.3 % |  | 30.2 % |  | 100.0 % |  |
|  | | % within column |  | 100.0 % |  | 100.0 % |  | 100.0 % |  | 100.0 % |  |

| *Table L5*. Descriptive statistics of marital status by group | | | | | | | | | | | | |
| --- | --- | --- | --- | --- | --- | --- | --- | --- | --- | --- | --- | --- |
|  | | | | | **Groups** | | | | | |  | |
| **Marital status** |  | | | | **H&H** | | **HD** | | **HM** | | **Total** | |
| Common-law marriage | |  | Observed |  | 0 |  | 2 |  | 1 |  | 3 |  |
|  | % within row | | |  | 0.0 % |  | 66.7 % |  | 33.3 % |  | 100.0 % |  |
|  | % within column | | |  | 0.0 % |  | 4.7 % |  | 2.6 % |  | 2.3 % |  |
| Divorced | |  | Observed |  | 3 |  | 4 |  | 1 |  | 8 |  |
|  | % within row | | |  | 37.5 % |  | 50.0 % |  | 12.5 % |  | 100.0 % |  |
|  | % within column | | |  | 6.4 % |  | 9.3 % |  | 2.6 % |  | 6.2 % |  |
| In a relationship (not married but have a partner / girlfriend / boyfriend) | |  | Observed |  | 6 |  | 6 |  | 5 |  | 17 |  |
|  | % within row | | |  | 35.3 % |  | 35.3 % |  | 29.4 % |  | 100.0 % |  |
|  | % within column | | |  | 12.8 % |  | 14.0 % |  | 12.8 % |  | 13.2 % |  |
| Married | |  | Observed |  | 5 |  | 13 |  | 0 |  | 18 |  |
|  | % within row | | |  | 27.8 % |  | 72.2 % |  | 0.0 % |  | 100.0 % |  |
|  | % within column | | |  | 10.6 % |  | 30.2 % |  | 0.0 % |  | 14.0 % |  |
| Separated | |  | Observed |  | 3 |  | 1 |  | 3 |  | 7 |  |
|  | % within row | | |  | 42.9 % |  | 14.3 % |  | 42.9 % |  | 100.0 % |  |
|  | % within column | | |  | 6.4 % |  | 2.3 % |  | 7.7 % |  | 5.4 % |  |
| Single (no partner) | |  | Observed |  | 29 |  | 17 |  | 29 |  | 75 |  |
|  | % within row | | |  | 38.7 % |  | 22.7 % |  | 38.7 % |  | 100.0 % |  |
|  | % within column | | |  | 61.7 % |  | 39.5 % |  | 74.4 % |  | 58.1 % |  |
| Widowed | |  | Observed |  | 1 |  | 0 |  | 0 |  | 1 |  |
|  | % within row | | |  | 100.0 % |  | 0.0 % |  | 0.0 % |  | 100.0 % |  |
|  | % within column | | |  | 2.1 % |  | 0.0 % |  | 0.0 % |  | 0.8 % |  |
| Total | |  | Observed |  | 47 |  | 43 |  | 39 |  | 129 |  |
|  | % within row | | |  | 36.4 % |  | 33.3 % |  | 30.2 % |  | 100.0 % |  |
|  | % within column | | |  | 100.0 % |  | 100.0 % |  | 100.0 % |  | 100.0 % |  |

| *Table L6*. Descriptive statistics of educational level by group | | | | | | | | | | | | |
| --- | --- | --- | --- | --- | --- | --- | --- | --- | --- | --- | --- | --- |
|  | | | | | **Groups** | | | | | |  | |
| **Educational level** | | |  | | **H&H** | | **HD** | | **HM** | | **Total** | |
| Doctorate (e.g. PhD, EdD, DPhill) | |  | Observed |  | 0 |  | 0 |  | 1 |  | 1 |  |
|  | % within row | | |  | 0.0 % |  | 0.0 % |  | 100.0 % |  | 100.0 % |  |
|  | % within column | | |  | 0.0 % |  | 0.0 % |  | 2.6 % |  | 0.8 % |  |
| Higher Degree (e.g. MA, MSc, PGCE) | |  | Observed |  | 3 |  | 10 |  | 7 |  | 20 |  |
|  | % within row | | |  | 15.0 % |  | 50.0 % |  | 35.0 % |  | 100.0 % |  |
|  | % within column | | |  | 6.4 % |  | 23.3 % |  | 17.9 % |  | 15.5 % |  |
| Highers / A-levels / BTEC | |  | Observed |  | 8 |  | 6 |  | 8 |  | 22 |  |
|  | % within row | | |  | 36.4 % |  | 27.3 % |  | 36.4 % |  | 100.0 % |  |
|  | % within column | | |  | 17.0 % |  | 14.0 % |  | 20.5 % |  | 17.1 % |  |
| National Vocational Qualification | |  | Observed |  | 5 |  | 4 |  | 2 |  | 11 |  |
|  | % within row | | |  | 45.5 % |  | 36.4 % |  | 18.2 % |  | 100.0 % |  |
|  | % within column | | |  | 10.6 % |  | 9.3 % |  | 5.1 % |  | 8.5 % |  |
| Other postgraduate qualification | |  | Observed |  | 2 |  | 4 |  | 0 |  | 6 |  |
|  | % within row | | |  | 33.3 % |  | 66.7 % |  | 0.0 % |  | 100.0 % |  |
|  | % within column | | |  | 4.3 % |  | 9.3 % |  | 0.0 % |  | 4.7 % |  |
| Prefer not to say | |  | Observed |  | 2 |  | 1 |  | 0 |  | 3 |  |
|  | % within row | | |  | 66.7 % |  | 33.3 % |  | 0.0 % |  | 100.0 % |  |
|  | % within column | | |  | 4.3 % |  | 2.3 % |  | 0.0 % |  | 2.3 % |  |
| SATs (Standardised Assessment Tests – Primary School) | |  | Observed |  | 2 |  | 0 |  | 2 |  | 4 |  |
|  | % within row | | |  | 50.0 % |  | 0.0 % |  | 50.0 % |  | 100.0 % |  |
|  | % within column | | |  | 4.3 % |  | 0.0 % |  | 5.1 % |  | 3.1 % |  |
| Standard grades / GCSE / O-levels | |  | Observed |  | 14 |  | 4 |  | 13 |  | 31 |  |
|  | % within row | | |  | 45.2 % |  | 12.9 % |  | 41.9 % |  | 100.0 % |  |
|  | % within column | | |  | 29.8 % |  | 9.3 % |  | 33.3 % |  | 24.0 % |  |
| Undergraduate degree (e.g. BA, BSc) | |  | Observed |  | 8 |  | 12 |  | 3 |  | 23 |  |
|  | % within row | | |  | 34.8 % |  | 52.2 % |  | 13.0 % |  | 100.0 % |  |
|  | % within column | | |  | 17.0 % |  | 27.9 % |  | 7.7 % |  | 17.8 % |  |
| Unknown | |  | Observed |  | 3 |  | 2 |  | 3 |  | 8 |  |
|  | % within row | | |  | 37.5 % |  | 25.0 % |  | 37.5 % |  | 100.0 % |  |
|  | % within column | | |  | 6.4 % |  | 4.7 % |  | 7.7 % |  | 6.2 % |  |
| Total | |  | Observed |  | 47 |  | 43 |  | 39 |  | 129 |  |
|  | % within row | | |  | 36.4 % |  | 33.3 % |  | 30.2 % |  | 100.0 % |  |
|  | % within column | | |  | 100.0 % |  | 100.0 % |  | 100.0 % |  | 100.0 % |  |

| *Table L7*. Descriptive statistics of employment status by group | | | | | | | | | | | | | |
| --- | --- | --- | --- | --- | --- | --- | --- | --- | --- | --- | --- | --- | --- |
|  | | | | | | **Groups** | | | | | |  | |
| **Employment status** | | | |  | | **H&H** | | **HD** | | **HM** | | **Total** | |
| Employed full-time | |  | Observed | |  | 5 |  | 14 |  | 6 |  | 25 |  |
|  | % within row | | | |  | 20.0 % |  | 56.0 % |  | 24.0 % |  | 100.0 % |  |
|  | % within column | | | |  | 10.6 % |  | 32.6 % |  | 15.4 % |  | 19.4 % |  |
| Employed part-time | |  | Observed | |  | 5 |  | 6 |  | 2 |  | 13 |  |
|  | % within row | | | |  | 38.5 % |  | 46.2 % |  | 15.4 % |  | 100.0 % |  |
|  | % within column | | | |  | 10.6 % |  | 14.0 % |  | 5.1 % |  | 10.1 % |  |
| Retired | |  | Observed | |  | 3 |  | 11 |  | 1 |  | 15 |  |
|  | % within row | | | |  | 20.0 % |  | 73.3 % |  | 6.7 % |  | 100.0 % |  |
|  | % within column | | | |  | 6.4 % |  | 25.6 % |  | 2.6 % |  | 11.6 % |  |
| Stay at home parent | |  | Observed | |  | 1 |  | 4 |  | 0 |  | 5 |  |
|  | % within row | | | |  | 20.0 % |  | 80.0 % |  | 0.0 % |  | 100.0 % |  |
|  | % within column | | | |  | 2.1 % |  | 9.3 % |  | 0.0 % |  | 3.9 % |  |
| Student | |  | Observed | |  | 1 |  | 0 |  | 1 |  | 2 |  |
|  | % within row | | | |  | 50.0 % |  | 0.0 % |  | 50.0 % |  | 100.0 % |  |
|  | % within column | | | |  | 2.1 % |  | 0.0 % |  | 2.6 % |  | 1.6 % |  |
| Unemployed and seeking work | |  | Observed | |  | 13 |  | 1 |  | 18 |  | 32 |  |
|  | % within row | | | |  | 40.6 % |  | 3.1 % |  | 56.3 % |  | 100.0 % |  |
|  | % within column | | | |  | 27.7 % |  | 2.3 % |  | 46.2 % |  | 24.8 % |  |
| Unemployed due to disability / incapacity | |  | Observed | |  | 18 |  | 7 |  | 10 |  | 35 |  |
|  | % within row | | | |  | 51.4 % |  | 20.0 % |  | 28.6 % |  | 100.0 % |  |
|  | % within column | | | |  | 38.3 % |  | 16.3 % |  | 25.6 % |  | 27.1 % |  |
| Unknown | |  | Observed | |  | 1 |  | 0 |  | 1 |  | 2 |  |
|  | % within row | | | |  | 50.0 % |  | 0.0 % |  | 50.0 % |  | 100.0 % |  |
|  | % within column | | | |  | 2.1 % |  | 0.0 % |  | 2.6 % |  | 1.6 % |  |
| Total | |  | Observed | |  | 47 |  | 43 |  | 39 |  | 129 |  |
|  | % within row | | | |  | 36.4 % |  | 33.3 % |  | 30.2 % |  | 100.0 % |  |
|  | % within column | | | |  | 100.0 % |  | 100.0 % |  | 100.0 % |  | 100.0 % |  |

| *Table L8*. Descriptive statistics of self-report early experiences of material deprivation by group | | | | | | | | | | | |
| --- | --- | --- | --- | --- | --- | --- | --- | --- | --- | --- | --- |
| **Early experiences of material deprivation?** | |  | | **Groups** | | | | | |  | |
|  |  |  | | **H&H** | | **HD** | | **HM** | | **Total** | |
| No |  | Observed |  | 15 |  | 24 |  | 21 |  | 60 |  |
|  | | % within row |  | 25.0 % |  | 40.0 % |  | 35.0 % |  | 100.0 % |  |
|  | | % within column |  | 31.9 % |  | 57.1 % |  | 53.8 % |  | 46.9 % |  |
| Yes |  | Observed |  | 32 |  | 18 |  | 18 |  | 68 |  |
|  | | % within row |  | 47.1 % |  | 26.5 % |  | 26.5 % |  | 100.0 % |  |
|  | | % within column |  | 68.1 % |  | 42.9 % |  | 46.2 % |  | 53.1 % |  |
| Total |  | Observed |  | 47 |  | 42 |  | 39 |  | 128 |  |
|  | | % within row |  | 36.7 % |  | 32.8 % |  | 30.5 % |  | 100.0 % |  |
|  | | % within column |  | 100.0 % |  | 100.0 % |  | 100.0 % |  | 100.0 % |  |
